# A Base‐Mediated Rearrangement of the Benzylic 1,5‐Hexadipyridynyl Moiety

**DOI:** 10.1002/cplu.202500252

**Published:** 2025-08-04

**Authors:** Wouter A. Remmerswaal, Thai‐Tony Nguyen, Zijian Han, Fedor Krasovski Slobodian, Ruisheng Xiong, Vadim Kessler, Zhijian Xu, Weiliang Zhu, Mate Erdelyi

**Affiliations:** ^1^ Department of Chemistry − BMC Uppsala University 751 23 Uppsala Sweden; ^2^ Center of Excellence for the Chemical Mechanisms of Life Uppsala University SE‐751 23 Uppsala Sweden; ^3^ State Key Laboratory of Drug Research Drug Discovery and Design Center Shanghai Institute of Materia Medica Chinese Academy of Sciences Shanghai 201203 China; ^4^ Department of Molecular Sciences BioCenter Swedish University of Agricultural Sciences 750 07 Uppsala Sweden

**Keywords:** density functional theory, mechanism, reactivity, rearrangement

## Abstract

A previously unrecognized base‐mediated rearrangement of a benzylic 1,5‐hexadipyridynyl moiety is reported. Upon exposure to base, this structural motif rearranges into a constrained vinyl‐pyridine substituted cyclobutene. Computational modeling indicates that the rearrangement takes place following a route involving stepwise deprotonation, shifted reprotonation, and 4π‐electrocyclization. The reaction rate and the stereochemical outcome is consistent with the experimental observations. Furthermore, nonbase mediates rearrangements, through well‐known Cope‐like [3,3]‐sigmatropic shifts, are found to be high in energy, and therefore, take a backseat to the base‐mediated pathway. This rearrangement may provide a novel reactivity pathway of conjugated systems for synthetic methodology development.

## Graphical abstract

**A rearrangement unheard of!** When exposed to base, benzylic 1,5‐hexadipyridynyl moieties skip a beat—disregarding Cope‐like rearrangement and instead readily rearrange through a deprotonation–reprotonation–electrocyclization cascade. The result is a highly rigid vinyl‐pyridine cyclobutene, fashioned via a novel pathway.

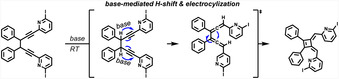

## 1 Introduction

**Scheme 1:**
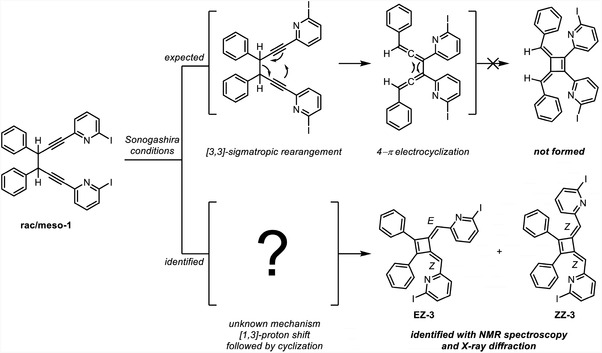
Under Sonogashira coupling conditions, a 1,5‐hexadiyne moiety containing benzyl and pyridine‐ethynyl functionalities isomerizes into a mixture of [1,3]‐proton shifted products *EZ*‐**3** and *ZZ*‐**3**, but not into **2**.

In the synthesis toward supramolecular scaffolds,^[^ ^1^ ^]^ we observed that the Sonogashira coupling of hexa‐1,5‐diyne‐3,4‐diyldibenzene^[^ ^2^, ^3^, ^4^, ^5^ ^]^ and 2,6‐diiodopyridine led to the formation of unexpected byproducts along with the anticipated di(iodopyridinyl) building block **1** (**Scheme** **1**). The possibility of building block **1** undergoing a [3,3]‐sigmatropic rearrangement followed by a 4π‐electrocyclization reaction^[^ ^6^, ^7^ ^]^ to yield byproduct **2** was expected.^[^ ^8^, ^9^, ^10^, ^11^, ^12^, ^13^, ^14^, ^15^ ^]^ However, the isolated products were not compatible with the expected outcome of the above described rearrangements, but rather with the isomeric products ***EZ*‐3** and ***ZZ*‐3**, the structure of which were identified by single crystal X‐ray diffraction and nuclear magnetic resonance (NMR) spectroscopy. As this type of rearrangement has not yet been reported, we performed a combined spectroscopic and computational investigation of its mechanism.

## 2 Results and Discussion

**Scheme 2:**
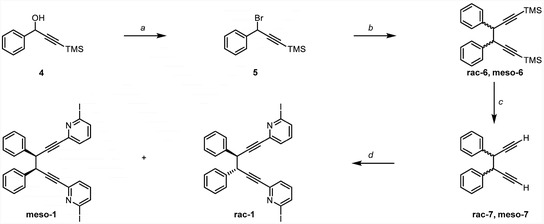
Synthesis of **1**, as an isomeric mixture of **rac‐1** and **meso‐1.** *Reagents and conditions*: a) PBr_3_ (1.5 equiv), Et_2_O, 2 h, 0•°C—RT, **5**: used as a crude mixture in further reactions; b) Mg turnings (1.5 equiv), Fe(acac)_3_ (2 mol%), dry THF, 17 h, RT, **rac‐6**/**meso‐6** (2:1): 57% over two steps; c) KF (7 equiv), MeOH, 17 h, 55 °C, **rac‐7**/**meso‐7** (2:1): 89%; d) 2,6‐Diiodopyridine (2.2 equiv), PdCl_2_(PPh_3_)_2_ (10•mol%), CuI (5 mol%), NEt_3_ (10 equiv.), dry DMF, 30 min, 40 °C: **meso‐1**/**rac‐1, 7%, and *EZ*‐3/*ZZ*−3 in a ratio of 1:2**.

The benzylic 1,5‐hexadipyridynyl moiety **1** (**Scheme** **2**) was synthesized from 1‐phenyl‐3‐(trimethylsilyl)prop‐2‐yn‐1‐ol **4**. Following bromination using PBr_3_, yielding **5**, the chiral building block **6** was formed by the iron‐catalyzed homocoupling of **5**.^[^ ^16^ ^]^ Subsequently, the trimethylsilyl groups were deprotected using KF to yield **7** as a mixture of racemic (**rac‐7**) and meso‐isomers (**meso‐7**). **Meso‐7** was isolated by crystallization from the isomeric mixture, simultaneously enriching **rac‐7** (1:2 meso to racemate). The Sonogashira coupling of the di‐alkyne building block **7** and 2,6‐diiodopyridine yielded **rac‐1** and **meso‐1** (optimization of the reaction protocol is depicted in Figure S1, Supporting Information) along with the byproducts ***EZ*‐3** and ***ZZ*‐3**.

**Scheme 3:**
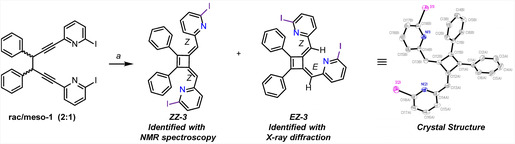
Subjecting **rac‐1** and **meso‐1** to conditions resembling a Sonogashira coupling provides ***EZ*‐3** and ***ZZ*‐3. *EZ*‐3** was crystallized and analyzed with X‐ray crystallography (ellipsoid displacement probability = 30%, CCDC 2,429,445). *Reagents and condition*s: a) PdCl_2_(PPh_3_), CuI, NEt_3_ (10 equiv), dry DMF, 180 min, 25 °C, quantitative as determined with NMR (Figure S3, Supporting Information).

When **rac‐1** was subjected to conditions resembling a Sonogashira coupling (**Scheme** **3**), ***EZ*‐3** and ***ZZ*‐3** were formed in a 1:1.5 ratio, and were isolated by flash column chromatography and high‐performance liquid chromatography. The ***EZ*‐3** isomer crystallized from a mixture of **rac/meso**‐**7** (2:1) and was identified by single crystal X‐ray analysis (Scheme 3, see Figure S27 and Table S1–S8, Supporting Information for further details) and NMR spectroscopy. The second stereoisomer was identified as ***ZZ* *‐*3** with NMR spectroscopy. Upon treatment of **meso‐1** under the same reaction conditions, ***EZ*‐3** and ***ZZ*‐3** formed in a 5:1 ratio (Figure S2, Supporting Information).

**Figure 1:**
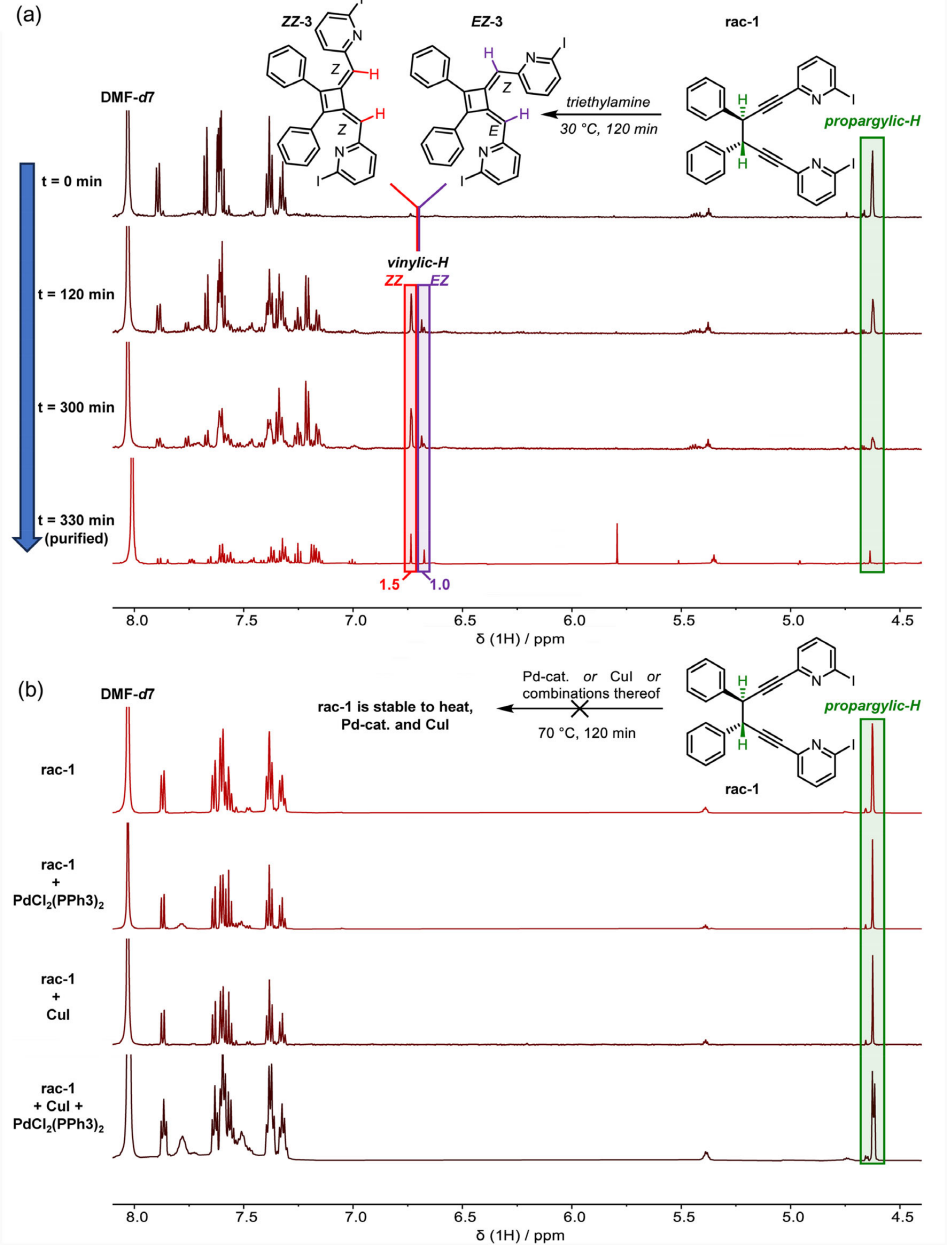
a) Base‐driven formation of ***ZZ*‐3** and ***EZ*‐3** from **rac‐1**, monitored over time with NMR spectroscopy. b) Stability of **rac‐1** when exposed to PdCl_2_(PPh_3_), CuI, or both at 70 °C for 120 min.

The formation of ***EZ*‐3** and ***ZZ*‐3** over time from **rac/meso‐1** was monitored with NMR spectroscopy indicating that neither increased temperature, nor the presence of copper(I) iodide or PdCl_2_(PPh_3_)_2_, or combinations thereof led to any conversion of **rac‐1–** **3** (**Figure** **1**b). A radical mechanism was ruled out by carrying out the reaction in the presence of TEMPO (Figure S3, Supporting Information). In our hands, ***EZ*‐3** and ***ZZ*‐3** were readily formed in the presence of triethylamine at elevated temperatures (Figure 1a). NMR measurements showed that both **rac‐1** and **meso‐1** converted nearly quantitively to ***EZ*‐3**/***ZZ*−3 (**Figure S4, Supporting Information**).**

**Figure 2:**
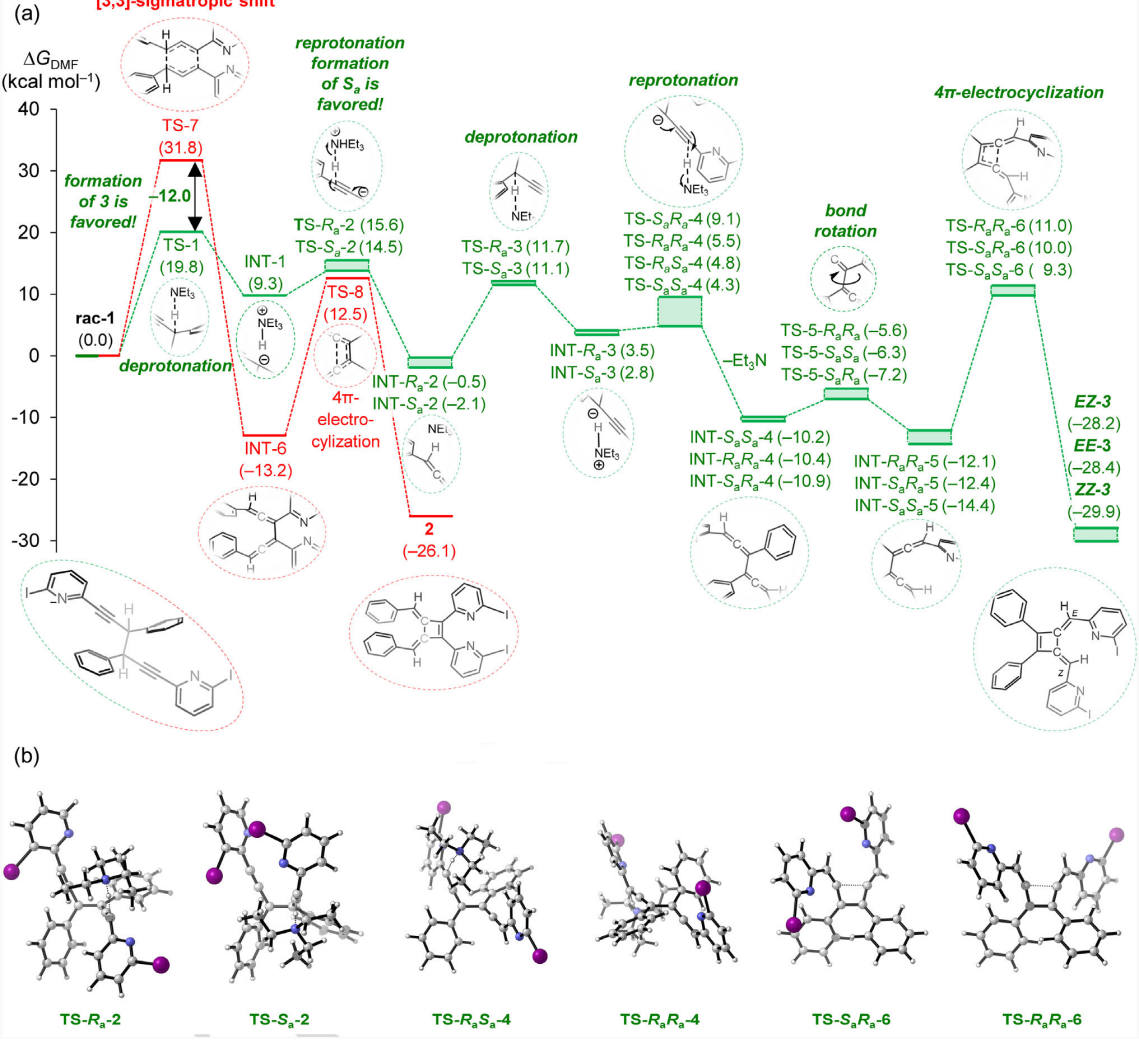
a) The computed reaction profiles for the formation of ***EZ*‐3** and ***ZZ*‐3** from **rac‐1** (green path) and for the formation of **2** (red path), and b) cylview^[^ ^37^ ^]^ images of transition states critical for the stereochemical outcome. Gibbs free solute energies relative to **rac‐1** (Δ*G*_DMF_) are given in kcal mol^–1^. See Table S9 and S10, Supporting Information, for energies and coordinates. Computed stationary points are given relative to the solvent separated reactants (**rac‐1** and triethylamine), and were computed at SMD‐18(DMF)/M06‐2X‐D3/6‐311++G(d,p)/SDD//‐PCM(DMF)//M06‐2X‐D3/6‐31G(d)/SDD.^[^ ^17^, ^18^, ^19^, ^20^, ^21^ ^]^

The mechanism for forming ***EZ*‐3** and ***ZZ*‐3** from **rac‐1** was investigated by computing the potential energy surfaces for possible reaction pathways (**Figure** **2**) at the SMD‐18(DMF)/M06‐2X‐D3/6‐311++G(d,p)/SDD//PCM(DMF)‐M06‐2X‐D3/6‐31G(d)/SDD^[^ ^17^, ^18^, ^19^, ^20^, ^21^, ^22^ ^]^ level of theory using Gaussian 16.^[^ ^23^ ^]^ This level of theory has previously provided good experimental correlation for the rearrangement reactions of unsaturated nitriles, and is expected to give similarly accurate energies for our systems.^[^ ^24^ ^]^

**Scheme 4:**
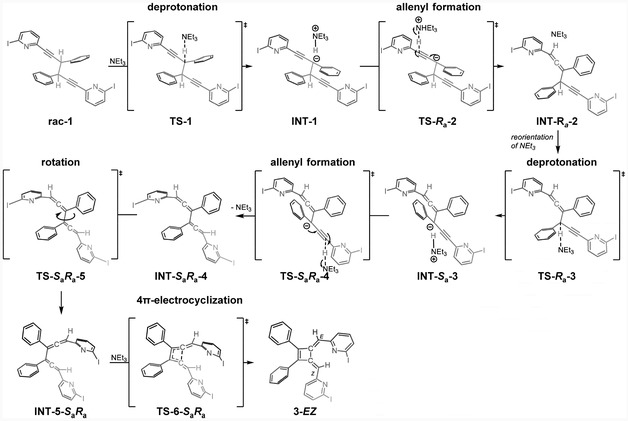
The trimethylamine‐mediated formation ***EZ*‐3** from **rac‐1.** The analogous formation of ***EE*‐3** and ***ZZ*‐3** isomers is not shown here, for clarity; however, it follows the same logic.

As experiments (Figure 1) revealed that the rearrangement of **rac‐1** into ***EZ*‐3** and ***ZZ*‐3** is base‐dependent, we computed the potential energy surface for a triethylamine‐mediated reaction route (**Scheme** **4** and Figure 2). Owing to the formation of two stereocenters, four analogous pathways are possible. In the following description, we focus on the key stereochemistry‐ and rate‐determining steps, whereas give full energy profiles in Figure 2.

The reaction is initiated by abstraction of a propargylic proton by triethylamine via transition state **TS‐1** (Δ*G* = 19.8 kcal mol^–1^), forming the charge‐separated intermediate **INT‐1**. This is the overall‐rate‐determining step of the base‐mediated rearrangement that is facilitated by the acidity of the propargylic position,^[^ ^25^, ^26^, ^27^, ^28^ ^]^ and further by an alkyne substituent (Table S11, Supporting Information).

The triethylammonium ion then transfers the abstracted proton toward the terminal end of the alkyn, forming the allenylic position. At this stage, the *E* or *Z* configuration of the first vinylic position in the final product is defined through **TS‐*R***_**a**_ **‐2** and **TS‐*S***_**a**_ **‐2**. The lower barrier of **TS‐*S***_***a***_ ***‐2***, ΔΔ*G*_TS‐*Ra‐*2‐TS‐*Sa*‐2_ = +1.1 kcal mol^–1^, predominantly favors formation of the *Z*‐configured intermediate **INT‐*S***_**a**_ **‐2**. The process is then repeated, and hence, the second propargylic proton is abstracted, followed by allenylic reprotonation defining the second stereocenter. The reprotonation transition states,^[^ ^29^ ^]^ **TS‐*R***_**a**_ ***R***_**a**_ **‐4** and **TS‐*R***_**a**_ **S**_**a**_ **‐4**, are near isoenergetic (ΔΔ*G*_TS‐*RaRa‐*4‐TS‐*RaSa*‐4_ = +0.7 kcal mol^–1^), forming a mixture of ***R***_**a**_ ***R***_**a**_‐ and ***R***_**a**_ ***S***_**a**_‐configured allenylic intermediates (**INT‐4**) upon dissociation of the triethylamine complex. These intermediates are thermodynamically favored (Δ*G* ≈ −10 kcal mol^−1^), due to the electron‐withdrawing iodopyridine ring and formation of a conjugated system. Thus, the thermodynamic driving force of the reaction is the energy gain, Δ*G* ≈ −10 kcal mol^−1^, which in association with the low energy barrier of the proton abstraction, Δ*G* = 19.8 kcal mol^−1^, and makes the transformation feasible.

Following formation of dienes **INTs‐4**, conformational rotation to **INTs‐5** via **TSs‐5** enables orbital alignment necessary for the ensuing 4*π*‐ electrocyclization via **TS‐*R***_**a**_ ***R***_**a**_ **‐6** and **TS‐*S***_**a**_ ***R***_**a**_ **‐6**. For the formation of the second stereocenter, these ring‐forming steps are rate‐determining (Δ*G* = 11.0 and 10.0 kcal mol^−1^, respectively). These energy barriers are in line with the analogous 4π‐electrocyclizations of substituted *bis*‐allenes, as reported by Pasto and coworkers,^[^ ^30^ ^,^ ^31^ ^]^ which occur readily at room temperature and are involved in the formation of alkylidenecyclobutenes.^[^ ^32^ ^,^ ^33^ ^]^ This cyclization step shows a computed ΔΔ*G*_TS‐*RaRa‐*6‐TS‐*Sara*‐6_ = +1.0 kcal mol^–1^, suggesting a mild preference toward formation of ***EZ*‐3**, but ultimately favors forming mixtures of ***EZ*‐3** and ***ZZ*‐3**. The formation of a mixture of isomers is consistent with the experiment.

Overall, the stereochemistry of **3** is established during the two allenyl‐forming steps **TS‐2** and **TS‐4**. The first favors the *S*_*a*_‐isomer, providing a *Z*‐configured vinyl after cyclization, while the latter shows little stereochemical bias. As a result, the formation of a mixture of ***ZZ*‐3** and ***EZ*‐3** isomers is predicted, which is consistent with the experimental observation (Figure 1). The computed activation energy, Δ*G* = 19.8 kcal mol^−1^, aligns with the multihours experimental half‐life of **rac‐1** at room temperature.

**Scheme 5:**
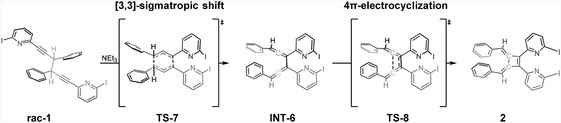
Schematic representation of the computed base‐mediated formation of **2** from **rac‐1**.

Finally, we examined the formation of byproduct **2** (Scheme 3), which could be expected based on literature reports, however, was not observed. We computed the potential energy surface towards the formation of **2** via a Cope‐like [3,3]‐sigmatropic rearrangement (**TS‐7**, Δ*G* = 31.8 kcal mol^–1^) of **rac‐1** to the highly stable intermediate **INT‐6** (Δ*G* = −13.29 kcal mol^–1^) (**Scheme** **5**). This rate‐determining step is followed by 4π‐electrocyclization via **TS‐8** (Δ*G* = 12.5 kcal mol^–1^), yielding **2**. The high‐energy barrier of **TS‐7** is consistent with typical pericyclic reaction energetics.^[^ ^11^ ^,^ ^34^, ^35^, ^36^ ^]^ The rigid structure of propargylic centers, as in **rac‐1,** hinders the alignment of the alkyne pyridinyl carbons. To achieve the required orbital overlap for the pericyclic reaction, the propargylic carbons dissociate en route to **TS‐8,** positioning the C•••C_propargylic_ and C•••C_alkyne_ bonds in a semi‐equidistant fashion. Here, the C•••C_propargylic_ and C•••C_alkyne_ distances are 2.23 and 2.30 Å, respectively, similar to those reported for a 1,5‐diyne systems by Wu et al.^[^ ^10^ ^]^ Thus, distorting the geometry of **rac‐1** to reach **TS‐8** is energetically penalized (Δ*G* = 31.8 kcal mol^–1^). Comparable barriers have been reported for the [3,3]‐sigmatropic rearrangement of 1,5‐hexadiyne by Houk,^[^ ^36^ ^]^ and experimentally by Huntsman.^[^ ^15^ ^]^ Overall, the significantly higher activation energy of the route leading to **2** as compared to that toward **3**, ΔΔ*G*_**TS7**–**TS1**_ = +12.0 kcal mol^−1^, suggests that the former is unlikely to form. This aligns with our experimental observations (Scheme 3).

## 3 Conclusions

We have identified a previously unknown rearrangement of the benzylic 1,5‐hexadipyridynyl moiety into a substituted cyclobutene. The transformation proceeds through a base‐mediated mechanism, yielding a mixture of the *EZ* and *ZZ* stereoisomers. Computations on the density functional theory level are in agreement with a base‐mediated step‐wise mechanism, where the initial deprotonation of **rac/meso‐1** is the rate‐determining step. This is followed by protonation and a subsequent 4π‐electrocyclization to yield the observed rearrangement products. Computed barriers are consistent with the experimental reaction rate and stereochemical outcome, that is, the formation of a *EZ*/*ZZ*‐mixture and the absence of the *EE* isomer. The absence of products resulting from a nonbase‐mediated mechanism correlate well with the computed high‐energy barrier for a Cope‐like [3,3]‐sigmatropic shift. This base‐mediated rearrangement provides a so far unexplored reactivity pathway for a benzylic 1,5‐hexadipyridynyl system that may gain applicability in structurally related conjugated systems.

## Conflict of Interest

The authors declare no conflict of interest.

## Supporting information

Supplementary Material

## Data Availability

The data that support the findings of this study are openly available in [Zenodo] at [https://doi.org/10.5281/zenodo.15132431], reference number [15132431].

## References

[1] A. Vanderkooy , A. K. Gupta , T. Földes , S. Lindblad , A. Orthaber , I. Pápai , M. Erdélyi , Angew. Chem. Int. Ed. 2019, 58, 9012. 10.1002/anie.201904817 PMC6773207 31074942

[2] S. Padmanabhan , K. M. Nicholas , J. Organomet. Chem. 1981, 212, 115.

[3] H.‐L. Sun , B. Wu , D.‐Q. Liu , Z.‐D. Yu , J.‐J. Wang , Q. Liu , X. Liu , D. Niu , J.‐H. Dou , R. Zhu , J. Am. Chem. Soc. 2022, 144, 8807. 35522220 10.1021/jacs.2c02816

[4] G. G. Melikyan , R. Davis , S. Cappuccino , Organometallics 2016, 35, 2854.

[5] G. G. Melikyan , R. C. Combs , J. Lamirand , M. Khan , K. M. Nicholas , Tetrahedron Lett. 1994, 35, 363.

[6] H. Hauptmann , Tetrahedron Lett. 1974, 15, 3589.

[7] H. Hauptmann , Tetrahedron Lett. 1975, 16, 1931.

[8] J. D. Savee , B. Sztáray , P. Hemberger , J. Zádor , A. Bodi , D. L. Osborn , Faraday Discuss. 2022, 238, 645. 35822493 10.1039/d2fd00028h

[9] H. Hopf , G. Markopoulos , Beilstein J. Org. Chem. 2012, 8, 1936. 23209534 10.3762/bjoc.8.225 PMC3511034

[10] Y. Xia , F. Zhou , Y. Li , W. Li , J. Mol. Struct. THEOCHEM. 2009, 904, 69.

[11] K. A. Black , S. Wilsey , K. N. Houk , J. Am. Chem. Soc. 1998, 120, 5622.

[12] G. Zimmermann , M. Nüchter , M. Remmler , M. Findeisen , H. Hopf , L. Ernst , C. Mlynek , Chem. Ber. 1994, 127, 1747.

[13] H. Hopf , U. Hamann , G. Zimmermann , M. Remmler , Chem. Ber. 1994, 127, 959.

[14] T. J. Henry , R. G. Bergman , J. Am. Chem. Soc. 1972, 94, 5103.

[15] W. D. Huntsman , H. J. Wristers , J. Am. Chem. Soc. 1967, 89, 342.

[16] X. Xu , D. Cheng , W. Pei , J. Org. Chem. 2006, 71, 6637. 16901160 10.1021/jo060673l

[17] A. V. Marenich , C. J. Cramer , D. G. Truhlar , J. Phys. Chem. B 2009, 113, 6378. 19366259 10.1021/jp810292n

[18] R. F. Ribeiro , A. V. Marenich , C. J. Cramer , D. G. Truhlar , J. Phys. Chem. B 2011, 115, 14556. 21875126 10.1021/jp205508z

[19] Y. Zhao , D. G. Truhlar , Theor. Chem. Acc. 2008, 120, 215.

[20] S. Grimme , J. Antony , S. Ehrlich , H. Krieg , J. Chem. Phys. 2010, 132, 154104. 20423165 10.1063/1.3382344

[21] S. Grimme , S. Ehrlich , L. Goerigk , J. Comput. Chem. 2011, 32, 1456. 21370243 10.1002/jcc.21759

[22] E. Engelage , N. Schulz , F. Heinen , S. M. Huber , D. G. Truhlar , C. J. Cramer , Chem.—Eur. J. 2018, 24, 15983. 30152113 10.1002/chem.201803652

[23] M. J. Frisch , G. W. Trucks , H. B. Schlegel , G. E. Scuseria , M. A. Robb , J. R. Cheeseman , G. Scalmani , V. Barone , G. A. Petersson , H. Nakatsuji , X. Li , M. Caricato , A. V. Marenich , J. Bloino , B. G. Janesko , R. Gomperts , B. Mennucci , H. P. Hratchian , J. V. Ortiz , A. F. Izmaylov , J. L. Sonnenberg , F. D. Williams , F. Lipparini , F. Egidi , J. Goings , B. Peng , A. Petrone , T. Henderson , D. Ranasinghe , V. G. Zakrzewski , et al., Gaussian 16, Revision C.01 , Gaussian, Inc., Wallingford CT 2016.

[24] M. Chen , Y. Liang , T. Dong , W. Liang , Y. Liu , Y. Zhang , X. Huang , L. Kong , Z.‐X. Wang , B. Peng , Angew. Chem. Int. Ed. 2021, 60, 2339. 10.1002/anie.202010740 33017503

[25] J. L. G.ía Ruano , L. Marzo , V. Marcos , C. Alvarado , J. Alemán , Chem.—Eur. J 2012, 18, 9775. 22847833 10.1002/chem.201201607

[26] M. S. Robinson , M. L. Polak , V. M. Bierbaum , C. H. DePuy , W. C. Lineberger , J. Am. Chem. Soc. 1995, 117, 6766.

[27] W. S. Matthews , J. E. Bares , J. E. Bartmess , F. G. Bordwell , F. J. Cornforth , G. E. Drucker , Z. Margolin , R. J. McCallum , G. J. McCollum , N. R. Vanier , J. Am. Chem. Soc. 1975, 97, 7006.

[28] R. Roy , S. Saha , RSC Adv. 2018, 8, 31129. 35548716 10.1039/c8ra04481c PMC9085608

[29] While **TS‐*S* _a_ *R* _a_‐4** is higher in energy than the other **TS‐4**, and is therefore not actively discussed, wish to make a note on the computed imaginary frequency: For **TS‐*S* _a_ *R* _a_‐4**, the imaginary frequency is unusually small (−9.7 cm^−1^); however, several numerical validations confirm the reliability of this transition state. These include: (1) the IRC connects the appropriate reactant and product complexes, albeit with limited steps; (2) a relaxed potential energy surface scan from the reactant complex shows a clear local maximum near the optimized geometry (ΔG = 7.8 kcal mol^−1^, at, −30.2 cm^−1^); and (3) reoptimization at a higher level of theory yields a structurally similar TS with a more well‐defined imaginary frequency (ΔG = 9.4 kcal mol^−^ ^1^, −610.1 cm^−1^). As seen, the energies of these geometries are similar to **TS‐*S* _a_ *R* _a_‐4**, supporting the physical relevance and energetic validity of the reported transition state geometry.

[30] D. J. Pasto , W. Kong , J. Org. Chem. 1989, 54, 4028.

[31] D. J. Pasto , W. Kong , J. Org. Chem. 1988, 53, 4807.

[32] E. Soriano , I. Fernández , Chem. Soc. Rev. 2014, 43, 3041. 24553788 10.1039/c3cs60457h

[33] B. Alcaide , C. Aragoncillo , P. Almendros , in Compr. Org. Synth, (Ed: P. Knochel ), Elsevier, Amsterdam 2014, 66.

[34] N. Graulich , WIREs Comput. Mol. Sci. 2011, 1, 172.

[35] Ş. Gül , F. Schoenebeck , V. Aviyente , K. N. Houk , J. Org. Chem. 2010, 75, 2115. 20166688 10.1021/jo100033d PMC2837782

[36] K. N. Houk , B. R. Beno , M. Nendel , K. Black , H. Y. Yoo , S. Wilsey , J. K. Lee , J. Mol. Struct. THEOCHEM. 1997, 398‐399, 169.

[37] C. Y. Legault , 2009.

