# Supplementary material for: A Base‐Mediated Rearrangement of the Benzylic 1,5‐Hexadipyridynyl Moiety
Source: Chempluschem. 2025 Aug 4;90(9):e202500252. doi: 10.1002/cplu.202500252 (PMC12435124; doi:10.1002/cplu.202500252)
Supplement: Supplementary file 1 — Supplementary Material [file CPLU-90-e202500252-s001.pdf]

Supporting Information

A Base-Mediated Rearrangement of the Benzylic 1,5-Hexadipyridynyl Moiety

Wouter A. Remmerswaal, Thai-Tony Nguyen, Han Zijan, Fedor Krasovski Slobodian, Ruisheng Xiong, Vadim Kessler, Zhijian Xu, Weiliang Zhu, Mate Erdelyi

Table of Contents

Supplementary organic synthesis methods....S2
General experimental procedures....S2
Sample preparation for VT-NMR experiments with **rac/meso-7**....S2
Sample preparation for VT-NMR experiments with **rac-1** and **meso-1**....S2
Preparation of rac and **meso-1**....S3
Experimental Supplementary Figure....S6
NMR spectra of new and selected compounds....S10
Supplementary X-ray Methods....S21
Methods for recording the crystal structure of ***EZ*-3**....S21
Supplementary X-ray Tables and Figures....S22
Supplementary Computational Methods....S28
Computational Details....S28
Conformational Sampling of Stationary Points....S28
Supplementary Computational Tables....S29
References....S45

Supplementary organic synthesis methods

General experimental procedures

All chemicals (Acros, Fluka, Merck, and Sigma-Aldrich) were used as received unless stated otherwise. TLC-analysis was conducted on TLC Silica gel 60 (Kieselgel 60 F_{254}, Supelco) with UV detection by (254 nm). Flash column chromatography was performed on silica gel 60 Å (0.04 – 0.063 mm, VWR), using a Biotage® Isolera™ One FCC system with Luknova SuperSep™ column cartridges. Purification with preparative RP-HPLC was performed on a VWR LaPrep P110 with single wavelength detection, using a Kromasil C8 column (10 µm, 100 Å, ø 39 mm, L 250 mm) and gradients of CH_3CN/H_2O with/without 0.1% formic acid as mobile phase at 10/15 mL min^{-1} flow rate. High-resolution accurate mass measurements were performed by ESIMS using an LTQ-Velos Pro Orbitrap mass analyzer (Thermo Fisher Scientific, Waltham, MA, USA) equipped with an Agilent 1100 autosampler (Agilent, SantaClara, CA, USA) with a bioZen Peptide XB-C18 column (100 x 2.1 mm, 1.7 µm). Gradient used was from 5-95% CH_3CN (with 0.01% formic acid) in H_2O (with 0.01% formic acid) at 0.8 mL/min. MS was scanned from 100 to 2500 Da with 1 scan/s. Each mass spectrum was obtained in the positive-ion mode, and the obtained data were processed using MassLynx V4.1 software. LC-MS were performed by ESIMS using an LC/MSD-iQ mass analyzer (Agilent, SantaClara, CA, USA) equipped with an Agilent 1260 Infinity II autosampler (Agilent, SantaClara, CA, USA) with a poroshell 120 EC-C18 column (50 x 2.1 mm, 2.7 µm). Gradient used was from 5-95% CH_3CN (with 0.01% formic acid) in H_2O (with 0.01% formic acid) at 0.5 mL/min. MS was scanned from 100 to 1 450 Da with 1 scan/s. ^{1}H and $^{13}C\{^{1}H\}$ NMR spectra were recorded on a Varian Unity 400, Bruker Avance NEO 500 or a Bruker Avance NEO 600 spectrometer. Chemical shifts (δ) are given in ppm relative to the residual signal of the deuterated solvent. Coupling constants (*J*) are given in Hz. To get better resolution of signals with small coupling constants or overlapping signals a gaussian window function (LB ± -1 and GB ± 0.5) was used on the ^{1}H NMR spectrum. All given ^{13}C spectra are proton decoupled. NMR peak assignment was made using COSY, HSQC, $^{1}H,^{13}C$ HMBC and $^{1}H,^{15}N$ HMBC experiments. Data processing was carried out using MestReNova version 14.1.2 (Mestrelab Research S.L.).

Sample preparation for VT-NMR experiments with rac/meso-7

A solution of **2:1 rac/meso-7** (5 mg, 0.022 mmol, 1.0 equiv.), triethylamine (0.03 ml, 0.217 mmol, 10.0 equiv.), 2,6-diiodopyridine (15.84 mg, 0.048 mmol, 2.2 equiv.) and DMF-*d7* (0.2 ml) was prepared. The mixture was degassed with argon for 10 min, followed by addition of bis(triphenylphosphine) palladium(II) chloride (1.52 mg, 0.002 mmol, 0.1 equiv.) and copper (I) iodide (0.207 mg, 0.001 mmol, 0.05 equiv). The mixture was degassed further for 5 min, and subsequently transferred to an NMR tube. NMR spectra were then recorded in the range of 20 °C to 70 °C, measuring each spectra with 10 minute and 10 °C intervals.

Sample preparation for VT-NMR experiments with rac-1 and meso-1

A solution of **rac-1 or meso-1** (0.75 mg, 0.002 mmol, 1.0 equiv.) in DMF-*d7* (0.2 ml) was prepared. The mixture was degassed with argon for 10 min, followed by addition of one or more of the following reagents: previously degassed triethylamine (0.03 ml, 0.217 mmol, 10.0 equiv.), bis(triphenylphosphine) palladium(II) chloride (1.52 mg, 0.002 mmol, 0.1 equiv.), copper (I) iodide (0.207 mg, 0.001 mmol, 0.05 equiv.), TEMPO (1.22 mg, 0.008 mmol, 4 equiv.). NMR spectra were then either recorded in the range of 20 °C to 70 °C, measuring each spectra with 10 minute and 10 °C intervals, or the sample was followed over time at the stable temperature of 30 °C.

Preparation of rac and meso-1

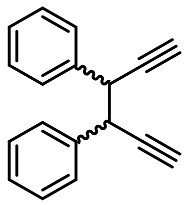

Hexa-1,5-diyne-3,4- diyldibenzene (7)[1,2]

Commercially available 1-Phenyl-3-(trimethylsilyl)prop-2-yn-1-ol **4** (2.0 mL, 9.12 mmol. 1.0 equiv.) was dissolved in Et_2O (10 ml) an cooled to 0 °C. While stirring, PBr3 (1.23 ml, 13.69 mmol, 1.5 equiv.) was added dropwise and stirred at 0 °C for 1 h. Subsequently, the reaction was allowed to heat up to room temperature and stirred for another 1 h. The reaction was quenched by the dropwise addition of aq. NaOH (2M) until pH of 8-9 was reached. The reaction mixture was diluted with H_2O and extracted with DCM. The organic layers were combined, washed with brine, dried over $MgSO_4$ and concentrated *in vacuo*, yielding (3-bromo-3-phenylprop-1-yn-1-yl)trimethylsilane **5** as yellow oil. The oil was dissolved in dry THF (6.6 ml) under N_2 atmosphere. Tris(acetylacetonato)-iron(III) (64.44 mg, 0.18 mmol, 0.02 equiv.) and Mg turnings (443.5 mg, 18.25 mmol, 2.0 equiv.) were added and the reaction mixture was stirred at room temperature overnight. The solution turned from red to black in ca. 30 min. The reaction was cooled down to 0 °C and while stirring, 1M aq. HCl (4 ml) was added dropwise. The reaction mixture was diluted with H_2O and extracted with Et_2O. The organic layers were combined, washed with brine, dried over $MgSO_4$ and concentrated *in vacuo*, yielding the crude (3,4-diphenylhexa-1,5-diyne-1,6-diyl)bis(trimethylsilane) **6** as a brown oil (1.71 g, 4.56 mmol). KF (1.57 g, 29.33 mmol, 7.0 equiv.) was added to a solution of the crude in MeOH (37 ml). The mixture was stirred overnight at 55 °C. The reaction mixture was then diluted with H_2O and extracted with Et_2O. The organic layers were combined, washed with brine, dried over $MgSO_4$ and concentrated *in vacuo*. The obtained brown oil was diluted with MeOH, effecting a white solid to precipitate. Filtration of the white solid, provided **meso-7** (148.6 mg, 0.65 mmol, 7 % over three steps). Concentration of the filtrate *in vacuo*, followed by flash column chromatography (100:0 → 98:2, petroleum ether:EtOAc v:v) yielded the title compound (0.613 g, quant) as a brown oil (570.4 mg, 2.48 mmol, 27 % over three steps). Analytical data for **meso-7:** TLC: R_f 0.42, (petroleum ether); ^{1}H NMR (500 MHz, CD_2Cl_2) δ 7.30 – 7.20 (m, 10H, CH_{arom}), 4.04 (s, 2H, CH-propargylic), 2.35 (s, 2H, CH-alkyne); ^{13}C NMR (151 MHz, $CDCl_3$) δ 138.26 ($C_{q\text{-}arom}$), 129.09 ($CH_{arom\text{-}ortho}$), 128.35 ($CH_{arom\text{-}meta}$), 127.83 ($CH_{arom\text{-}prara}$), 83.71 ($C_{alkyn\text{-}phenyl}$), 73.45 (CH_{alkyne}), 45.23 ($CH_{propargylic}$). Analytical data for **meso/rac-7** mixture**:** TLC: R_f 0.42, (petroleum ether); ^{1}H NMR (400 MHz, CD_2Cl_2) δ 7.40 – 7.19 (m, 10H, CH_{arom}), 4.03 (s, 1H, $CH_{propargylic}$), 2.41 (s, 1H, CH_{alkyne}).; ^{13}C NMR (101 MHz, $CDCl_3$) δ 138.30 ($C_{q\text{-}arom}$), 128.78 ($CH_{arom\text{-}ortho}$), 128.54 ($CH_{arom\text{-}meta}$), 127.82 ($CH_{arom\text{-}para}$), 83.75 ($C_{alkyn\text{-}phenyl}$), 73.65 (CH_{alkyne}), 45.73 ($CH_{propargylic}$).

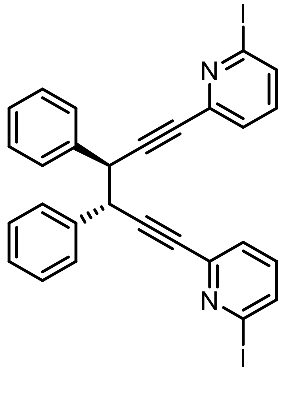

6,6'-(3,4-Diphenylhexa-1,5-diyne-1,6-diyl)bis(2-iodopyridine) (rac-1)

A solution of **2:1 rac/meso-7** (50 mg, 0.22 mmol, 1.0 equiv.), triethylamine (0.3 ml, 2.17 mmol, 10.0 equiv.), 2,6-diiodopyridine (158.04 mg, 0.48 mmol, 2.2 equiv.) and dry DMF (1 ml) was prepared. The mixture was degassed with argon for 25 min, followed by addition of bis(triphenylphosphine) palladium(II) chloride (15.24 mg, 0.02 mmol, 0.1 equiv.) and copper (I) iodide (2.07 mg, 0.01 mmol, 0.05 equiv). The mixture was degassed further for 5 min, and subsequently heated to 40 °C for 30 min by microwave. The solution was diluted with deionized-H_2O and the aqueous phase was extracted with EtOAc (3x). The organic layers were combined, washed with brine, dried over $MgSO_4$ and concentrated *in vacuo*. After purification through flash column chromatography (99:1 → 90:10, petroleum ether:EtOAc v:v) and preparative HPLC (ACN/H_2O 70:30 to 85:15) the title compound was obtained as a yellow oil (10.2 mg, 7.4%). TLC: R_f 0.40, (petroleum ether:EtOAc, 85:15, v:v); ^{1}H NMR (600 MHz, DMF-d_7) δ 7.67 (dd, J = 7.9, 1.0 Hz, 2H, $CH_{arom-meta-pyridine}$), 7.50 (dd, J = 7.7, 1.0 Hz, 2H, $CH_{arom-ortho-pyridine}$), 7.40 – 7.26 (m, 12H, CH_{arom}), 4.33 (s, 2H, $CH_{propargylic}$); ^{13}C NMR (151 MHz, $CDCl_3$) δ 144.43 ($Cq_{pyridine}$), 138.32 (*C*q-pyridine), 137.98 ($CH_{arom-meta-pyridine}$), 134.61 ($CH_{arom-para-pyridine}$), 128.85 ($CH_{arom-phenyl}$), 128.79 ($CH_{arom-phenyl}$), 128.11 ($CH_{arom-phenyl}$), 127.19 ($CH_{arom-ortho-pyridine}$), 117.56 ($C_{q-arom-phenyl}$), 90.90 ($C_{alkyne-propargylic}$), 84.50 ($C_{alkyne-pyridine}$), 46.77 ($CH_{propargylic}$); HRMS (ESI) m/z: $[M + Na]^+$ Calcd for $C_{28}H_{18}I_2N_2Na^+$ 658.9452; Found 658.9522.

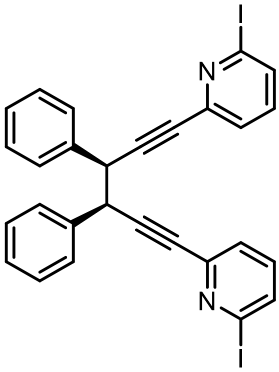

6,6'-(3,4-Diphenylhexa-1,5-diyne-1,6-diyl)bis(2-iodopyridine) (meso-1)

Meso-1 was prepared from **meso-6** through identical reaction conditions as when starting from a 2:1 mixture of **rac/meso-6**. The reaction mixture was diluted with aq. sat. NH_4Cl, and the aqueous phase was extracted with EtOAc (3 ×). The combined organic phases were washed with brine, separated and organic phase was dried over $MgSO_4$. After filtration, the solvent was removed under reduced pressure. After purification through flash column chromatography (100:0 → 90:10, petroleum ether:EtOAc v:v) the title compound was obtained as a yellow solid (10.3 mg, 7.5%). R_f 0.40, (petroleum ether:EtOAc, 85:15, v:v). ^{1}H NMR (601 MHz, CD_2Cl_2) δ 7.66 (dd, J = 7.8, 1.0 Hz, 2H, $CH_{arom-meta-pyridine}$), 7.36 – 7.27 (m, 14H, CH_{arom}), 4.39 (s, 2H, propargylic);^{13}C NMR (151 MHz, CD_2Cl_2) δ 144.32 ($Cq_{pyridine}$), 137.84 ($CH_{arom-pyridine}$), 134.59 ($CH_{arom-pyridine}$), 129.18 ($CH_{arom-phenyl}$), 128.62 ($CH_{arom-phenyl}$), 128.13 ($CH_{arom-para-phenyl}$), 127.00 ($CH_{arom-pyridine}$), 117.56 ($C_{q-arom-phenyl}$), 91.20 ($C_{alkyne-propargylic}$), 84.35 ($C_{alkyne-pyridine}$), 46.15 ($CH_{propargylic}$). HRMS (ESI) m/z: $[M + Na]^+$ Calcd for $C_{28}H_{18}I_2N_2Na^+$ 658.9452; Found 658.9417.

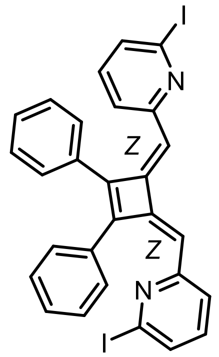

6,6'-((1Z,1'Z)-(3,4-Diphenylcyclobut-3-ene-1,2-diylidene)bis(methaneylylidene))bis-(2-iodopyridine) (*ZZ*-3)

EZ-3 was isolated through crystallization during the synthesis of **rac/meso-1.** ^{1}H NMR (500 MHz, DMF-d_7) δ 7.62 (d, *J* = 7.7 Hz, 2H, CH_{arom}), 7.40 – 7.33 (m, 6H, CH_{arom}), 7.27 (t, *J* = 7.7 Hz, 2H, CH_{arom}), 7.23 – 7.15 (m, 6H, CH_{arom}), 6.76 (s, 2H, $CH_{alkene\text{-}Z}$); ^{13}C NMR (126 MHz, DMF-d_7) δ 157.40 ($C_{q\text{-}arom}$), 156.79 ($C_{q\text{-}arom}$), 145.81 ($C_{q\text{-}arom}$), 138.73 (CH_{arom}), 133.71 ($C_{q\text{-}arom}$), 133.65 (CH_{arom}), 130.02 ($C_{q\text{-}arom}$), 129.54 (CH_{arom}), 129.27 (CH_{arom}), 124.94 (CH_{arom}), 118.51 (CH_{arom}), 110.74 ($CH_{alkene\text{-}Z}$). HRMS (ESI) m/z: [M + Na]$^+$ Calcd for $C_{28}H_{18}I_2N_2Na^+$ 658.9452; Found 658.9470.

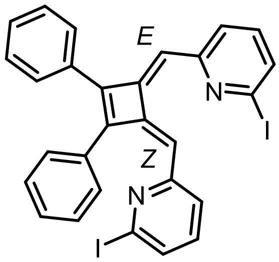

6,6'-((1Z,1'E)-(3,4-diphenylcyclobut-3-ene-1,2-diylidene)bis(methaneylylidene))-bis(2-iodopyridine) (*EZ*-3)

EZ-3 was isolated through crystallization during the synthesis of **rac/meso-1.** ^{1}H NMR (500 MHz, DMF-d_7) δ 8.14 (s, 1H, $CH_{alkene\text{-}Z}$), 7.75 (d, *J* = 7.6 Hz, 1H, $CH_{arom\text{-}pyridine}$), 7.65 (d, *J* = 7.6 Hz, 1H, $CH_{arom\text{-}pyridine}$), 7.62 – 7.55 (m, 4H, CH_{arom}), 7.51 – 7.45 (m, 3H, CH_{arom}), 7.40 – 7.35 (m, 2H, CH_{arom}), 7.35 – 7.28 (m, 3H, CH_{arom}), 7.17 (dd, *J* = 7.4, 1.6 Hz, 2H, CH_{arom}), 6.70 (s, 1H, $CH_{alkene\text{-}E}$); ^{13}C NMR (126 MHz, DMF-d_7) δ 158.09 (C_q), 158.05 (C_q), 155.37 (C_q), 148.70 (C_q), 145.87 (C_q), 140.14 (CH_{arom}), 138.67 (CH_{arom}), 134.54 ($C_{q\text{-}arom}$), 133.49 (CH_{arom}), 133.34 (CH_{arom}), 132.03 ($C_{q\text{-}arom}$), 130.47 (CH_{arom}), 129.95 (2x CH_{arom}), 129.73 (CH_{arom}), 129.22 (2x CH_{arom}), 129.04 (2x CH_{arom}), 128.98 (2x CH_{arom}), 128.85 ($C_{q\text{-}arom}$), 125.40 (CH_{arom}), 125.22 (CH_{arom}), 119.18 ($CH_{\text{-}alkene\text{-}E}$), 118.43 ($C_q$), 118.25 ($C_q$), 113.91 ($CH_{\text{-}alkene\text{-}E}$). HRMS (ESI) m/z: [M + Na]$^+$ Calcd for $C_{28}H_{18}I_2N_2Na^+$ 658.9452; Found 658.9490.

Experimental Supplementary Figure

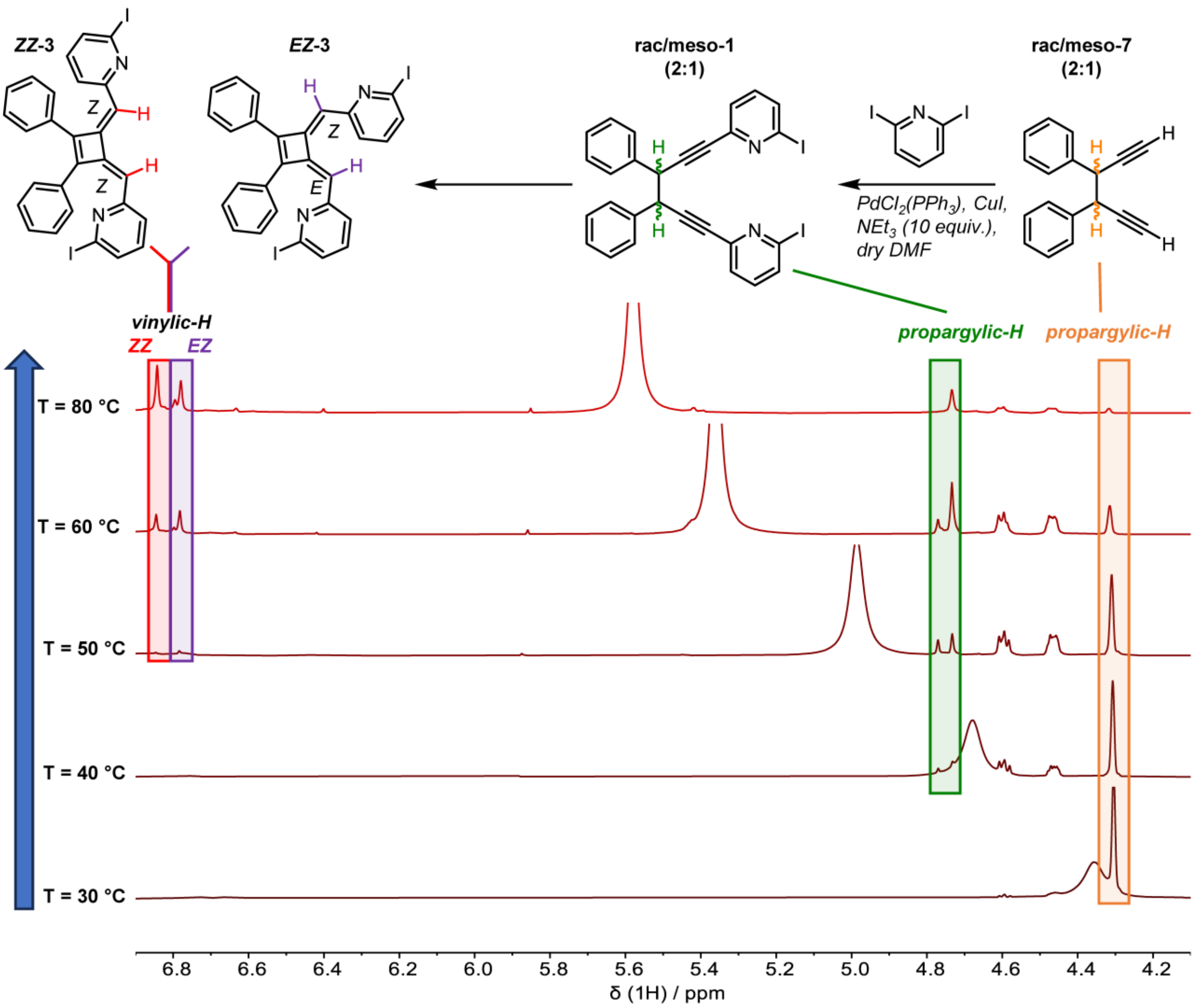

Supplementary Figure S1. VT-NMR of the Sonogashira reaction between between **7** and 2,6-diiodopyridine. Between each temperature step the sample was equilibrated for the temperature for 10 minutes. *Reagents and conditions*: **7,** $PdCl_2(PPh_3)$, CuI, NEt_3 (10 equiv.), DMF-d_7, 30 - 80 °C.

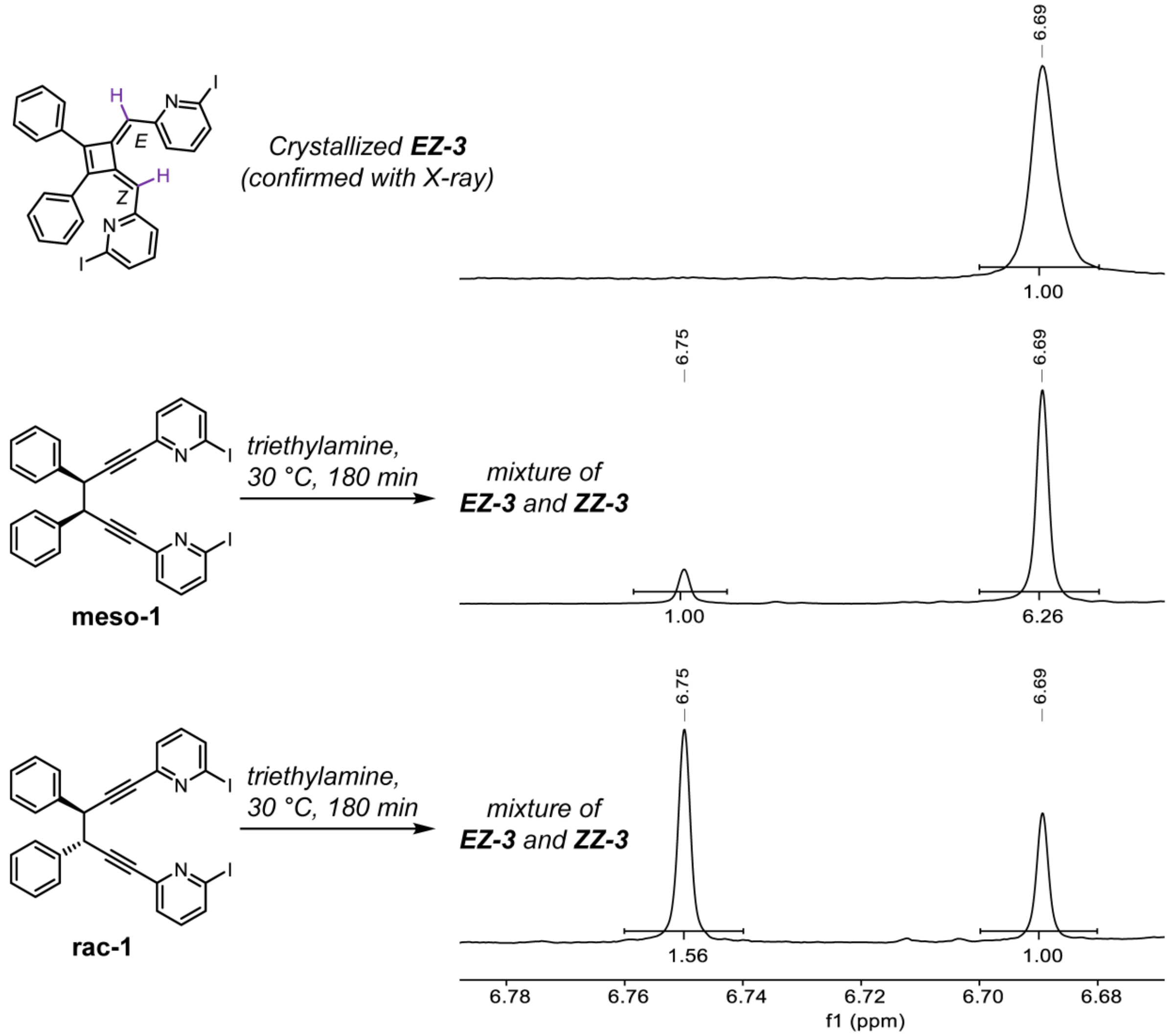

Supplementary Figure S2. Stacked NMR spectra of crystalized ***EZ*-3** (as confirmed with X-ray diffraction experiments), with the crude reaction mixtures of **meso-1** and **rac-1** when exposed to 50 equiv. of triethylamine for 180 minutes at 30 °C. All spectra were baseline corrected using the Bernstein Polynomial Fit method, with Polynomial-*N* = 3. Integrations of ***ZZ*-3** were performed with a window ranging from 6.76-6.74 ppm, and integration of ***EZ*-3** were performed with a window ranging from 6.70-6.68 ppm.

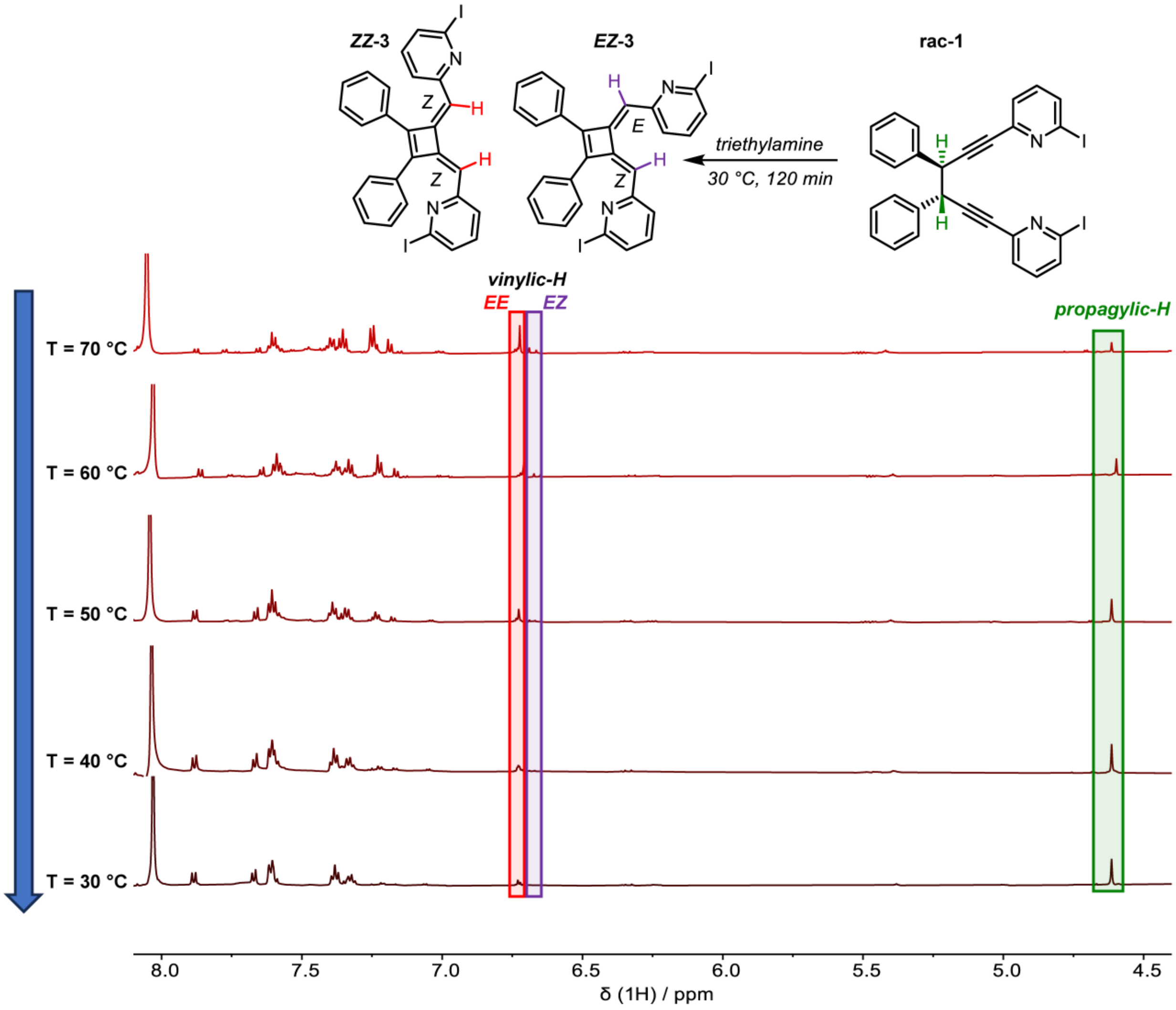

Supplementary Figure S3. VT-NMR of the Sonogashira reaction between between **7** and 2,6-diiodopyridine with radical scavenger present in the reaction mixture. Between each temperature step the sample was equilibrated for the temperature for 10 minutes. *Reagents and conditions*: **7,** $PdCl_2(PPh_3)$, CuI, NEt_3 (10 equiv.), TEMPO (4 equiv.), DMF-d_7, 30 - 70 °C.

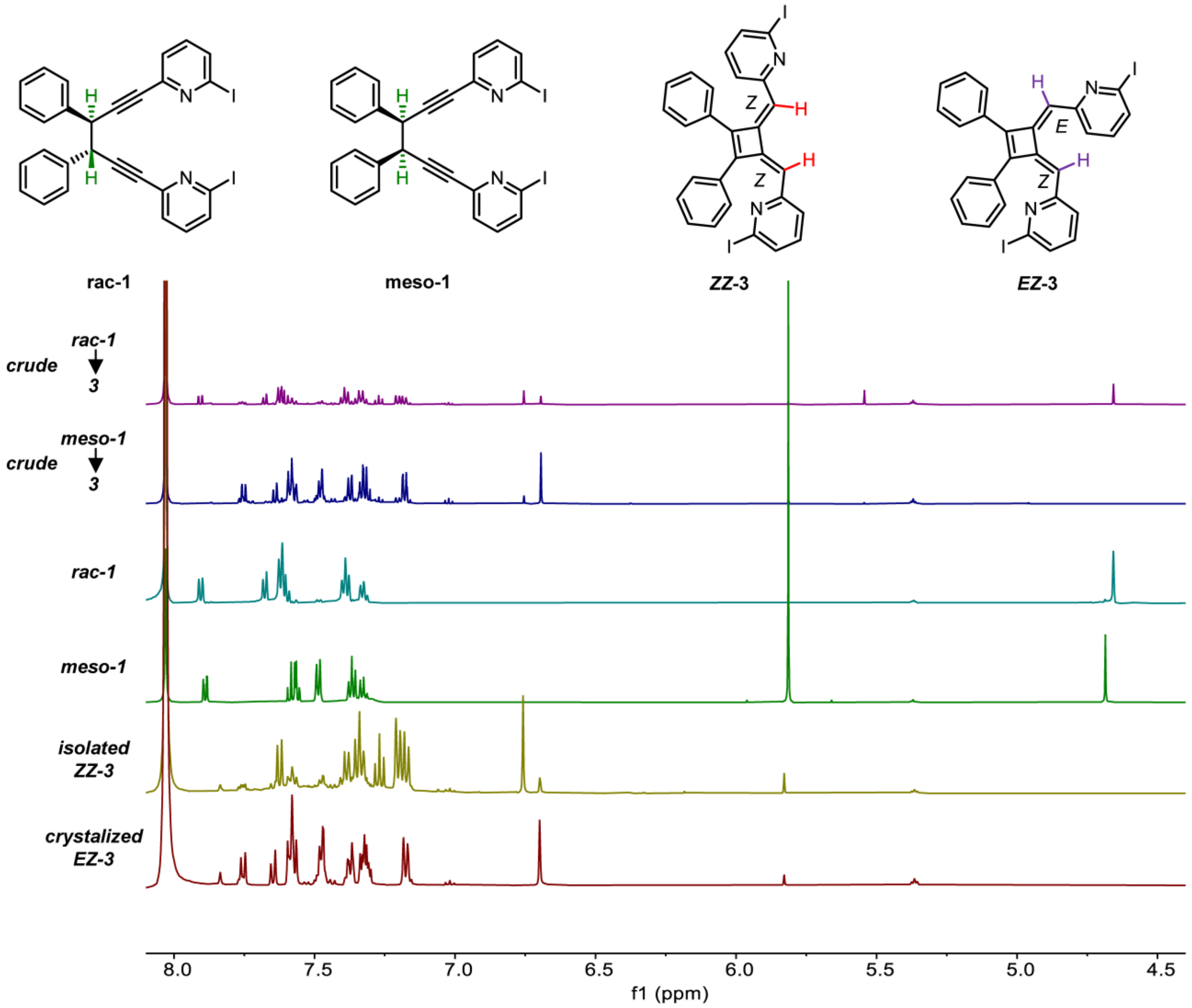

Supplementary Figure S4. NMR of the crude of **rac-1** and **meso-2** subjected to Sonogashira reaction conditions, with NMR spectra of isolated **rac-1**, **meso-2**, **EZ-3** and **ZZ-3** as references. *Reagents and condition*s: **1,** NEt_3 (10 equiv.), DMF-d_7, 30 °C, 180 minutes.

NMR spectra of new and selected compounds

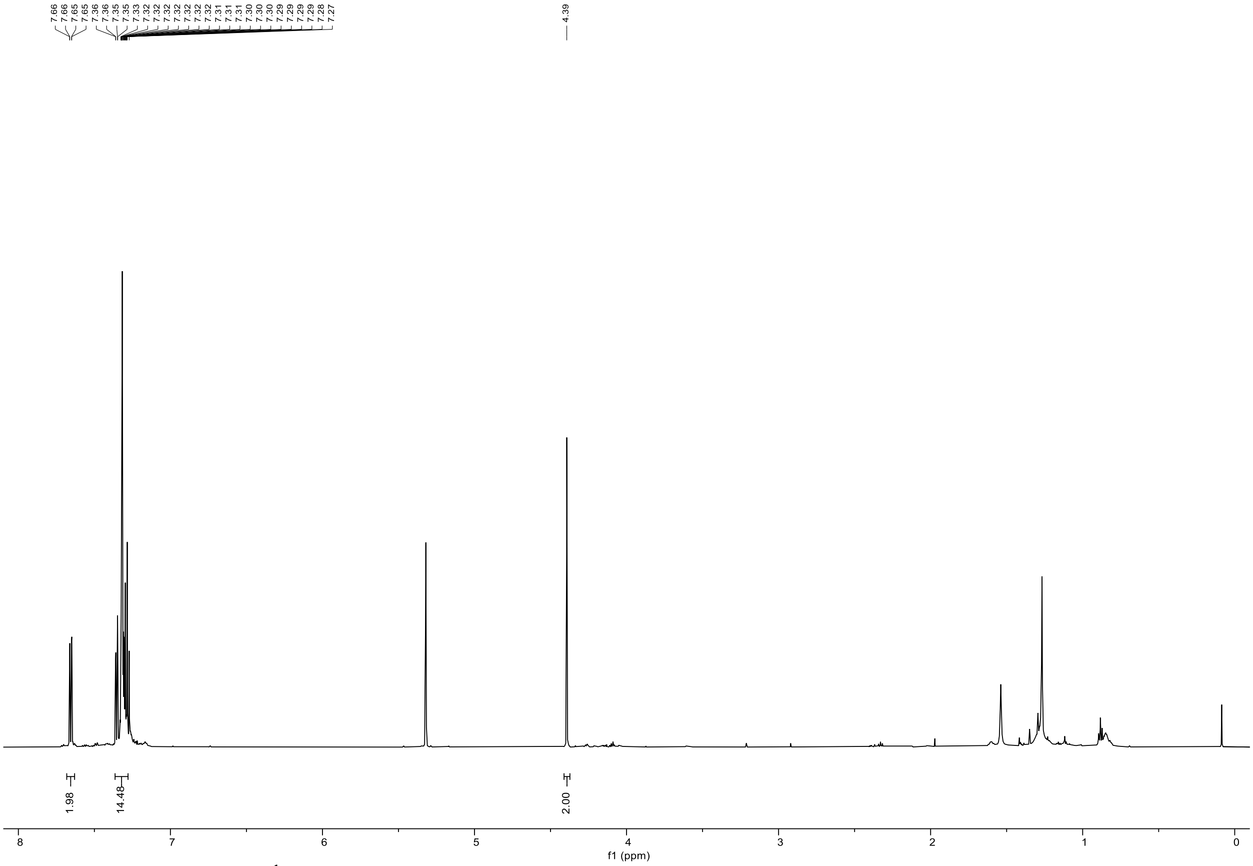

Supplementary Figure S5. ^{1}H NMR, 601 MHz, CD_2Cl_2 of compound **meso-1**

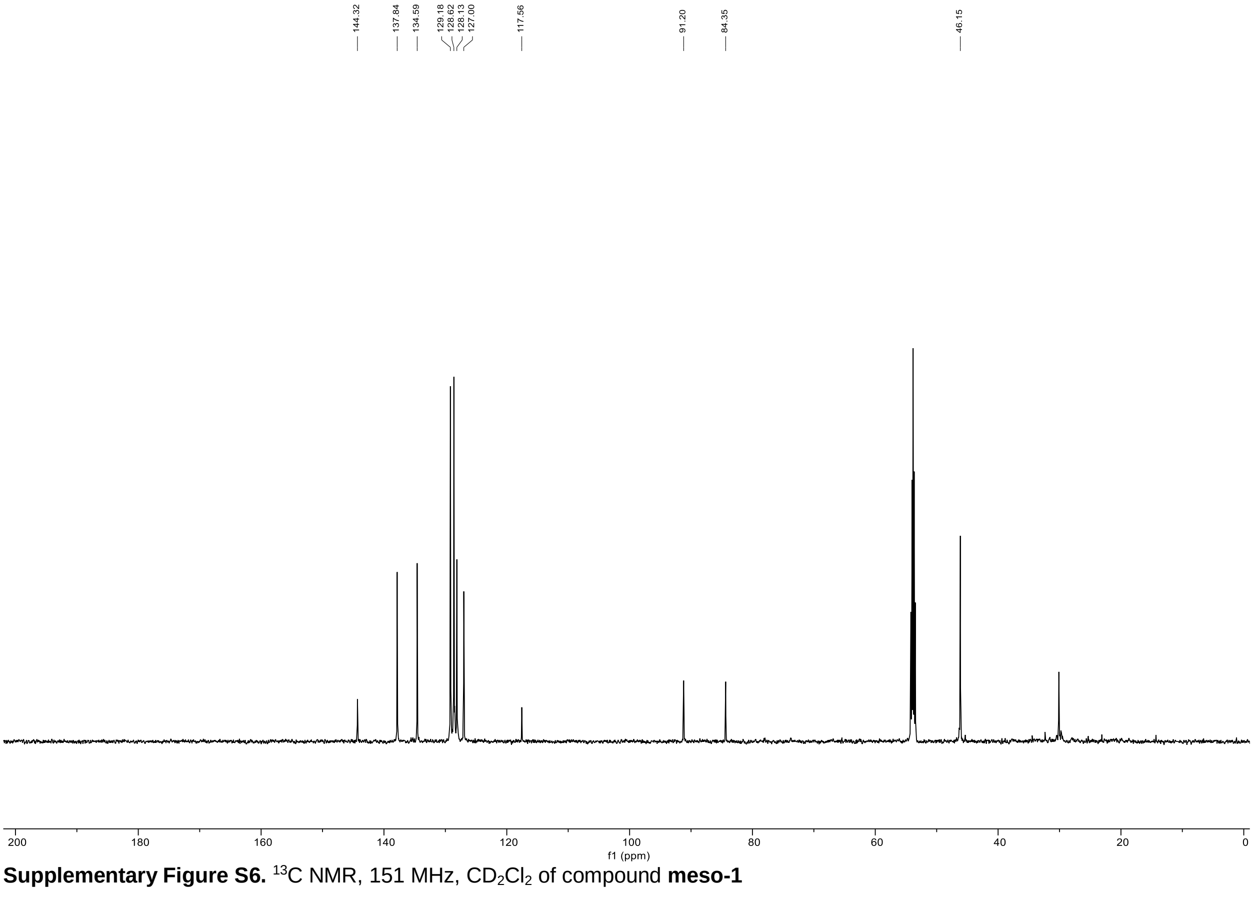

Supplementary Figure S6. ^{13}C NMR, 151 MHz, CD_2Cl_2 of compound **meso-1**

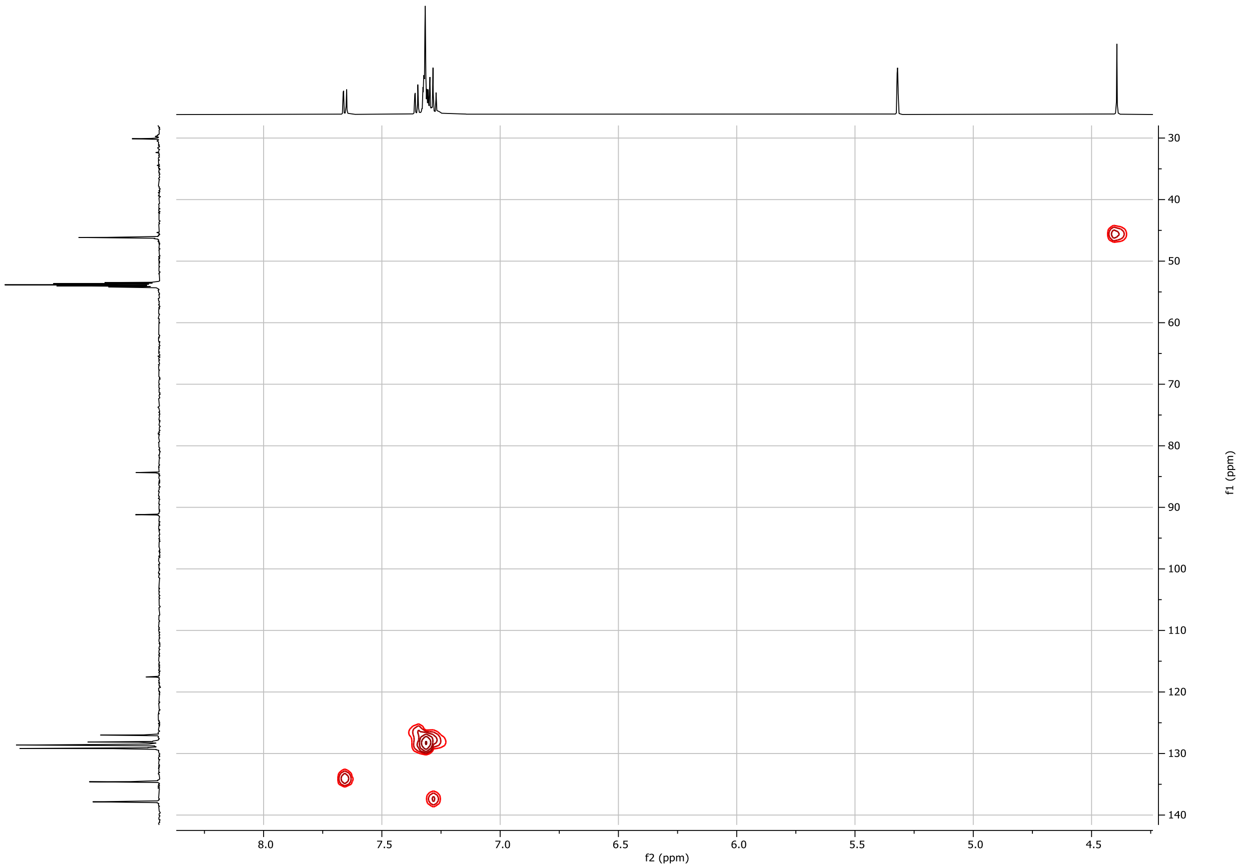

Supplementary Figure S7. HSQC NMR, CD_2Cl_2 of compound **meso-1**

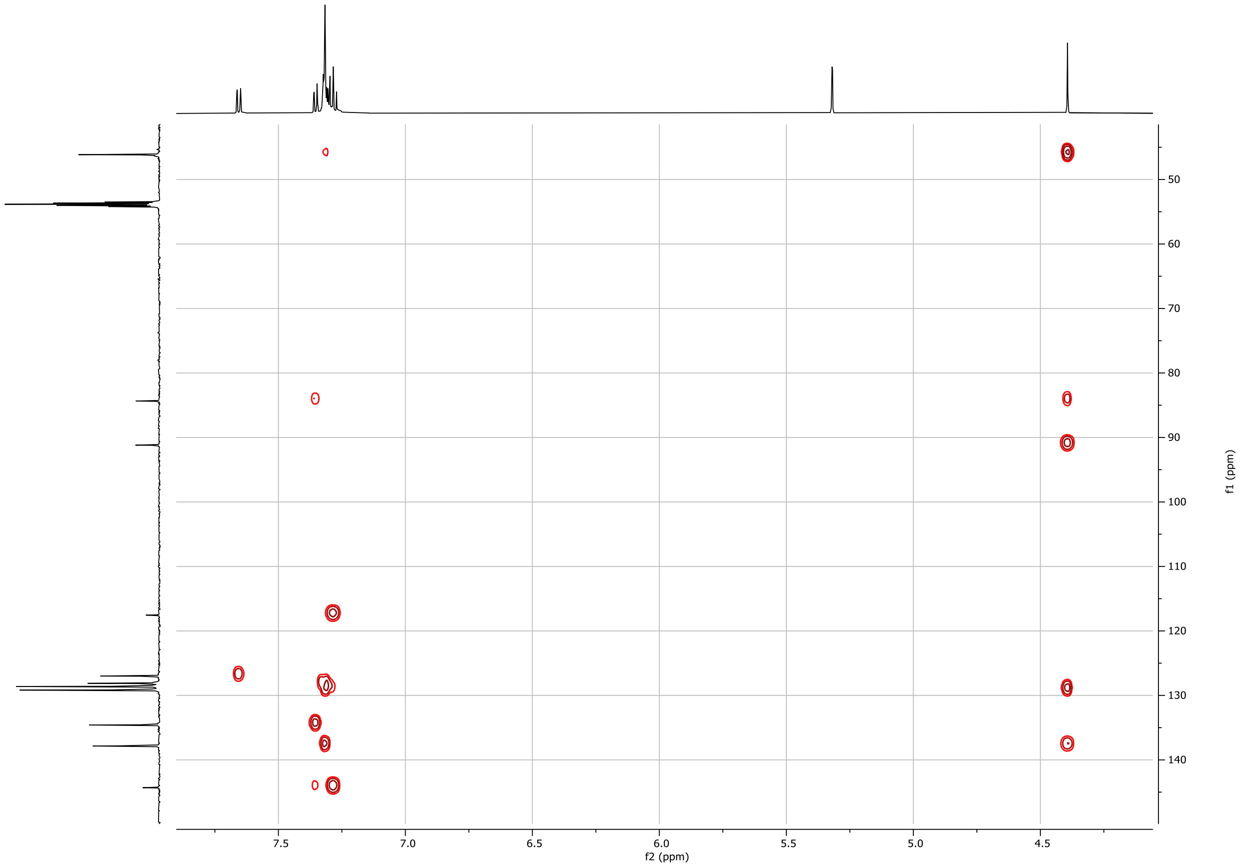

Supplementary Figure S8. HMBC NMR, CD_2Cl_2 of compound **meso-1**

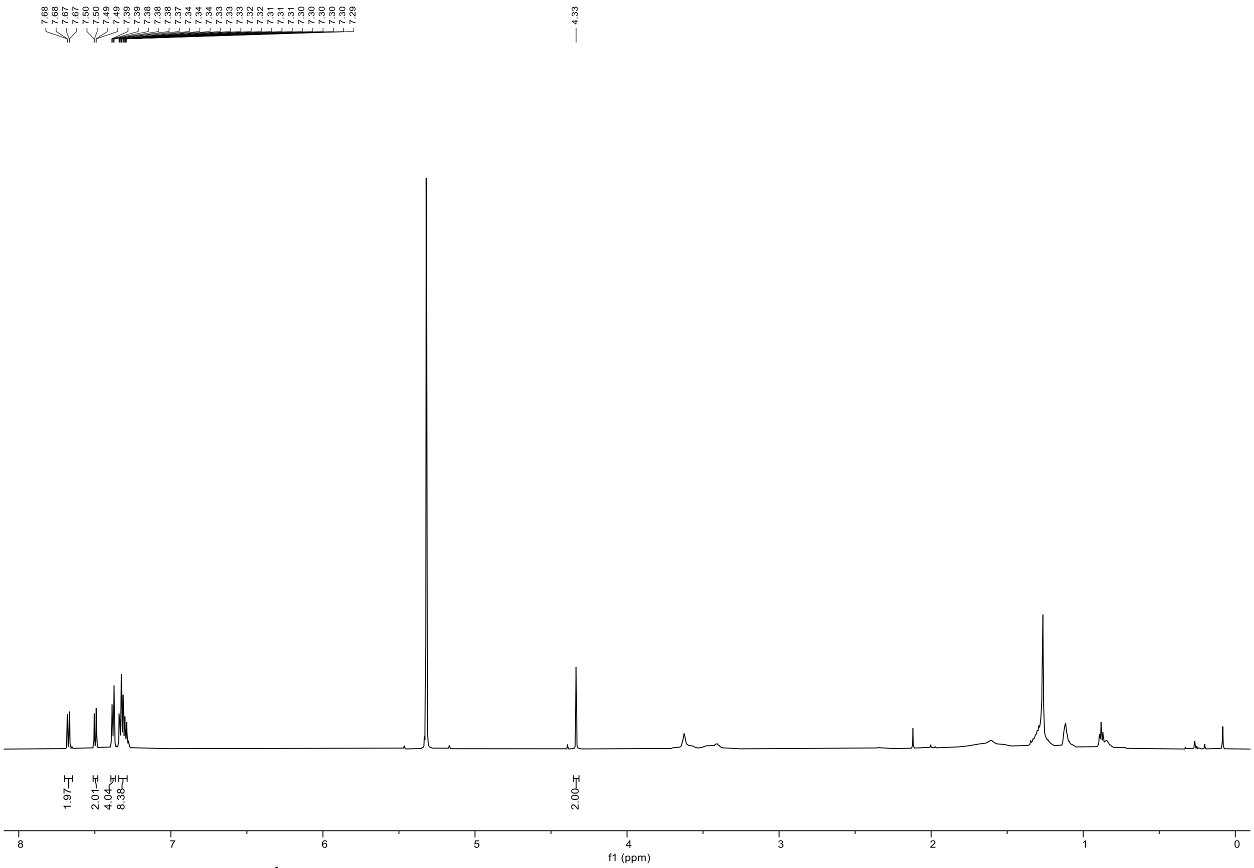

Supplementary Figure S9. ^{1}H NMR, 601 MHz, $CDCl_3$ of compound **rac-1**

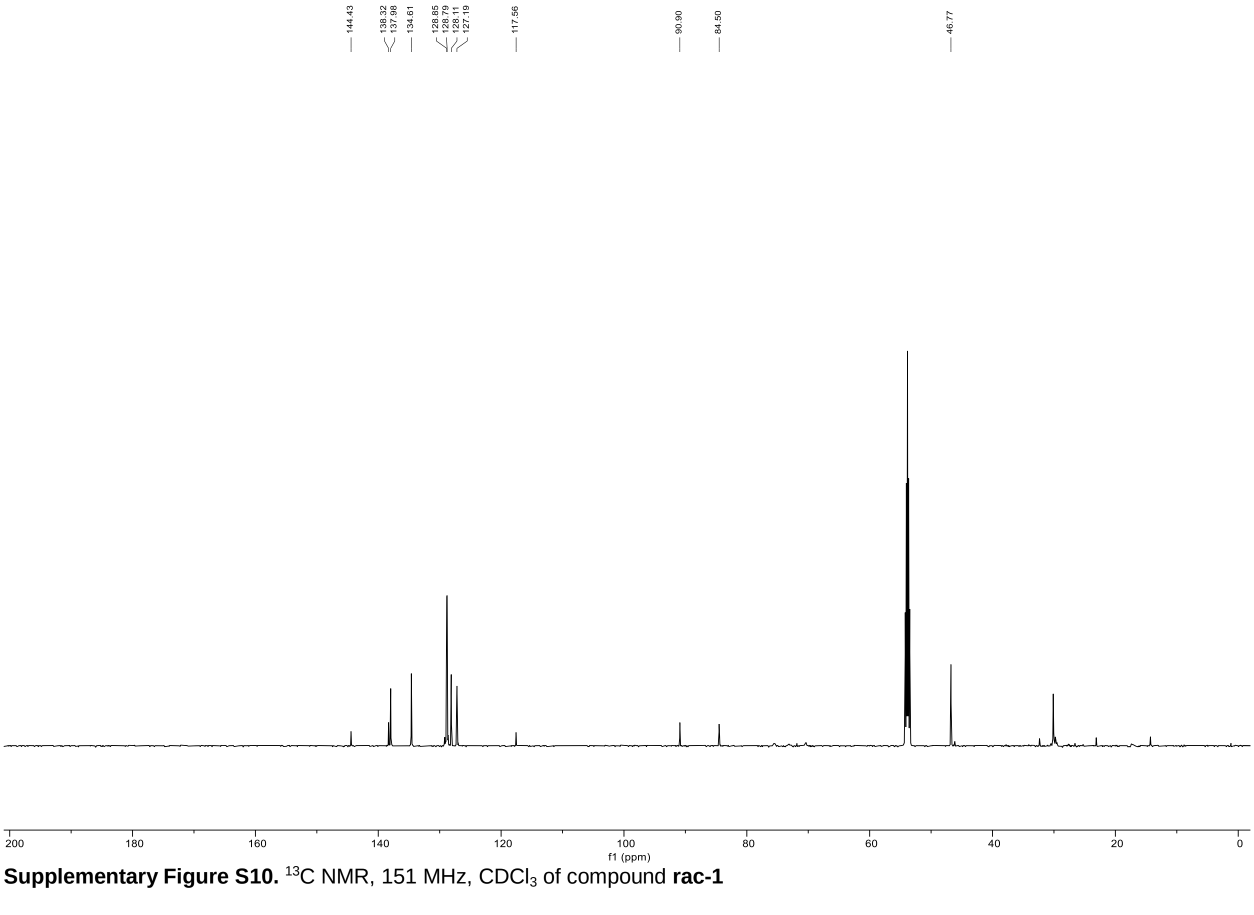

Supplementary Figure S10. ^{13}C NMR, 151 MHz, $CDCl_3$ of compound **rac-1**

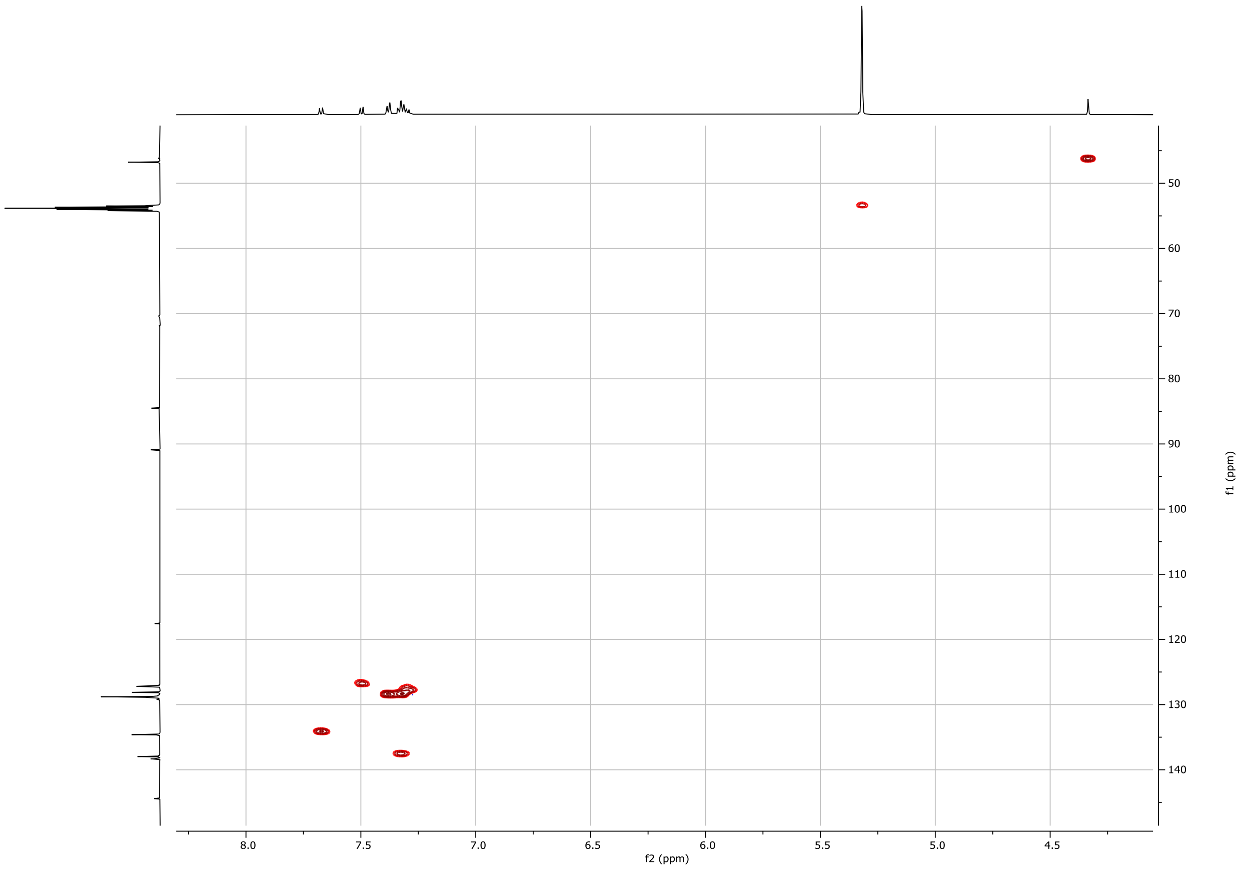

Supplementary Figure S11. HSQC NMR, $CDCl_3$ of compound **rac-1**

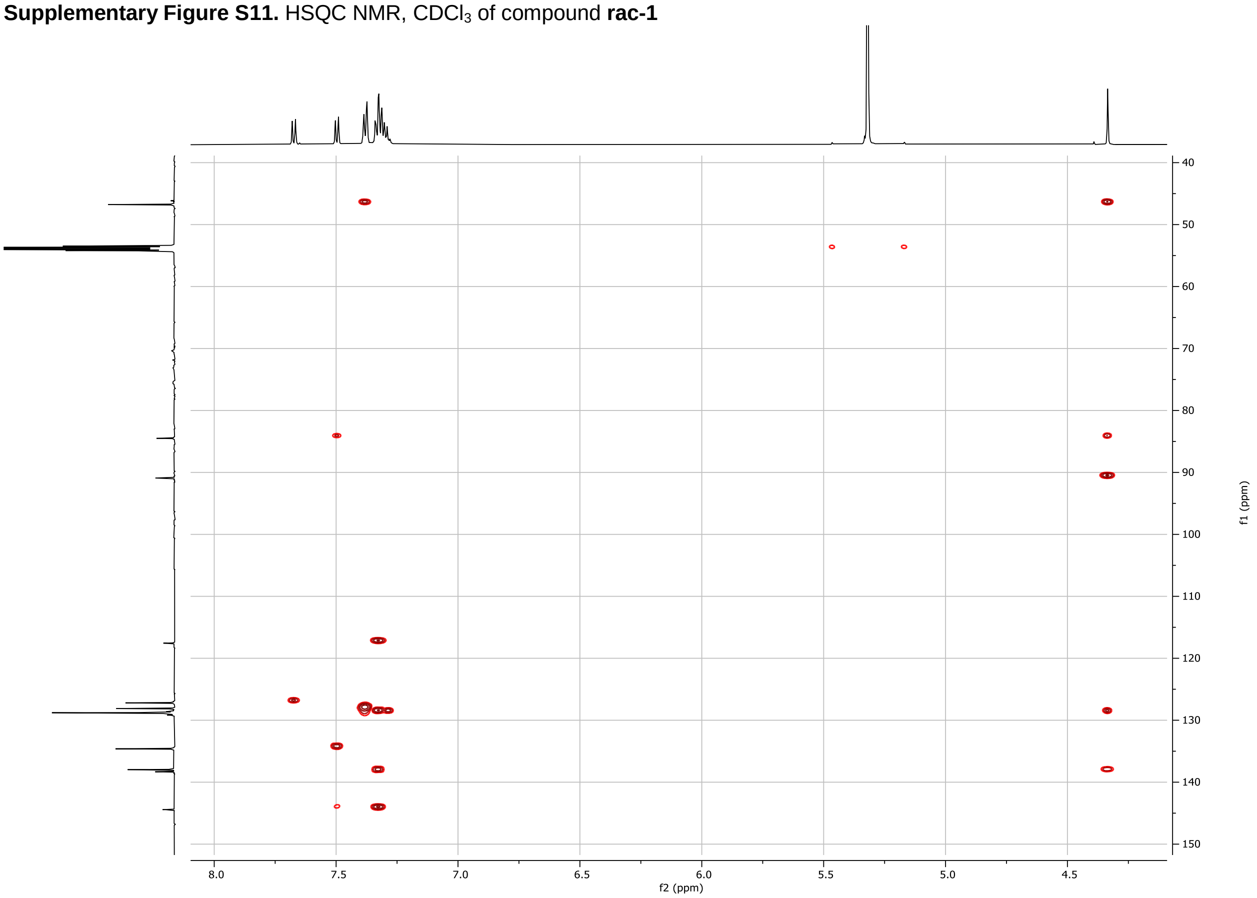

Supplementary Figure S12. HMBC NMR, $CDCl_3$ of compound **rac-1**

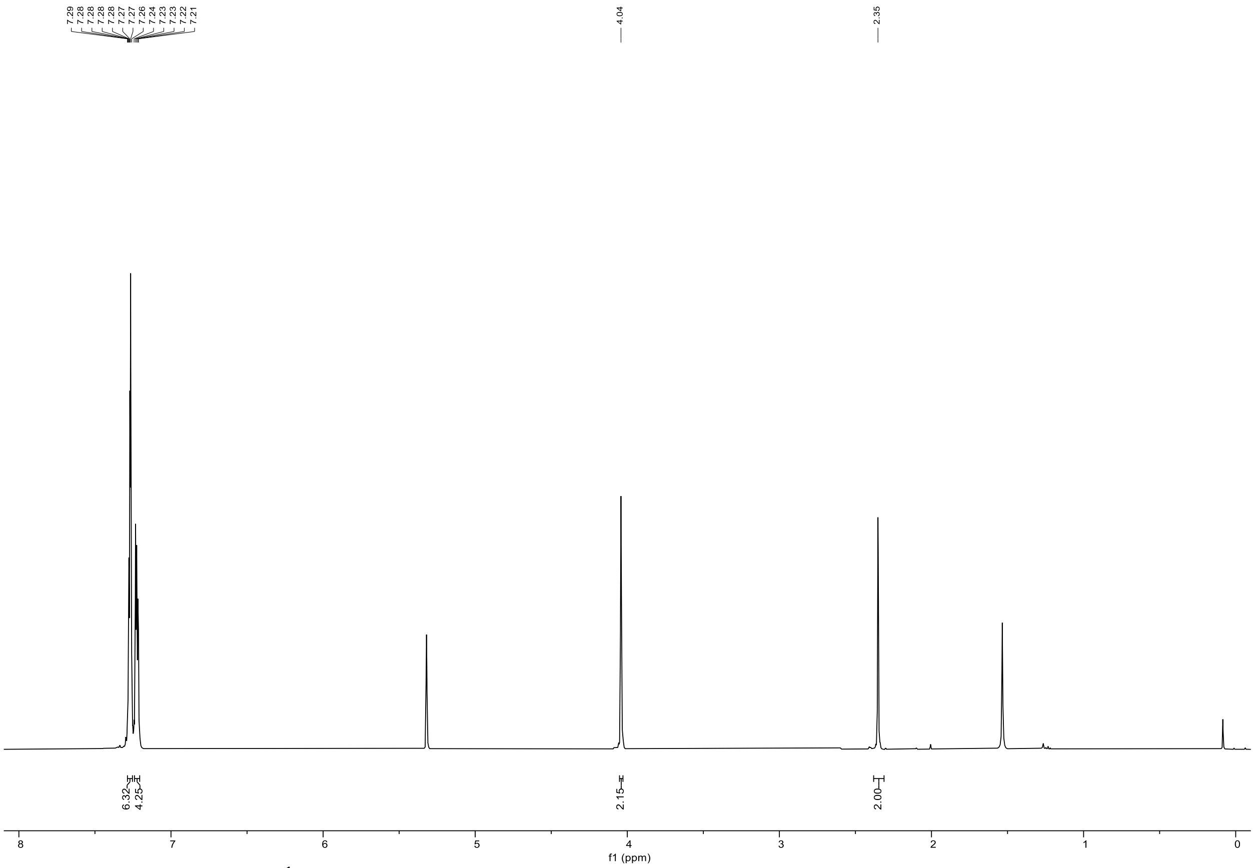

Supplementary Figure S13. ^{1}H NMR, 500 MHz, CD_2Cl_2 of compound **meso-7**

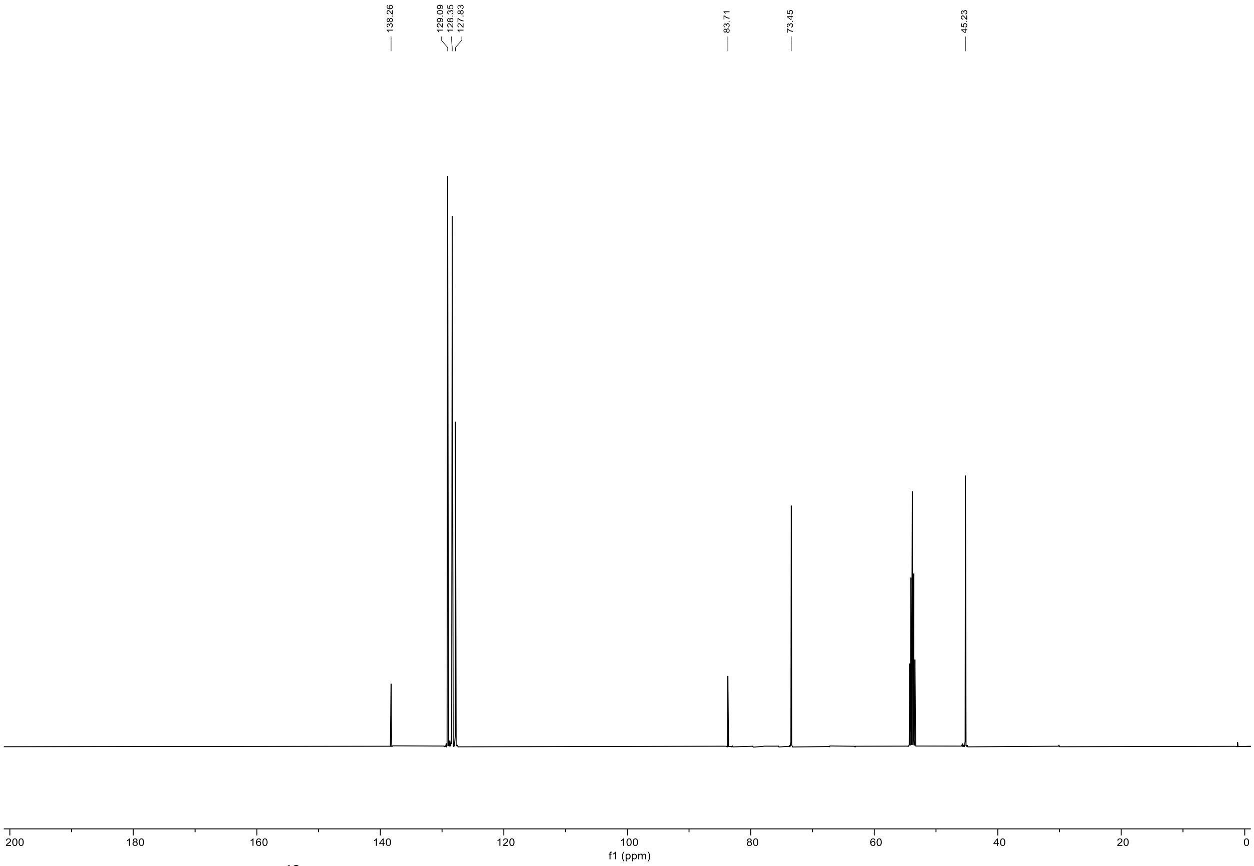

Supplementary Figure S14. ^{13}C NMR, 126 MHz, CD_2Cl_2 of compound **meso-7**

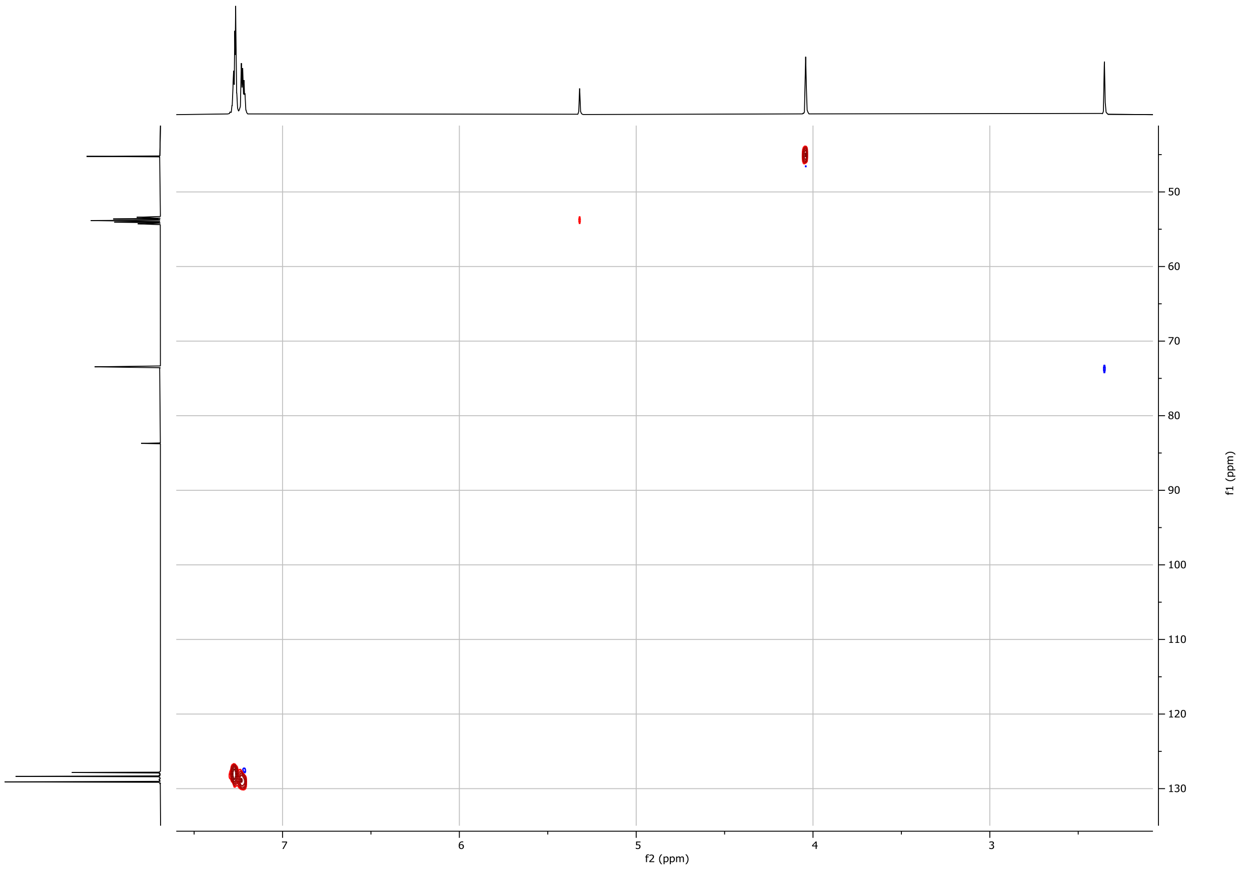

Supplementary Figure S15. HSQC NMR, CD_2Cl_2 of compound **meso-7**

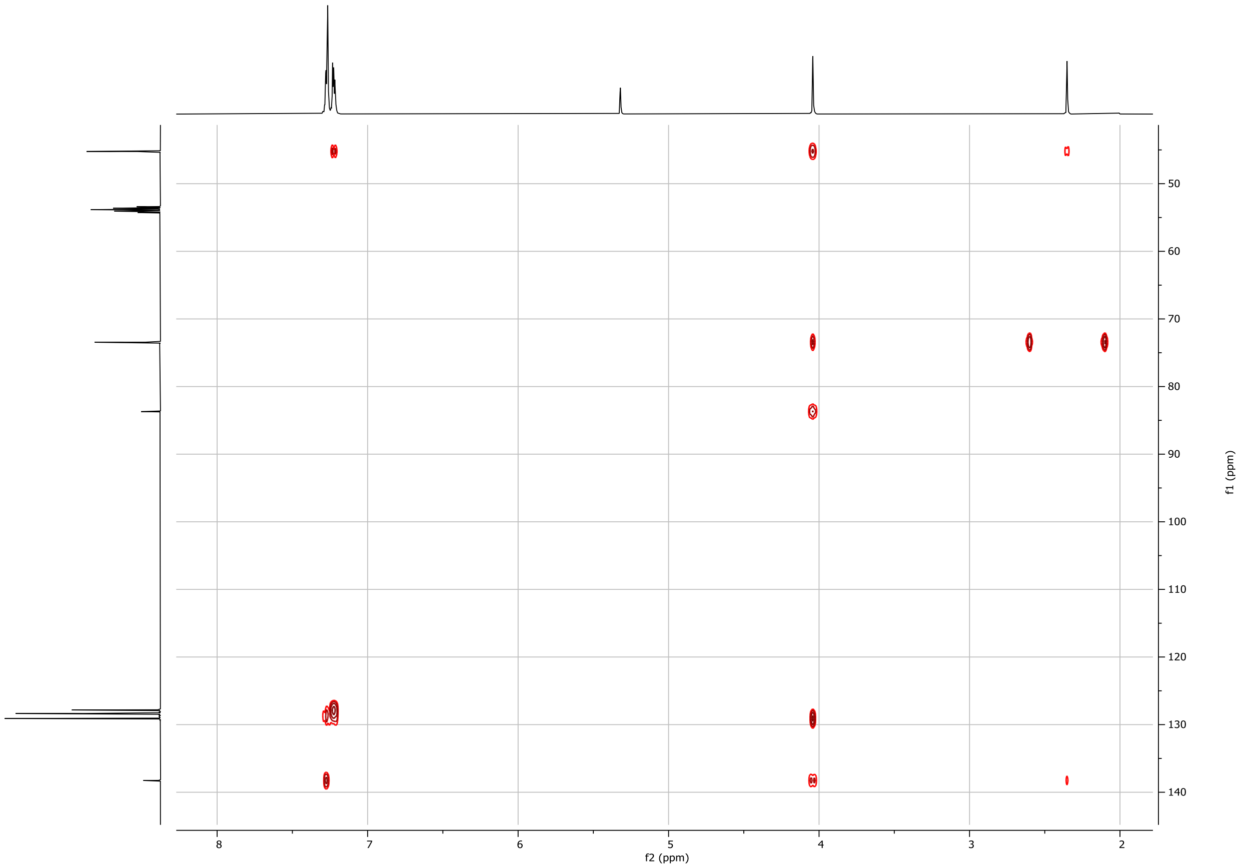

Supplementary Figure S16. HMBC NMR, CD_2Cl_2 of compound **meso-7**

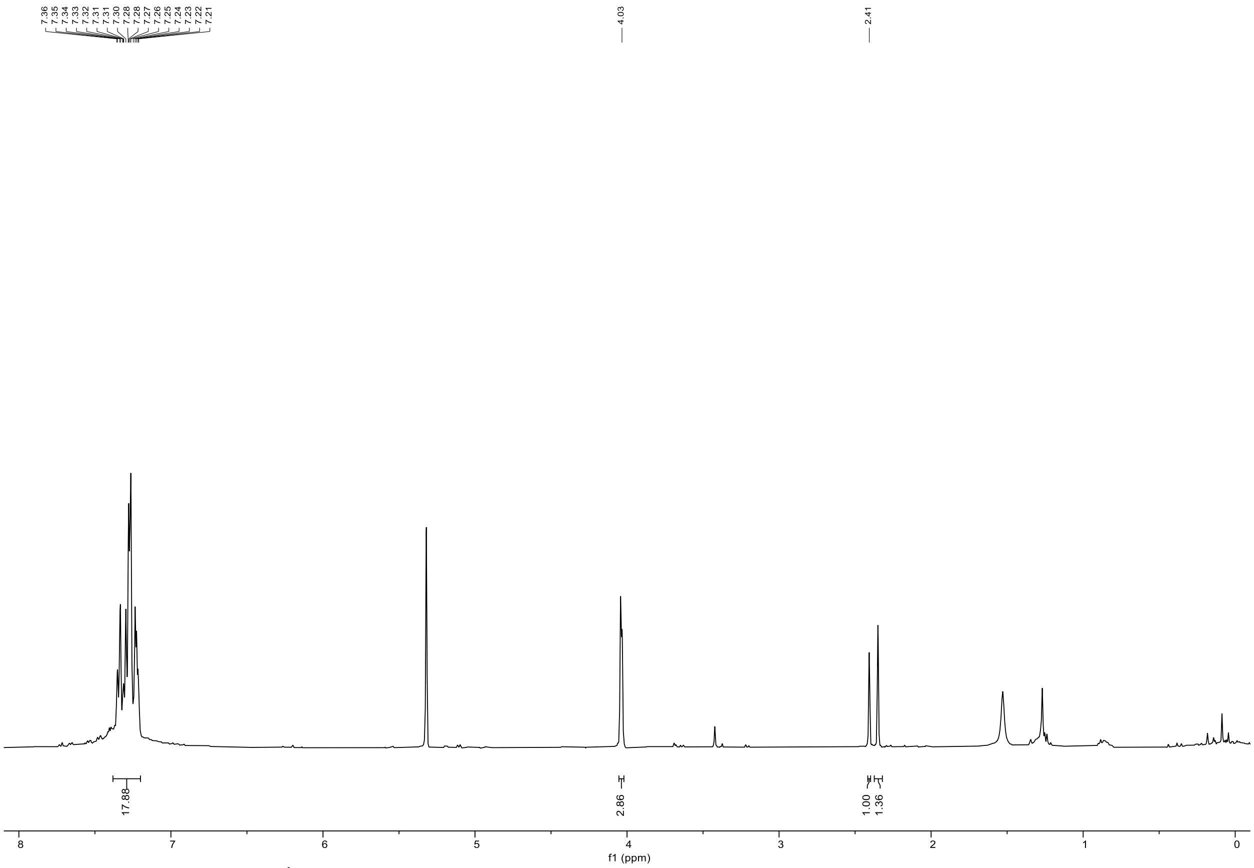

Supplementary Figure S17. ^{1}H NMR, 400 MHz, CD_2Cl_2 of compound **rac-7**

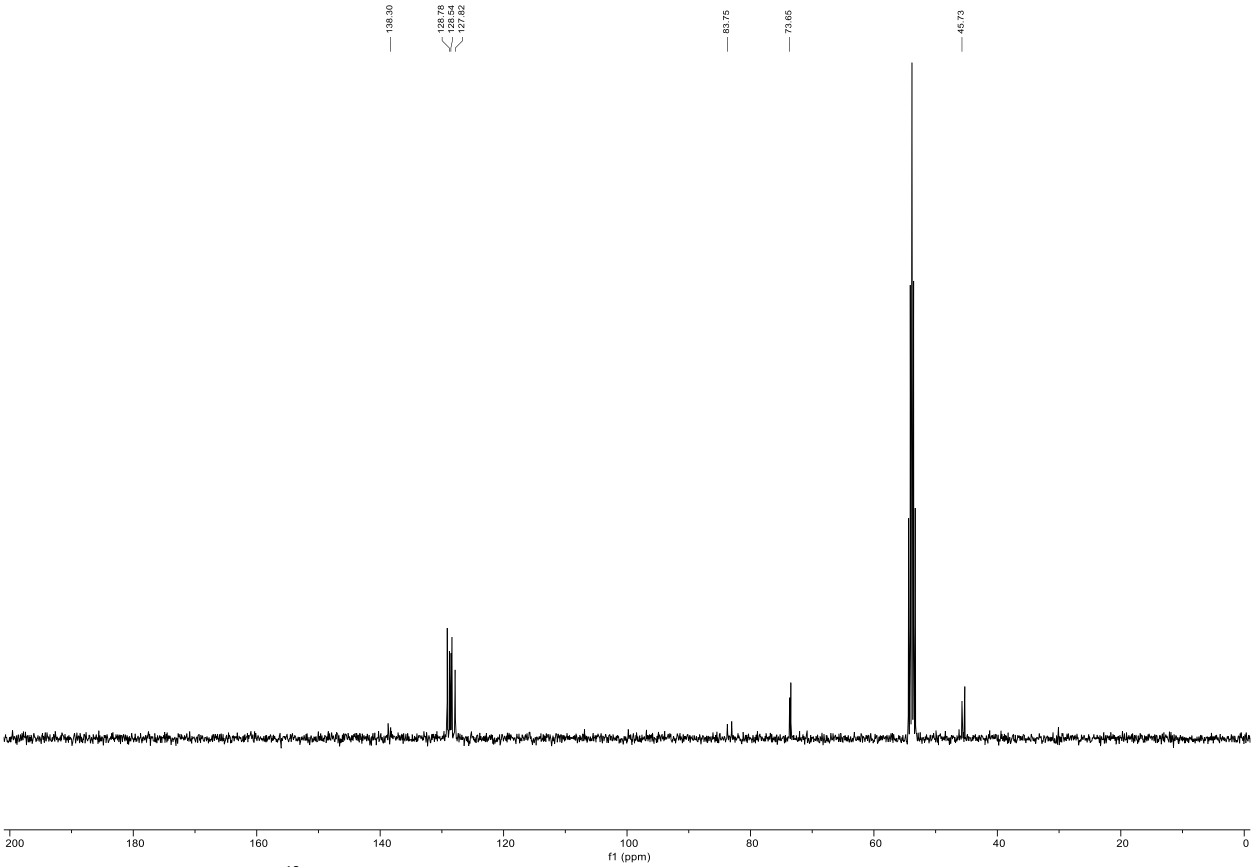

Supplementary Figure S18. ^{13}C NMR, 101 MHz, CD_2Cl_2 of compound **rac-7**

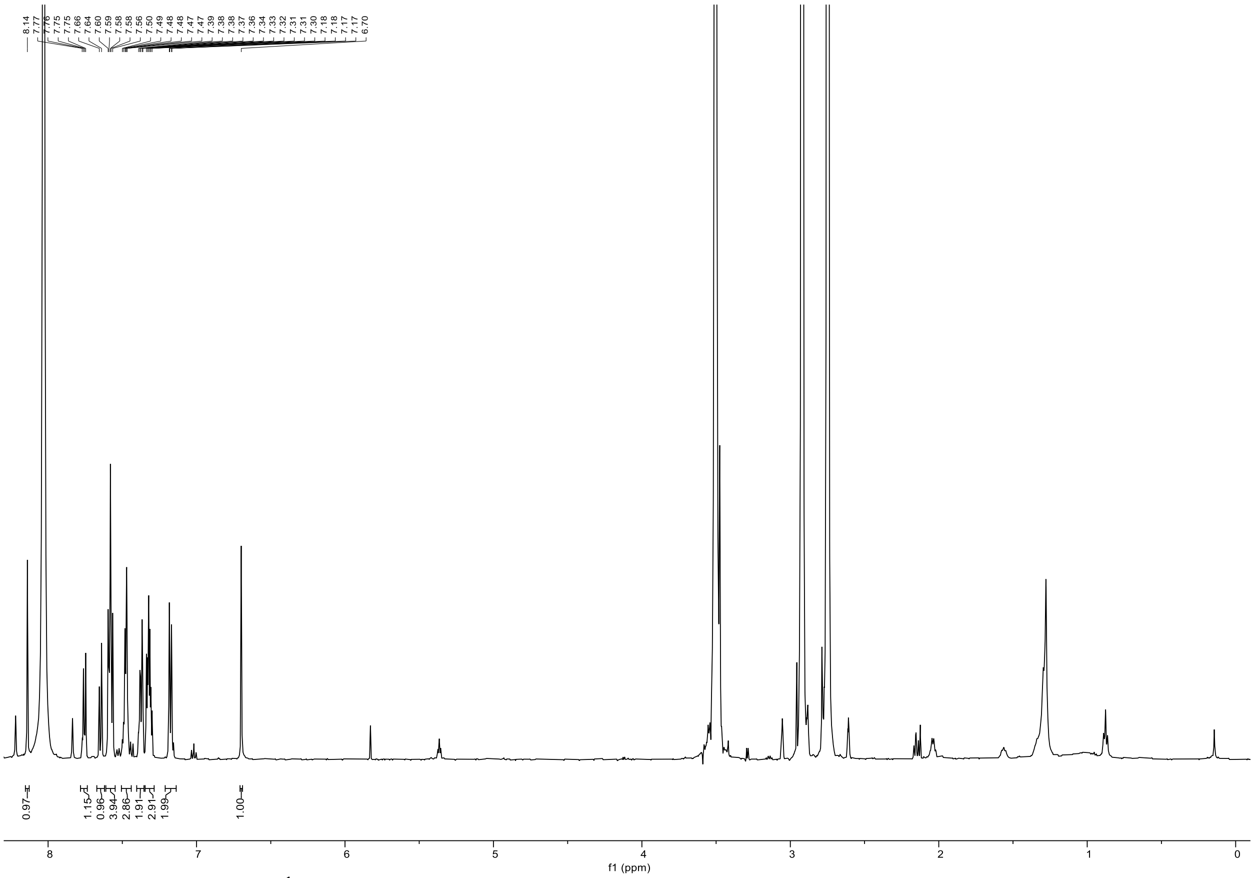

Supplementary Figure S19. ^{1}H NMR, 500 MHz, DMF-d_7 of compound ***EZ*-3**

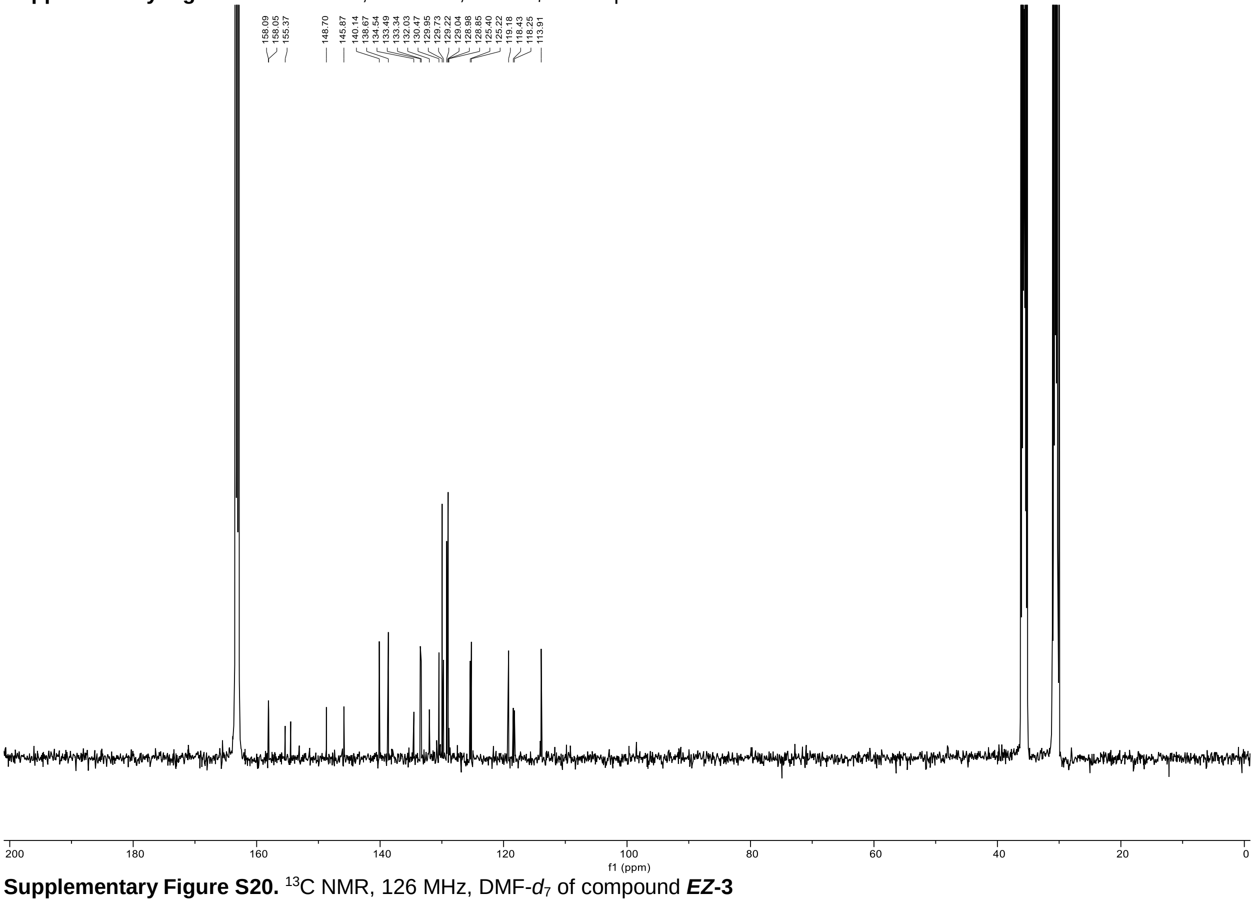

Supplementary Figure S20. ^{13}C NMR, 126 MHz, DMF-d_7 of compound ***EZ*-3**

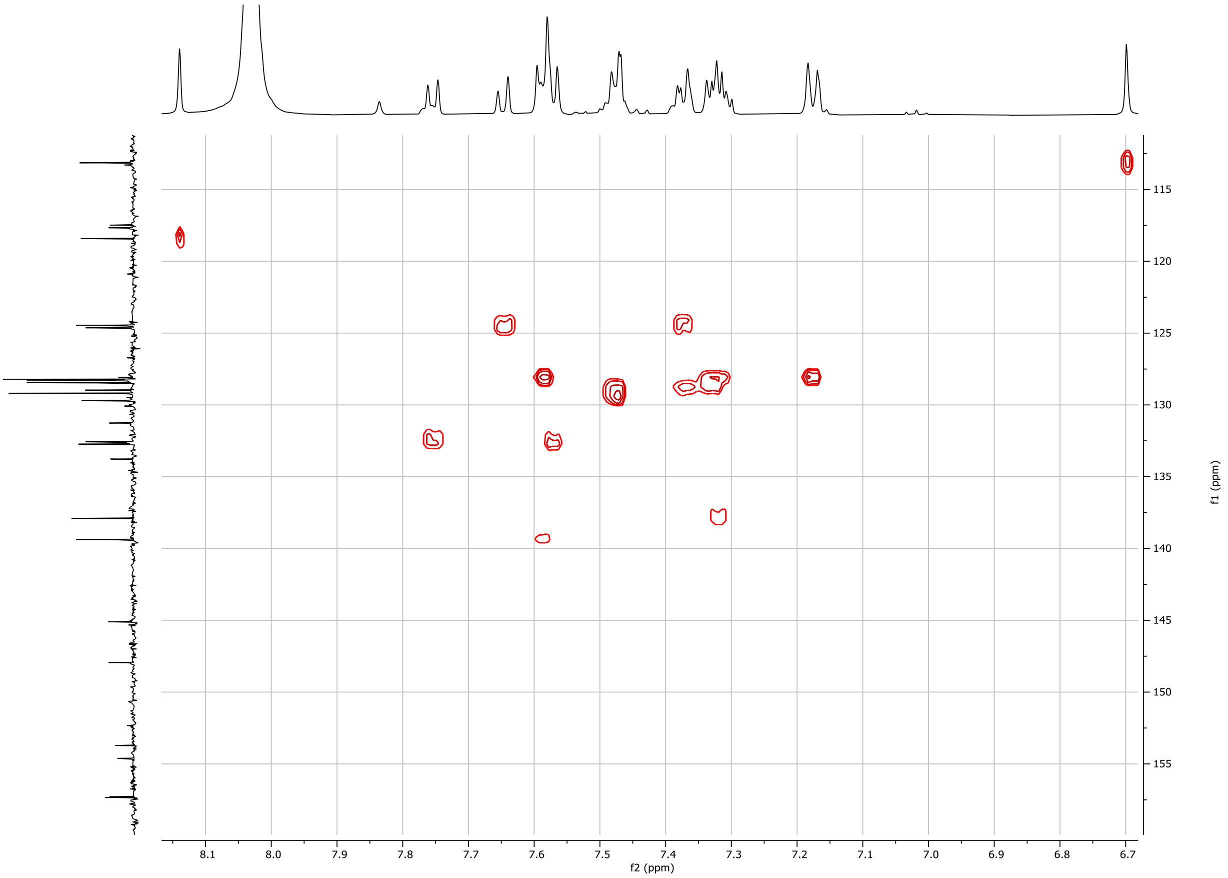

Supplementary Figure S21. HSQC NMR, DMF-d_7 of compound ***EZ*-3**

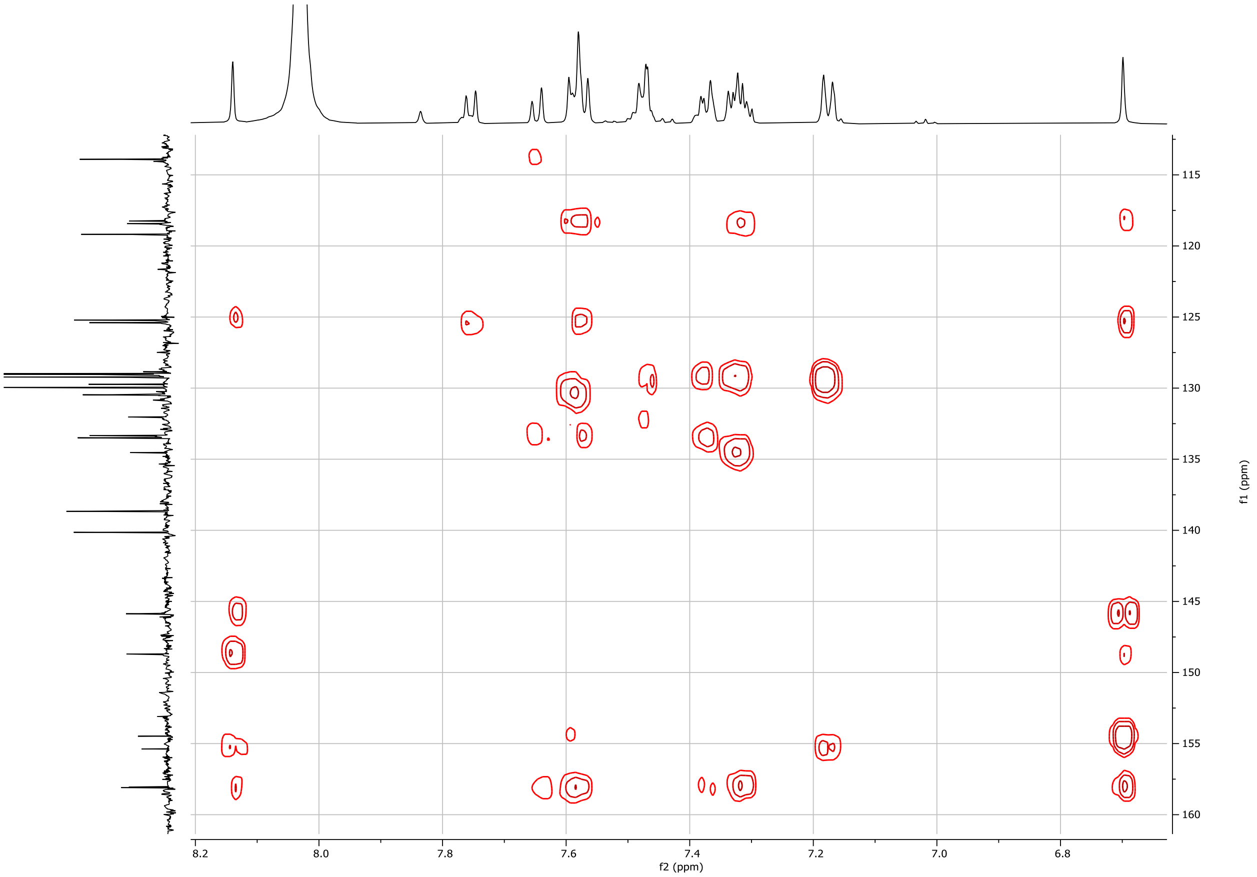

Supplementary Figure S22. HMBC NMR, DMF-d_7 of compound ***EZ*-3**

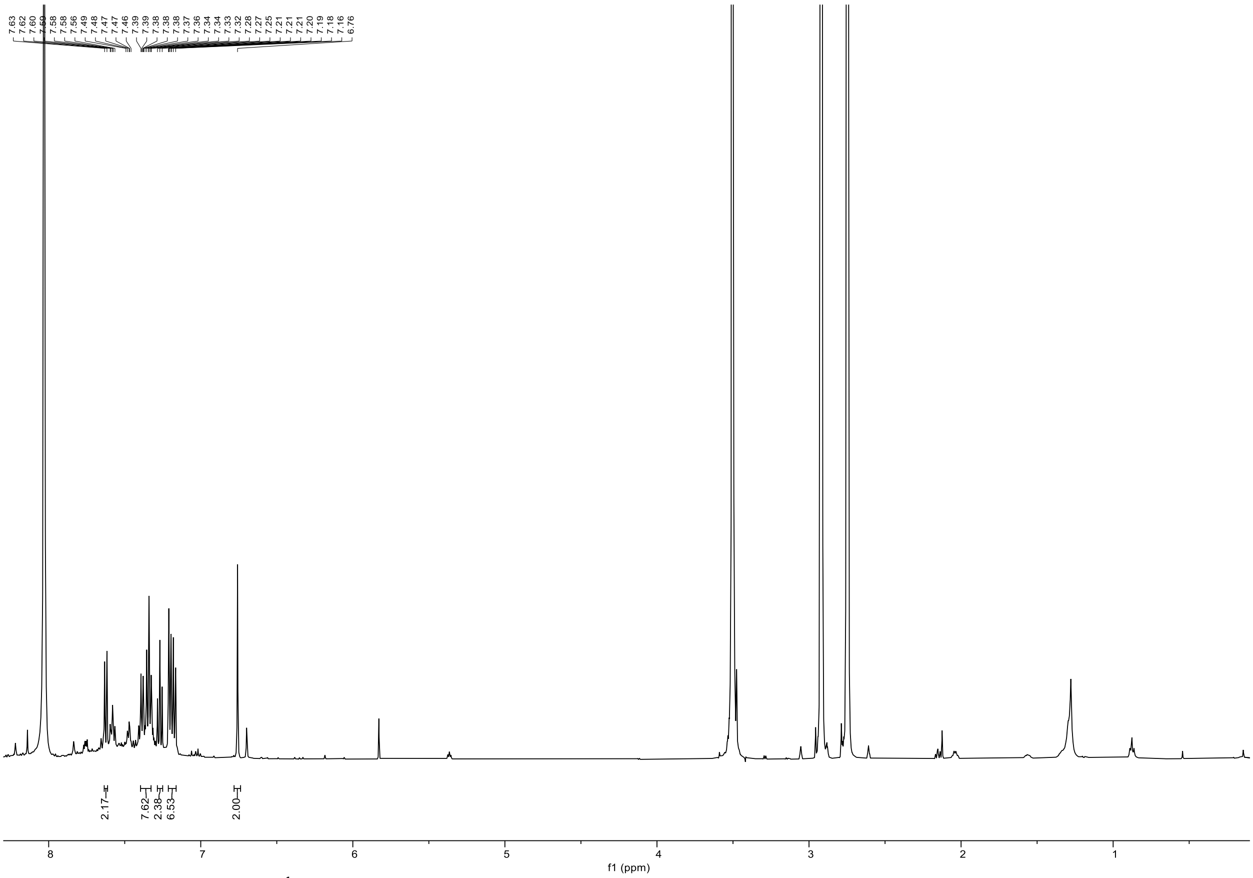

Supplementary Figure S23. ^{1}H NMR, 500 MHz, DMF-d_7 of compound ***ZZ*-3**

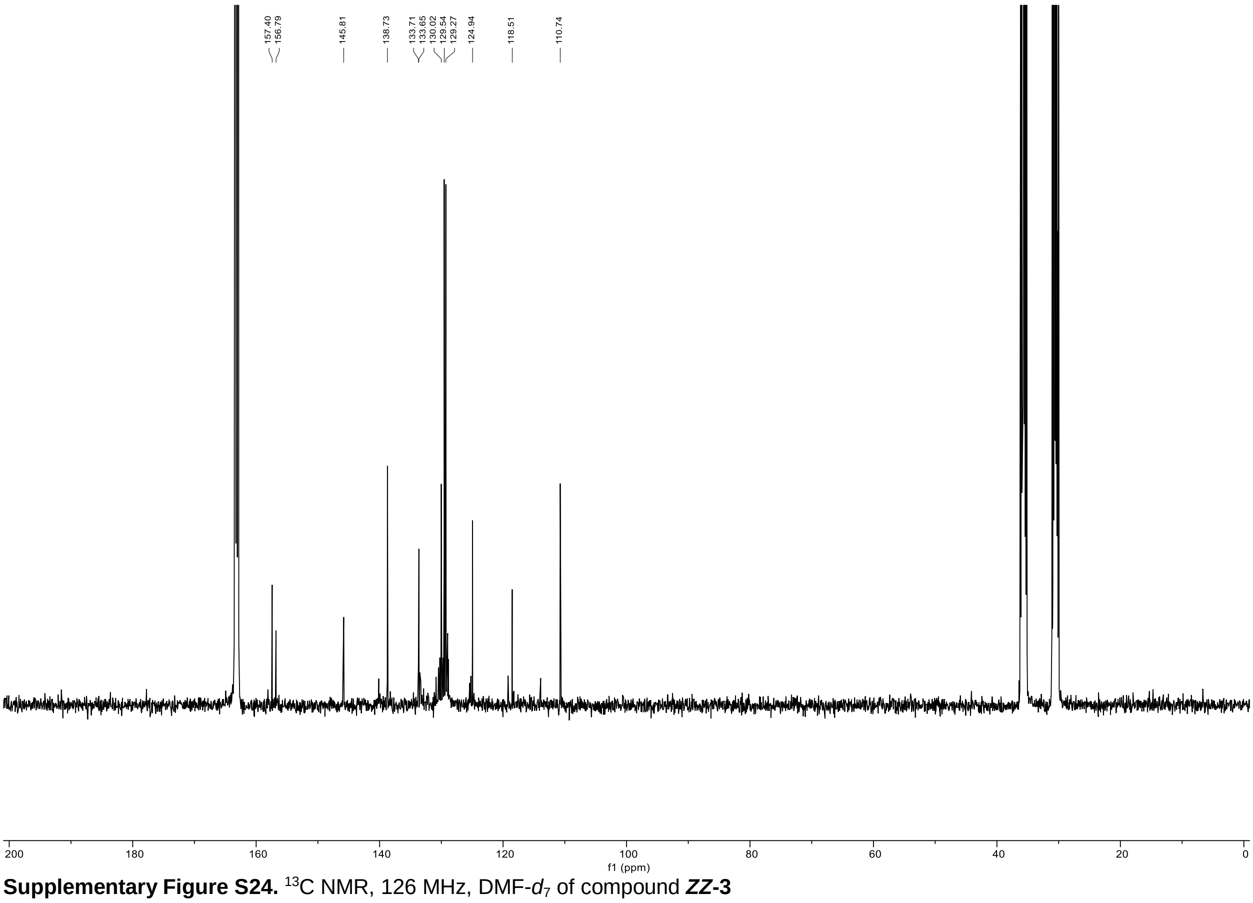

Supplementary Figure S24. ^{13}C NMR, 126 MHz, DMF-d_7 of compound ***ZZ*-3**

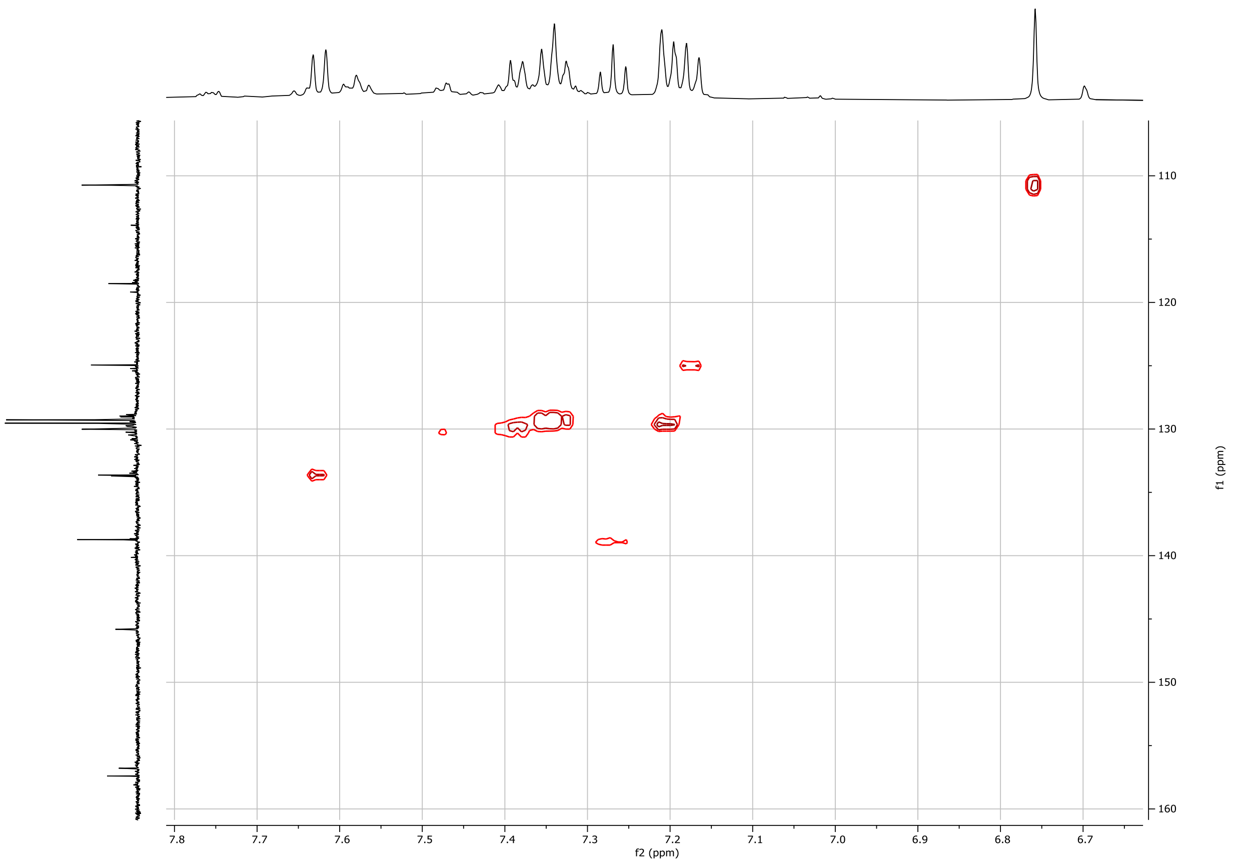

Supplementary Figure S25. HSQC NMR, DMF-d_7 of compound ***ZZ*-3**

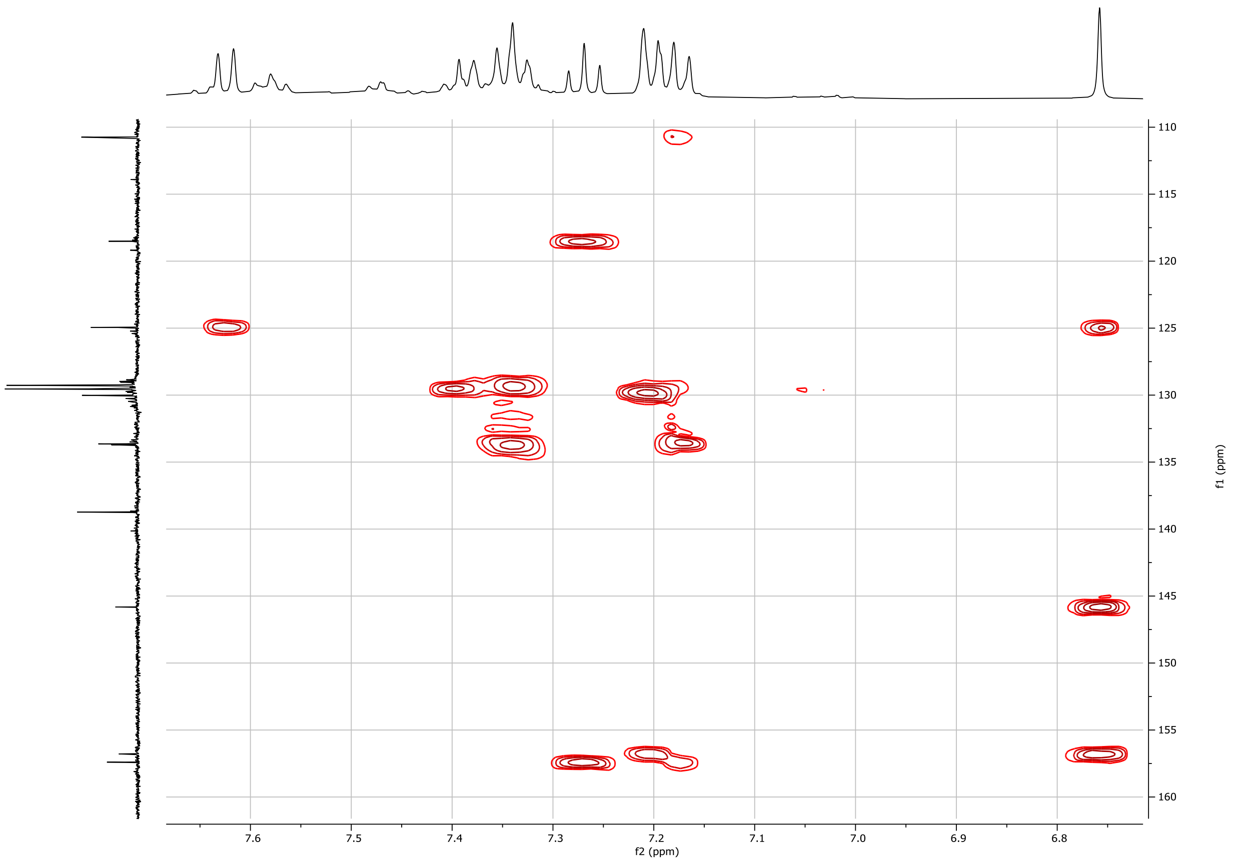

Supplementary Figure S26. HMBC NMR, DMF-d_7 of compound ***ZZ*-3**

Supplementary X-ray Methods

Methods for recording the crystal structure of *EZ*-3

A specimen of $C_{28}H_{18}I_2N_2$, approximate dimensions 0.120 mm x 0.180 mm x 0.320 mm, was used for the X-ray crystallographic analysis. The X-ray intensity data were measured (λ = 0.71073 Å). All recorded data is given in Figure 2, Supplementary Figure S23 and Supplementary Table S1-S8.

A total of 1818 frames were collected. The total exposure time was 2.52 hours. The frames were integrated with the Bruker SAINT software package using a narrow-frame algorithm. The integration of the data using an orthorhombic unit cell yielded a total of 23450 reflections to a maximum θ angle of 25.02° (0.84 Å resolution), of which 4068 were independent (average redundancy 5.765, completeness = 99.3%, Rint = 3.66%, Rsig = 2.77%) and 3636 (89.38%) were greater than 2σ(F2). The final cell constants of a = 30.5419(11) Å, b = 6.5237(2) Å, c = 11.8638(4) Å, volume = 2363.82(14) Å3, are based upon the refinement of the XYZ-centroids of 8398 reflections above 20 σ(I) with 6.245° < 2θ < 45.36°. Data were corrected for absorption effects using the Multi-Scan method (SADABS). The ratio of minimum to maximum apparent transmission was 0.848. The calculated minimum and maximum transmission coefficients (based on crystal size) are 0.4810 and 0.7390.

The structure was solved and refined using the Bruker SHELXTL Software Package, using the space group P c a 21, with Z = 4 for the formula unit, C28H18I2N2. The final anisotropic full-matrix least-squares refinement on F2 with 289 variables converged at R1 = 3.00%, for the observed data and wR2 = 9.74% for all data. The goodness-of-fit was 0.764. The largest peak in the final difference electron density synthesis was 0.572 e-/Å3 and the largest hole was -0.260 e-/Å3 with an RMS deviation of 0.061 e-/Å3. On the basis of the final model, the calculated density was 1.788 g/cm3 and F(000), 1224 e-.

Full details of data collection and structure solution and refinement are available on request from Cambridge Crystallographic Data Centre (CCDC) following the link https://www.ccdc.cam.ac.uk citing the deposition number 2429445.

Supplementary X-ray Tables and Figures

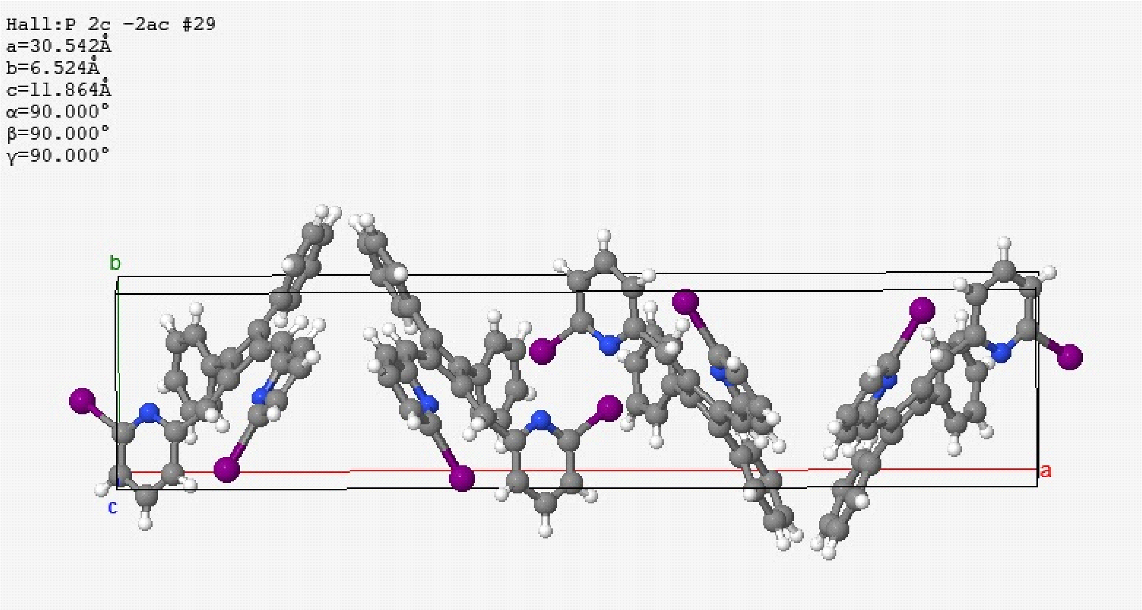

Supplementary Figure S27. Packing of the FKSS molecules in the structure

Supplementary Table S1. Data collection details for ***EZ*-3**.

Axis	Dx /mm	2θ /°	Ω /°	Φ /°	X /°	Width /°	Frames	Time/s	Wavelength /Å	Voltage /kV	Current /mA	Temp. /K
Omega	59.307	-28.00	-28.00	0.00	54.76	0.30	606	5.00	0.71076	50	20.0	n/a
Omega	59.307	-28.00	-28.00	90.00	54.76	0.30	606	5.00	0.71076	50	20.0	n/a
Omega	59.307	-28.00	-28.00	180.00	54.76	0.30	606	5.00	0.71076	50	20.0	n/a

Supplementary Table S2. Sample and crystal data for FKS_240226.

Identification code	FKS_240226
Chemical formula	$C_{28}H_{18}I_2N_2$
Formula weight	636.24 g/mol
Temperature	295(2) K
Wavelength	0.71073 Å
Crystal size	0.120 x 0.180 x 0.320 mm
Crystal system	orthorhombic
Space group	P c a 21
Unit cell dimensions	a = 30.5419(11) Å, α = 90°
	b = 6.5237(2) Å, β = 90°
	c = 11.8638(4) Å, γ = 90°
Volume	2363.82(14) Å3
Z	4
Density (calculated)	1.788 g/cm^3
Absorption coefficient	2.679 mm^{-1}
F(000)	1224

Supplementary Table S3. Data collection and structure refinement for FKS_240226.

Theta range for data collection	3.12 to 25.02°
Index ranges	-36<=h<=36, -7<=k<=7, -13<=l<=14
Reflections collected	23450
Independent reflections	4068 [R(int) = 0.0366]
Coverage of independent reflections	99.3%
Absorption correction	Multi-Scan
Max. and min. transmission	0.7390 and 0.4810
Structure solution technique	direct methods
Structure solution program	SHELXS-97 (Sheldrick 2008)
Refinement method	Full-matrix least-squares on F2
Refinement program	SHELXL-2019/1 (Sheldrick, 2019)
Function minimized	Σ w(Fo2 - Fc2)2
Data / restraints / parameters	4066 / 1 / 289
Goodness-of-fit on F2	1.038
Final R indices	3636 data; I>2σ(I)
all data	R1 = 0.0354, wR2 = 0.0684
Weighting scheme	w=1/[\s^2^(Fo^2^)+(0.0404P)^2^+0.0395P] where
	P=(Fo^2^+2Fc^2^)/3
Absolute structure parameter	-0.009(11)
Largest diff. peak and hole	0.481 and -0.241 eÅ-3
R.M.S. deviation from mean	0.057 eÅ-3

Supplementary Table S4. Atomic coordinates and equivalent isotropic atomic displacement parameters (Å2) for FKS_240226. U(eq) is defined as one third of the trace of the orthogonalized Uij tensor.

x/a	y/b	z/c	U(eq)
I001	0.46275(2)	0.62532(9)	0.25640(5)
I002	0.62122(2)	0.92325(10)	0.85052(7)
N003	0.53616(19)	0.6652(10)	0.4163(6)
N004	0.6588(2)	0.5653(10)	0.7396(6)
C005	0.6251(2)	0.3995(10)	0.3761(6)
C006	0.5073(2)	0.7802(11)	0.3623(7)
C007	0.5648(2)	0.7629(12)	0.4840(6)
C008	0.6558(2)	0.2561(11)	0.4085(7)
C009	0.6570(3)	0.6491(13)	0.8418(8)
C00A	0.6223(2)	0.4915(12)	0.4887(7)
C00B	0.6823(3)	0.3919(13)	0.7264(7)
C00C	0.5990(3)	0.6406(12)	0.5394(7)
C00D	0.5041(3)	0.9926(13)	0.3732(7)
C00E	0.6575(2)	0.3402(12)	0.5251(7)
C00F	0.5621(3)	0.9701(13)	0.5042(8)
C00G	0.6040(2)	0.4475(11)	0.2674(7)
C00H	0.7045(3)	0.3044(16)	0.8171(8)
C00I	0.6821(3)	0.2963(14)	0.6161(8)
C00J	0.5860(3)	0.2920(13)	0.2014(7)
C00K	0.7013(3)	0.3938(16)	0.9209(9)
C00L	0.6794(2)	0.0929(11)	0.3526(10)
C00M	0.6025(3)	0.6473(13)	0.2286(7)
C00N	0.5822(3)	0.6914(15)	0.1283(8)
C00O	0.5664(3)	0.3372(15)	0.1007(8)
C00P	0.5324(3)	0.0881(13)	0.4442(12)
C00Q	0.6766(3)	0.5654(16)	0.9362(9)
C00R	0.6881(3)	0.1037(13)	0.2371(10)
C00S	0.7153(3)	0.7636(15)	0.3556(15)
C00T	0.5646(3)	0.5405(16)	0.0644(8)
C00U	0.7233(3)	0.7773(17)	0.2429(14)
C00V	0.7096(3)	0.9488(17)	0.1813(11)
C00W	0.6935(3)	0.9219(13)	0.4091(11)

Supplementary Table S5. Bond lengths (Å) for FKS_240226.

I001-C006	2.110(8)	I002-C009	2.098(9)
N003-C006	1.323(10)	N003-C007	1.347(10)
N004-C009	1.331(12)	N004-C00B	1.348(11)
C005-C008	1.379(11)	C005-C00A	1.467(11)
C005-C00G	1.475(11)	C006-C00D	1.395(12)
C007-C00F	1.375(12)	C007-C00C	1.471(11)
C008-C00L	1.448(11)	C008-C00E	1.489(11)
C009-C00Q	1.383(14)	C00A-C00C	1.347(12)
C00A-C00E	1.520(10)	C00B-C00H	1.395(12)
C00B-C00I	1.450(13)	C00D-C00P	1.358(15)
C00E-C00I	1.348(12)	C00F-C00P	1.388(14)
C00G-C00M	1.383(11)	C00G-C00J	1.394(11)
C00H-C00K	1.366(15)	C00J-C00O	1.369(13)
C00K-C00Q	1.363(14)	C00L-C00W	1.371(13)
C00L-C00R	1.396(17)	C00M-C00N	1.373(12)
C00N-C00T	1.354(15)	C00O-C00T	1.396(15)
C00R-C00V	1.375(13)	C00S-C00U	1.36(2)
C00S-C00W	1.383(16)	C00U-C00V	1.401(19)

Supplementary Table S6. Bond angles (°) for FKS_240226.

Bond angle atoms	Bond angle	Bond angle atoms	Bond angle
C006-N003-C007	116.9(7)	C009-N004-C00B	118.2(7)
C008-C005-C00A	93.6(6)	C008-C005-C00G	133.2(7)
C00A-C005-C00G	133.2(7)	N003-C006-C00D	124.4(7)
N003-C006-I001	116.5(5)	C00D-C006-I001	119.0(5)
N003-C007-C00F	122.1(7)	N003-C007-C00C	118.1(7)
C00F-C007-C00C	119.8(7)	C005-C008-C00L	135.3(8)
C005-C008-C00E	91.9(6)	C00L-C008-C00E	132.8(8)
N004-C009-C00Q	123.8(8)	N004-C009-I002	114.6(6)
C00Q-C009-I002	121.5(7)	C00C-C00A-C005	137.1(7)
C00C-C00A-C00E	135.6(8)	C005-C00A-C00E	87.3(6)
N004-C00B-C00H	120.9(8)	N004-C00B-C00I	117.7(7)
C00H-C00B-C00I	121.4(8)	C00A-C00C-C007	124.6(8)
C00P-C00D-C006	117.9(8)	C00I-C00E-C008	133.3(7)
C00I-C00E-C00A	139.5(8)	C008-C00E-C00A	87.2(6)
C007-C00F-C00P	119.6(8)	C00M-C00G-C00J	119.1(7)
C00M-C00G-C005	120.4(7)	C00J-C00G-C005	120.6(7)
C00K-C00H-C00B	119.1(9)	C00E-C00I-C00B	129.3(8)
C00O-C00J-C00G	120.4(8)	C00Q-C00K-C00H	120.7(9)
C00W-C00L-C00R	117.5(9)	C00W-C00L-C008	122.0(11)
C00R-C00L-C008	120.4(8)	C00N-C00M-C00G	120.1(8)
C00T-C00N-C00M	120.8(9)	C00J-C00O-C00T	119.4(9)
C00D-C00P-C00F	118.7(8)	C00K-C00Q-C009	117.2(10)
C00V-C00R-C00L	121.7(10)	C00U-C00S-C00W	119.2(11)
C00N-C00T-C00O	120.2(9)	C00S-C00U-C00V	120.7(10)
C00R-C00V-C00U	118.6(12)	C00L-C00W-C00S	122.3(13)

Supplementary Table S7. Anisotropic atomic displacement parameters (Å2) for FKS_240226. The anisotropic atomic displacement factor exponent takes the form: -2π2[h2 a*2 U11 + ... + 2 h k a* b* U12]

U11	U22	U33	U23	U13	U12
I001	0.0617(3)	0.0682(3)	0.0547(3)	0.0044(3)	-0.0132(3)
I002	0.0777(4)	0.0636(3)	0.0732(4)	-0.0057(3)	0.0102(4)
N003	0.050(4)	0.043(3)	0.042(4)	0.004(3)	-0.003(3)
N004	0.040(3)	0.062(4)	0.053(4)	0.004(3)	-0.006(3)
C005	0.044(4)	0.043(4)	0.040(5)	0.001(3)	-0.006(3)
C006	0.037(3)	0.057(4)	0.049(4)	0.013(4)	0.004(3)
C007	0.037(3)	0.053(4)	0.035(4)	0.000(3)	0.005(3)
C008	0.044(4)	0.045(4)	0.050(4)	-0.001(3)	0.004(3)
C009	0.046(4)	0.068(5)	0.053(5)	-0.003(5)	-0.006(4)
C00A	0.036(4)	0.045(4)	0.046(4)	0.004(3)	-0.007(3)
C00B	0.038(4)	0.061(4)	0.051(5)	0.006(3)	-0.002(3)
C00C	0.042(4)	0.058(5)	0.045(4)	0.001(4)	-0.001(3)
C00D	0.047(4)	0.055(4)	0.059(6)	0.012(4)	0.000(4)
C00E	0.041(4)	0.046(4)	0.052(4)	0.001(3)	-0.002(3)
C00F	0.051(4)	0.050(4)	0.062(5)	-0.009(4)	0.003(4)
C00G	0.041(4)	0.054(4)	0.035(4)	-0.004(4)	-0.002(3)
C00H	0.046(4)	0.075(6)	0.056(5)	0.004(4)	-0.012(4)
C00I	0.040(4)	0.064(5)	0.059(5)	0.003(4)	-0.007(3)
C00J	0.064(5)	0.046(4)	0.049(5)	-0.008(3)	-0.005(4)
C00K	0.054(5)	0.094(7)	0.056(6)	0.020(5)	-0.016(4)
C00L	0.039(4)	0.054(4)	0.065(5)	-0.010(4)	-0.003(4)
C00M	0.054(4)	0.051(4)	0.043(5)	0.000(3)	0.000(3)
C00N	0.082(6)	0.057(5)	0.047(5)	0.005(4)	0.002(4)
C00O	0.069(6)	0.070(6)	0.052(5)	-0.016(4)	-0.006(4)
C00P	0.060(5)	0.040(4)	0.100(8)	-0.001(5)	-0.002(5)
C00Q	0.055(5)	0.088(7)	0.050(5)	0.002(4)	-0.005(4)
C00R	0.049(4)	0.056(4)	0.080(8)	-0.011(4)	-0.002(4)
C00S	0.068(5)	0.058(5)	0.119(9)	-0.011(7)	-0.025(7)
C00T	0.088(7)	0.085(7)	0.043(5)	-0.003(5)	-0.012(4)
C00U	0.059(5)	0.067(6)	0.141(13)	-0.037(8)	-0.002(7)
C00V	0.061(5)	0.084(7)	0.085(8)	-0.035(6)	0.015(5)
C00W	0.051(5)	0.059(5)	0.088(7)	-0.002(5)	-0.020(5)

Supplementary Table S8. Hydrogen atomic coordinates and isotropic atomic displacement parameters (Å2) for FKS_240226.

x/a	y/b	z/c	U(eq)
H00C	0.6050	0.6681	0.6148
H00D	0.4832	1.0664	0.3330
H00E	0.5802	1.0307	0.5578
H00F	0.7212	0.1867	0.8070
H00G	0.7019	0.1891	0.6069
H00H	0.5874	0.1568	0.2259
H00I	0.7162	0.3370	0.9817
H00J	0.6153	0.7518	0.2705
H00K	0.5805	0.8266	0.1039
H00L	0.5543	0.2333	0.0569
H00M	0.5318	1.2299	0.4524
H00N	0.6731	0.6238	1.0071
H00O	0.6790	0.2186	0.1970
H00P	0.7244	-0.3509	0.3961
H00Q	0.5513	0.5724	-0.0039
H00R	0.7381	-0.3283	0.2065
H00S	0.7149	-0.0422	0.1042
H00T	0.6882	-0.0880	0.4861

Supplementary Computational Methods

Computational Details

Density functional theory (DFT) calculations were performed with Gaussian16 software.[1] Geometry optimizations were carried out using the M06-2X functional[2] including Grimme D3 dispersion correction[3] and 6-31G(d) basis set[4] in solution (n,n-dimethylformamide, ε=37.219) by the continuum method PCM.[5] The Stuttgart / Dresden effective core potential (SDD) and its associated basis set[6] were employed for I atom. Frequency analysis was performed at the same level under standard conditions to either a minimum (i.e., no imaginary frequency) or a transition state (i.e., only one imaginary frequency) and to obtain thermodynamic energy corrections. Intrinsic reaction coordinate (IRC) calculations[7] were conducted to verify that all transition state structures connected the corresponding reactants and products. Single-point calculations with the M06-2X/6-311++G(d,p) level of theory[8,9] and SMD solvent model[10] with corrected Coloumb radii for I[11] were performed using the geometries obtained at optimization step. The free energies presented in this work represent M06-2X/6-311++G(d,p) calculated single-point energies with M06-2X/6-31G(d) calculated thermodynamic corrections at 298 K.

Conformational Sampling of Stationary Points

For the key reactants and products, we employed the CREST molecular suite[12] to facilitate conformational searches at the GFN2-xTB[13] level to identify the lowest-energy conformers, which were then refined at the DFT level described in the general methods. For intermediates and transition states, we relied on a combination of chemical intuition, mechanistic reasoning, and manual sampling of torsional degrees of freedom to explore plausible conformational variants. any of the stationary points, particularly intermediates, were obtained directly as reactant– or product- complexes along the intrinsic reaction coordinate (IRC) paths, and thus represent chemically meaningful and dynamically connected structures. For each critical structure, multiple conformers were optimized and compared, and only the lowest-energy conformers were used to construct the final potential energy surface.

Supplementary Computational Tables

Supplementary Table S9. Energy values for the reported species and imaginary frequencies for the transition states

Structures	Corr. to *G*[a]	SP[b]	*G*[c]	IF[d]
**rac-1**	0.312727	-1209.780488	-1209.467761	—
**NEt_3**	0.175187	-292.3456492	-292.1704622	—
**TS-1**	0.509126	-1502.115727	-1501.606601	–1293.85
**INT-1**	0.513225	-1502.136613	-1501.623388	—
**TS-R_a-2**	0.512840	-1502.12615	-1501.61331	–28.01
**TS-S_a-2**	0.509594	-1502.124756	-1501.615162	–439.76
**INT-R_a-2**	0.509103	-1502.14806	-1501.638957	—
**INT-S_a-2**	0.510845	-1502.152396	-1501.641551	—
**TS-R_a-3**	0.507379	-1502.127943	-1501.620564	–1299.48
**TS-S_a-3**	0.507225	-1502.126836	-1501.619611	–1280.82
**INT-S_a-3**	0.514682	-1502.148501	-1501.633819	—
**INT-R_a-3**	0.513513	-1502.146177	-1501.632664	—
**TS-R_aR_a-4**	0.509921	-1502.139307	-1501.629386	–55.91
**TS-R_aS_a-4**	0.508889	-1502.139496	-1501.630607	–309.50
**TS-S_aS_a-4**	0.507797	-1502.139139	-1501.631342	– 252.22
**TS-S_aR_a-4**	0.514288	-1502.138002	-1501.623714	– 9.65
**TS-S_aR_a-4*[e]**	0.511293	-1502.137018	-1501.625725	-30.21
**TS-S_aR_a-4**[f]**	0.513382	-1502.136595	-1501.623213	-610.12
**INT-R_aR_a-4**	0.312684	-1209.79699	-1209.484306	—
**INT-S_aR_a-4**	0.312255	-1209.797399	-1209.485144	—
**INT-S_aS_a-4**	0.311869	-1209.795869	-1209.484	—
**TS-R_aR_a-5**	0.314568	-1209.791289	-1209.476721	– 17.91
**TS-S_aS_a-5**	0.313727	-1209.791479	-1209.477752	– 20.38
**TS-S_aR_a-5**	0.314027	-1209.793231	-1209.479204	– 11.66
**INT-S_aS_a-5**	0.314067	-1209.80473	-1209.490663	—
**INT-S_aR_a-5**	0.313964	-1209.801427	-1209.487463	—
**INT-R_aR_a-5**	0.312517	-1209.79958	-1209.487063	—
**TS-R_aR_a-6**	0.313162	-1209.763332	-1209.45017	–439.44
**TS-S_aS_a-6**	0.314234	-1209.767169	-1209.452935	–551.96
**TS-S_aR_a-6**	0.313331	-1209.765215	-1209.451884	–455.13
***EE*-3**	0.318781	-1209.831762	-1209.512981	—
***ZZ*-3**	0.316725	-1209.832111	-1209.515386	—
***EZ*-3**	0.316857	-1209.8295	-1209.512643	—
**TS-7**	0.312162	-1209.72929	-1209.417128	–439.77
**INT-6**	0.312413	-1209.801186	-1209.488773	—
**TS-8**	0.312597	-1209.760502	-1209.447905	–450.94
**2**	0.316434	-1209.825757	-1209.509323	—

[a] Free energy correction of the structures calculated at M06-2X PCM (DMF)/6-31G(d), 298K, Hartree. [b] Single point energies at the M06-2X SMD-18 (DMF)/6-311+G(d,p) level of theory, Hartree. [c] Free energies = SP + Corr. to G, Hartree. [d] Imaginary frequencies of the transition states. [e] Geometry of the local maximum of the relaxed potential energy surface scan between **INT-S_a-3** and **INT-S_aR_a-4**, through **TS-S_aR_a-4** (0.05 Å steps). Computed at SMD-18(DMF)/M06-2X-D3/6-311+G(d,p)/SDD//PCM(DMF)//M06-2X-D3/6-31G(d)/SDD. [f] The geometry was computed at PCM(DMF)/M06-2X-D3/6-311G(d,p), with the reported energies computed at SMD-18(DMF)/M06-2X-D3/6-311+G(d,p)/SDD, frequency and single point correction computed at PCM(DMF)//M06-2X-D3/6-31G(d)/SDD.

Supplementary Table S10. Optimized cartesian coordinates of the calculated structures

rac-1

C	0.524616	0.724600	3.476551
C	1.742953	0.069152	3.644951
C	2.312662	-0.616332	2.573562
C	1.666348	-0.645118	1.340063
C	0.448260	0.016315	1.163133
C	-0.118873	0.699052	2.241595
H	0.073498	1.258531	4.306987
H	2.244057	0.089323	4.607569
H	3.259320	-1.133596	2.696460
H	2.116819	-1.174024	0.504908
H	-1.066546	1.214426	2.109930
C	-0.198723	0.030467	-0.212158
H	0.089869	-0.884330	-0.742617
C	0.318065	1.232104	-1.103370
C	1.753820	1.066276	-1.335340
C	2.940253	0.881787	-1.471355
C	-1.659784	0.087428	-0.146080
C	-2.862681	0.194567	-0.102507
C	4.351698	0.651813	-1.608379
C	5.120023	1.397767	-2.504070
N	4.882104	-0.310367	-0.825406
C	6.483745	1.135494	-2.586762
H	4.650928	2.160878	-3.113436
C	6.170120	-0.529472	-0.929845
C	7.040574	0.147345	-1.783098
H	7.110641	1.696495	-3.271587
H	8.097017	-0.087068	-1.815628
C	-4.282840	0.403002	-0.040182
C	-4.785672	1.682988	0.206963
N	-5.081077	-0.666070	-0.230493
C	-6.164620	1.855534	0.256656
H	-4.100183	2.510264	0.354350
C	-6.375970	-0.465796	-0.175281
C	-6.997493	0.759251	0.061978
H	-6.590374	2.835223	0.445719
H	-8.075906	0.849663	0.092634
I	6.976122	-2.069874	0.341673
I	-7.617845	-2.198362	-0.482352
C	-0.026867	2.582185	-0.498678
C	0.817050	3.198615	0.427872
C	-1.237944	3.195413	-0.830169
C	0.452485	4.405804	1.019334
H	1.761118	2.726872	0.687405
C	-1.604305	4.402402	-0.238194
H	-1.896612	2.723839	-1.555028
C	-0.759878	5.009030	0.690269
H	1.116891	4.874427	1.738421
H	-2.545579	4.871124	-0.508723
H	-1.043000	5.950392	1.150335
H	-0.199978	1.139090	-2.065207

TS-7

C	-4.552252	3.798638	-1.176488
C	-5.940136	3.687887	-1.282414
C	-6.530619	2.427667	-1.346298
C	-5.738452	1.282821	-1.305775
C	-4.346432	1.387139	-1.201224
C	-3.759734	2.657789	-1.134324
H	-4.087998	4.778502	-1.126399
H	-6.556161	4.580651	-1.316040
H	-7.608594	2.334065	-1.431137
H	-6.196837	0.298410	-1.357594
H	-2.679232	2.740467	-1.049141
C	-3.533483	0.156984	-1.104520
H	-4.072700	-0.773442	-1.276806
C	-3.533485	-0.156979	1.104519
C	-2.190505	-0.142774	1.376855
C	-0.978107	-0.070327	1.096602
C	-2.190503	0.142783	-1.376854
C	-0.978105	0.070334	-1.096598
C	0.418066	-0.074356	1.480150
C	1.004369	-1.223798	2.013667
N	1.124143	1.044407	1.225226
C	2.369820	-1.212358	2.276408
H	0.397549	-2.102858	2.196715
C	2.409769	1.018396	1.480081
C	3.111484	-0.069545	1.996383
H	2.859297	-2.091272	2.682761
H	4.179541	-0.025025	2.168336
C	0.418068	0.074362	-1.480145
C	1.004374	1.223805	-2.013655
N	1.124142	-1.044403	-1.225224
C	2.369826	1.212364	-2.276392
H	0.397557	2.102867	-2.196699
C	2.409769	-1.018395	-1.480077
C	3.111487	0.069548	-1.996372
H	2.859307	2.091280	-2.682739
H	4.179544	0.025026	-2.168323
I	3.498741	2.826657	1.053211
I	3.498736	-2.826660	-1.053215
C	-4.346428	-1.387139	1.201221
C	-3.759726	-2.657785	1.134300
C	-5.738447	-1.282828	1.305794
C	-4.552237	-3.798638	1.176464
H	-2.679224	-2.740458	1.049099
C	-6.530608	-2.427677	1.346318
H	-6.196835	-0.298419	1.357630
C	-5.940120	-3.687894	1.282412
H	-4.087980	-4.778500	1.126357
H	-7.608582	-2.334080	1.431173
H	-6.556141	-4.580661	1.316038
H	-4.072706	0.773444	1.276804

INT-6

C	0.633421	-0.226987	0.715701
C	0.633395	0.226826	-0.715719
C	1.641699	-0.141725	-1.475558
C	1.641720	0.141612	1.475525
C	2.696173	0.546579	2.140982
C	2.696159	-0.546657	-2.141025
C	-0.476504	-1.087699	1.190636
C	-0.476582	1.087476	-1.190646
C	-0.654525	-1.387184	2.543000
C	-1.719429	-2.198899	2.911169
C	-2.586055	-2.683840	1.934120
C	-2.306207	-2.313059	0.623128
C	-0.654789	1.386716	-2.543038
C	-1.719737	2.198373	-2.911207
C	-2.586217	2.683503	-1.934124
C	-2.306183	2.312967	-0.623102
H	2.613676	1.450876	2.744420
H	0.028897	-0.983050	3.281622
H	-1.883842	-2.450252	3.953804
H	-3.432672	-3.311992	2.180181
H	0.028523	0.982434	-3.281681
H	-1.884299	2.449530	-3.953866
H	-3.432868	3.311613	-2.180178
N	-1.304080	-1.557371	0.242866
I	-3.610272	-3.013307	-0.945289
I	-3.610003	3.013565	0.945365
N	-1.304017	1.557328	-0.242841
H	2.613685	-1.450964	-2.744453
C	4.021362	-0.103696	2.094563
C	5.054832	0.386489	2.899032
C	4.285069	-1.163952	1.216505
C	6.329130	-0.172198	2.830247
H	4.858436	1.214071	3.575312
C	5.555520	-1.721172	1.149977
H	3.490384	-1.530273	0.571041
C	6.582923	-1.226428	1.956098
H	7.123611	0.219738	3.457350
H	5.749981	-2.535969	0.459418
H	7.576780	-1.658513	1.897659
C	4.021328	0.103657	-2.094618
C	5.054786	-0.386446	-2.899151
C	4.285027	1.163876	-1.216513
C	6.329064	0.172289	-2.830388
H	4.858396	-1.214002	-3.575466
C	5.555458	1.721145	-1.150007
H	3.490353	1.530128	-0.570995
C	6.582848	1.226485	-1.956195
H	7.123536	-0.219585	-3.457541
H	5.749914	2.535913	-0.459413

H 7.576689 1.658609 -1.897773

TS-8
C -1.620624 0.731454 -0.227964
C -0.213892 0.848647 0.076412
C 0.116377 2.032656 -0.510430
C -1.982701 2.025469 -0.448669
C -2.704951 3.131487 -0.410872
C 0.775938 2.940100 -1.208500
C -3.871043 3.344185 0.466785
C -2.412828 -0.469535 -0.519663
C 0.634879 0.076802 0.995919
C -2.435256 -2.728297 -0.778469
C -3.799734 -2.774245 -1.059533
C -4.476699 -1.558677 -1.059799
C -3.782889 -0.386986 -0.778237
C 2.752129 -0.317863 1.728983
C 2.352342 -1.282030 2.652371
C 0.987885 -1.544120 2.724077
C 0.111394 -0.856068 1.891826
C -5.355532 2.646400 2.252160
C -6.123809 3.798267 2.076965
C -5.762154 4.725393 1.101670
C -4.643396 4.501311 0.304361
C 1.927169 2.666152 -2.086677
C 2.418921 1.368432 -2.290570
C 3.513138 1.153670 -3.119709
C 4.135148 2.226897 -3.760887
C 3.650897 3.518678 -3.567351
H -2.420593 3.958849 -1.060534
H 0.435574 3.974185 -1.156253
H -4.304724 -3.709394 -1.265212
H -5.540505 -1.530447 -1.270459
H -4.284634 0.575024 -0.753208
H 3.067461 -1.796714 3.281451
H 0.613054 -2.277660 3.430105
H -0.956676 -1.033621 1.933509
H -6.995181 3.972568 2.699817
H -6.350900 5.626416 0.961066
H -4.362994 5.225239 -0.455922
H 1.940520 0.532567 -1.788801
H 3.883147 0.143856 -3.268642
H 4.989449 2.054837 -4.407750
N -1.749039 -1.640140 -0.529595
I -1.327611 -4.574473 -0.744421
I 4.851679 0.139767 1.562873
N 1.953481 0.342597 0.928439
C -4.237996 2.420900 1.457133
H -3.634614 1.529166 1.608085
C 2.553705 3.735998 -2.738145
H 2.176510 4.744140 -2.588322
H 4.125901 4.359594 -4.062916
H -5.626025 1.923126 3.015048

2
C 0.730569 1.669175 -0.171191
C -0.730618 1.669139 0.171062
C -0.671366 0.186731 0.133188
C 0.671388 0.186767 -0.133343
C 1.596096 2.660043 -0.417097
C -1.596156 2.659991 0.416991
C 1.672780 -0.872157 -0.288275
C -1.672703 -0.872242 0.288082
C 1.391248 -2.058921 -0.966321
C 2.400446 -3.009636 -1.090672
C 3.652894 -2.753286 -0.544041
C 3.810971 -1.532587 0.111277
N 2.881525 -0.620659 0.249359
N -2.881520 -0.620695 -0.249368
C -3.810923 -1.532674 -0.111339
C -3.652731 -2.753479 0.543752
C -2.400213 -3.009879 1.090201
C -1.391060 -2.059112 0.965901
C 2.987039 2.541657 -0.879952
C -2.987083 2.541614 0.879887
C -3.940992 3.474467 0.450634
C -5.263527 3.388426 0.876045
C -5.651087 2.375580 1.752128
C -4.705585 1.456430 2.206465
C -3.384331 1.543727 1.780925
C 3.384298 1.543789 -1.781007
C 4.705568 1.456472 -2.206495
C 5.651073 2.375586 -1.752093
C 5.263501 3.388416 -0.875996
C 3.940952 3.474475 -0.450635
I 5.731955 -1.062958 0.961387
I -5.732017 -1.062940 -0.961138
H 1.229632 3.674953 -0.262661
H -1.229694 3.674902 0.262551
H 0.409802 -2.221431 -1.398450
H 2.217659 -3.939980 -1.617813
H 4.467453 -3.461685 -0.625969
H -4.467259 -3.461919 0.625652
H -2.217338 -3.940306 1.617166
H -0.409566 -2.221663 1.397904
H -3.639570 4.266487 -0.229799
H -5.990641 4.115008 0.527080
H -6.681075 2.309440 2.088338
H -4.995619 0.676228 2.903725
H -2.644399 0.846864 2.163946
H 2.644368 0.846951 -2.164078
H 4.995611 0.676284 -2.903766
H 6.681074 2.309432 -2.088261
H 5.990619 4.114970 -0.526981
H 3.639522 4.266482 0.229809

NEt_3
N -0.152024 -0.165216 -0.052018
C -0.044368 1.246574 0.311566
H -0.882095 1.779952 -0.146489
H -0.143240 1.376368 1.407820
C 1.249284 1.899643 -0.160107
H 1.372422 1.768511 -1.239555
H 1.223899 2.971161 0.057893
H 2.127285 1.480324 0.339788
C 0.934871 -0.965799 0.532780
H 0.513657 -1.898867 0.922744
H 1.374831 -0.448892 1.400853
C 2.016934 -1.313118 -0.485639
H 2.482629 -0.415463 -0.900010
H 2.799700 -1.924998 -0.025644
H 1.579440 -1.877294 -1.314594
C -1.462923 -0.706536 0.313822
H -1.442012 -1.777423 0.086241
H -1.647019 -0.615440 1.401915
C -2.618148 -0.075258 -0.456128
H -3.528935 -0.661083 -0.303293
H -2.829353 0.947685 -0.134706
H -2.390942 -0.061060 -1.526600

TS-1
C -1.606388 4.785145 -1.245840
C -0.496513 5.629999 -1.233184
C 0.654915 5.216273 -0.567494
C 0.704516 3.972399 0.057685
C -0.397367 3.098068 0.034828
C -1.561104 3.545771 -0.616266
H -2.521847 5.097543 -1.740228
H -0.532891 6.596897 -1.724427
H 1.526787 5.863176 -0.533578
H 1.620955 3.673008 0.560134
H -2.443827 2.913261 -0.613777
C -0.299077 1.731432 0.625345
C 0.597485 1.649884 1.909957
C 1.993559 1.344539 1.574149
C 3.143257 1.096114 1.290115
C -1.509857 0.995291 0.715367
C -2.494857 0.275733 0.740641
C 4.489457 0.797498 0.890892
C 5.527570 1.705591 1.110037
N 4.684414 -0.386278 0.272570
C 6.804270 1.373136 0.669130
H 5.326429 2.645567 1.609783
C 5.899054 -0.663773 -0.135045
C 7.012890 0.158685 0.024769
H 7.633241 2.055891 0.822164
H 7.989789 -0.135489 -0.337130
C -3.593800 -0.619317 0.847366

C -3.615345 -1.589933 1.863672
N -4.596683 -0.510124 -0.054758
C -4.695559 -2.456046 1.936227
H -2.792078 -1.640666 2.568860
C -5.598147 -1.348096 0.053280
C -5.732694 -2.348591 1.009819
H -4.738666 -3.215660 2.710215
H -6.593000 -3.004784 1.031940
I 6.149345 -2.551418 -1.139597
I -7.171689 -1.130669 -1.414009
C 0.058901 0.707543 2.982338
C 0.626742 -0.535614 3.255651
C -1.072032 1.106293 3.703555
C 0.063908 -1.376436 4.218039
H 1.517412 -0.849523 2.718845
C -1.631985 0.274602 4.665888
H -1.520183 2.074668 3.494223
C -1.065992 -0.975958 4.924427
H 0.517389 -2.342604 4.416386
H -2.509352 0.600022 5.216199
H -1.501383 -1.627575 5.675465
H 0.597636 2.654688 2.356989
N 0.621306 0.368960 -1.606497
C 0.005847 1.114329 -2.742936
H 0.257762 2.166937 -2.589557
H 0.494598 0.781682 -3.668632
C -1.503776 0.960324 -2.868898
H -2.006378 1.092091 -1.907423
H -1.872581 1.728626 -3.553500
H -1.782687 -0.014588 -3.276140
C 0.077085 -1.008931 -1.475218
H 0.315734 -1.565940 -2.391946
H -1.006676 -0.919526 -1.400510
C 0.588957 -1.729866 -0.236730
H 0.395601 -1.129907 0.657792
H 0.045643 -2.672597 -0.129692
H 1.655921 -1.961667 -0.283974
C 2.097866 0.323194 -1.771657
H 2.512323 -0.145397 -0.880150
H 2.314513 -0.330336 -2.628868
C 2.739963 1.687861 -1.969433
H 3.825657 1.575841 -1.889059
H 2.520113 2.115570 -2.950478
H 2.412588 2.391279 -1.198366
H 0.291936 0.989362 -0.522511

INT-1

C 0.489472 2.348460 3.392658
C 1.678241 1.697127 3.718786
C 2.235781 0.785232 2.824146
C 1.607406 0.527871 1.607822
C 0.420792 1.185684 1.271261
C -0.135838 2.094097 2.174618
H 0.048382 3.056908 4.086853
H 2.164941 1.895854 4.668464
H 3.158007 0.268717 3.072733
H 2.040520 -0.182321 0.907801
H -1.059991 2.605494 1.916896
C -0.204993 0.927814 -0.087626
H 0.099539 -0.077948 -0.416031
C 0.323851 1.929821 -1.187760
C 1.737500 1.621000 -1.410215
C 2.887335 1.261057 -1.506632
C -1.666821 0.952178 -0.052733
C -2.876206 0.947150 -0.047933
C 4.243279 0.792194 -1.568909
C 5.102576 1.171757 -2.600752
N 4.625765 -0.038713 -0.576841
C 6.399534 0.667463 -2.602381
H 4.753453 1.842682 -3.376436
C 5.852250 -0.497740 -0.612554
C 6.801203 -0.194031 -1.588096
H 7.094186 0.941066 -3.389346
H 7.800000 -0.610186 -1.554862
C -4.311742 0.947340 -0.037088
C -5.022729 2.148513 -0.081489
N -4.921333 -0.254741 0.016316
C -6.412637 2.097485 -0.069673
H -4.487338 3.090179 -0.122671
C -6.232288 -0.264105 0.026995
C -7.051161 0.863651 -0.013542
H -6.997134 3.010717 -0.103090
H -8.130545 0.779890 -0.001403
I 6.407938 -1.829719 0.984851
I -7.164851 -2.203470 0.116372
C 0.060927 3.378440 -0.823249
C 0.987705 4.125861 -0.093722
C -1.164960 3.956401 -1.163225
C 0.691753 5.430265 0.294203
H 1.941576 3.679033 0.174412
C -1.464163 5.259364 -0.771389
H -1.887795 3.380600 -1.735392
C -0.535929 5.998871 -0.040393
H 1.420483 6.001450 0.860731
H -2.419405 5.697763 -1.043028
H -0.766510 7.015233 0.262751
H -0.228910 1.695870 -2.105937
N 0.186605 -2.360932 -0.846082
C 1.134299 -3.348964 -0.326383
H 1.035981 -3.364573 0.762318
H 0.878378 -4.364706 -0.683244
C 2.585628 -3.020966 -0.662951
H 2.845629 -2.015196 -0.317152
H 3.251501 -3.735939 -0.170231
H 2.791570 -3.069562 -1.736221
C 0.058906 -2.452495 -2.305443
H -0.673295 -3.234281 -2.575856
H 1.016990 -2.770737 -2.723397
C -0.322708 -1.121261 -2.943888
H 0.496635 -0.405247 -2.821738
H -0.510807 -1.247486 -4.014136
H -1.225455 -0.693485 -2.492552
C -1.139983 -2.510779 -0.237656
H -1.811310 -1.808285 -0.743254
H -1.539283 -3.524836 -0.432297
C -1.187114 -2.220481 1.258911
H -2.229494 -2.115882 1.575054
H -0.740190 -3.019813 1.855897
H -0.666175 -1.286961 1.493453

TS-R_a-2

C 1.412335 5.323246 2.003439
C 0.242347 6.022828 1.723512
C -0.914412 5.306471 1.406623
C -0.899347 3.918839 1.380096
C 0.275084 3.194821 1.664203
C 1.432252 3.929606 1.971895
H 2.324892 5.862035 2.241395
H 0.229011 7.107638 1.748452
H -1.837340 5.834791 1.185506
H -1.805629 3.369462 1.139159
H 2.369784 3.416172 2.163160
C 0.281260 1.725039 1.608352
C 1.465932 0.946364 2.203460
C 2.497237 0.800008 1.166703
C 3.262488 0.688914 0.234847
C -0.658148 1.034374 0.954703
C -1.472262 0.383210 0.207858
C 4.083427 0.605324 -0.940395
C 4.825597 -0.541920 -1.262324
N 4.073419 1.682462 -1.746892
C 5.572028 -0.569457 -2.434351
C 4.791049 1.647868 -2.866428
C 5.557008 0.551021 -3.256111
H 6.151381 -1.446108 -2.702272
H 6.123788 0.571831 -4.179540
C -2.632216 -0.406387 0.584935
C -2.548039 -1.378900 1.598823
N -3.774406 -0.243794 -0.122743
C -3.654321 -2.164419 1.880256
H -1.615763 -1.494600 2.142090
C -4.799135 -1.001657 0.179255
C -4.835998 -1.985955 1.157814
H -3.607531 -2.915687 2.663268
H -5.722178 -2.578717 1.343239
C 1.027053 -0.387094 2.797902
C 1.319162 -1.610589 2.199363
C 0.268368 -0.370678 3.972910

C 0.854077 -2.802373 2.759818
H 1.920348 -1.633645 1.295587
C -0.189114 -1.556064 4.538025
H 0.029824 0.582893 4.437720
C 0.100381 -2.779735 3.929513
H 1.085928 -3.747344 2.277606
H -0.774347 -1.527223 5.451890
H -0.259058 -3.705600 4.367321
H 1.884412 1.542931 3.024712
H -0.928135 0.394263 -1.311319
N -0.418595 0.375186 -2.339766
C 0.821940 1.191074 -2.194641
H 1.340702 0.792497 -1.322324
H 1.446367 1.020716 -3.080547
C 0.563872 2.671624 -1.966580
H 0.233190 3.188586 -2.870362
H -0.174032 2.822900 -1.172174
H 1.505970 3.121220 -1.640056
C -1.366683 0.943990 -3.336500
H -0.835971 1.030253 -4.291972
H -1.609857 1.949685 -2.989548
C -2.648211 0.134771 -3.473626
H -3.100204 -0.044917 -2.491495
H -3.357639 0.711464 -4.072600
H -2.487797 -0.820244 -3.978569
C -0.108223 -1.058691 -2.605769
H -1.052557 -1.599767 -2.518963
H 0.243036 -1.141314 -3.640820
C 0.898366 -1.642003 -1.622949
H 0.920969 -2.727449 -1.748026
H 1.911720 -1.264614 -1.787398
H 0.602534 -1.419112 -0.592499
H 4.751555 2.540268 -3.485086
I 4.812138 -2.247888 0.003082
I -6.586123 -0.677981 -1.005488

TS-S_a-2
C -6.117283 -0.349431 3.067809
C -6.558864 0.957629 2.889497
C -5.736733 1.859925 2.210700
C -4.503706 1.456573 1.718706
C -4.036760 0.137965 1.892814
C -4.875883 -0.754837 2.578955
H -6.739187 -1.067364 3.594334
H -7.526104 1.270342 3.269194
H -6.063827 2.884137 2.056584
H -3.875760 2.164168 1.183000
H -4.567662 -1.780854 2.751398
C -2.723773 -0.249097 1.345898
C -2.342263 -1.731146 1.168809
C -0.901651 -1.907745 1.396376
C 0.290526 -1.989677 1.583431
C -1.871146 0.653419 0.857692
C -1.066421 1.446700 0.253843
C 1.696300 -2.038170 1.877459
C 2.673709 -2.182269 0.880351
N 2.034971 -1.921671 3.174231
C 4.017588 -2.203616 1.232603
C 3.323508 -1.945048 3.503003
C 4.353531 -2.083693 2.575370
H 4.787117 -2.307273 0.475201
H 5.389339 -2.095105 2.894146
C 0.125735 2.126951 0.738638
C 0.068454 3.233272 1.602696
N 1.311606 1.685079 0.258267
C 1.245826 3.866321 1.973427
H -0.893767 3.569511 1.973338
C 2.401072 2.302553 0.641009
C 2.468824 3.400552 1.489209
H 1.221735 4.719193 2.644807
H 3.409762 3.864175 1.755399
C -2.779598 -2.214346 -0.208645
C -1.923301 -2.117209 -1.307511
C -4.082492 -2.680686 -0.401576
C -2.358402 -2.494634 -2.577022
H -0.911543 -1.746733 -1.161415
C -4.522583 -3.042664 -1.672929
H -4.757556 -2.756608 0.446895
C -3.660779 -2.954394 -2.765360
H -1.676761 -2.427060 -3.421145
H -5.537532 -3.403451 -1.808114
H -3.999637 -3.247388 -3.753942
H -2.861685 -2.330991 1.923620
H -1.472792 1.735412 -1.213824
N -1.774577 2.038500 -2.311366
C -3.254687 2.194485 -2.324916
H -3.493660 2.916745 -1.541490
H -3.542962 2.629400 -3.289540
C -4.003618 0.897015 -2.048192
H -3.966431 0.198271 -2.887430
H -3.607622 0.395322 -1.157806
H -5.052797 1.139845 -1.860070
C -1.308294 0.946694 -3.205592
H -1.618958 1.186692 -4.229924
H -1.837758 0.047465 -2.890381
C 0.191785 0.701689 -3.109548
H 0.511415 0.640642 -2.062596
H 0.419802 -0.253353 -3.591798
H 0.777241 1.475143 -3.612355
C -1.057244 3.316818 -2.572044
H -0.001502 3.118293 -2.372906
H -1.165424 3.559061 -3.635940
C -1.526987 4.465910 -1.690600
H -0.819677 5.292351 -1.792211
H -2.514651 4.834108 -1.975247
H -1.549365 4.168700 -0.636945
H 3.546414 -1.847747 4.562252
I 2.144762 -2.366069 -1.166594
I 4.253755 1.511102 -0.155088

INT-R_a-2
C 1.568524 5.944541 -1.057850
C 0.709661 6.302846 -2.091654
C -0.387231 5.490528 -2.384937
C -0.617565 4.335649 -1.650294
C 0.244247 3.961686 -0.607843
C 1.339767 4.783799 -0.319681
H 2.425444 6.566708 -0.819812
H 0.889535 7.206836 -2.664407
H -1.067622 5.761445 -3.186067
H -1.477403 3.711082 -1.876312
H 2.033768 4.521439 0.471824
C -0.028844 2.724546 0.170946
C 0.813521 2.422613 1.426760
C 2.162079 2.023038 1.008007
C 3.245280 1.636715 0.636866
C -0.956094 1.864955 -0.183462
C -1.864921 0.979106 -0.508738
C 4.520973 1.153453 0.188505
C 5.491437 2.028172 -0.303455
N 4.717780 -0.178899 0.262355
C 6.703837 1.498827 -0.734026
H 5.289243 3.091879 -0.344832
C 5.869128 -0.643083 -0.157556
C 6.914307 0.126206 -0.667102
H 7.480608 2.149193 -1.121787
H 7.842007 -0.325743 -0.994408
C -3.262685 1.016531 -0.041128
C -3.788794 2.065464 0.718415
N -4.006759 -0.051814 -0.382812
C -5.107785 1.986440 1.145793
H -3.165998 2.920182 0.960784
C -5.244819 -0.100573 0.042865
C -5.874951 0.871740 0.812468
H -5.542700 2.784526 1.738552
H -6.904302 0.768473 1.131233
I 6.124907 -2.777968 -0.042483
I -6.358119 -1.862854 -0.514610
C 0.154576 1.378182 2.322319
C 0.663147 0.089547 2.472758
C -1.022182 1.729727 2.990355
C 0.005350 -0.834621 3.285709
H 1.577797 -0.192552 1.959088
C -1.685069 0.805356 3.790634
H -1.420125 2.735540 2.877202
C -1.169980 -0.482666 3.942437
H 0.413428 -1.834992 3.396628
H -2.600561 1.091511 4.299076

H -1.683912 -1.205793 4.567831
H 0.889786 3.358548 1.997329
H -1.585229 0.093962 -1.089235
N -0.935173 -2.071716 -1.169667
C 0.355574 -2.198336 -0.492118
H 0.187856 -2.062352 0.579558
H 0.774536 -3.214874 -0.625188
C 1.366549 -1.143698 -0.933128
H 0.954820 -0.140674 -0.772607
H 2.285237 -1.225543 -0.342147
H 1.645960 -1.237119 -1.986551
C -0.843624 -2.513834 -2.559700
H -0.645245 -3.602850 -2.607473
H 0.019081 -2.018545 -3.013494
C -2.079226 -2.179761 -3.390663
H -2.342131 -1.123414 -3.273147
H -1.875246 -2.367614 -4.448601
H -2.947458 -2.781802 -3.110681
C -2.013076 -2.778541 -0.473496
H -2.943899 -2.539990 -0.994143
H -1.870312 -3.875614 -0.541739
C -2.180974 -2.369386 0.987172
H -3.187552 -2.632997 1.327756
H -1.464688 -2.861692 1.650842
H -2.056300 -1.287599 1.100612

INT-S_{a}-2
C 4.639828 4.502048 2.197494
C 5.316759 3.840760 3.218911
C 5.151829 2.464434 3.374153
C 4.322216 1.758733 2.511109
C 3.631744 2.415017 1.481695
C 3.803403 3.796887 1.334858
H 4.757005 5.573439 2.070077
H 5.970825 4.391756 3.886828
H 5.679600 1.937261 4.162567
H 4.205672 0.684364 2.622321
H 3.272735 4.340307 0.559660
C 2.737946 1.632886 0.591657
C 2.508076 2.060643 -0.880451
C 1.105344 1.799342 -1.226454
C -0.060379 1.515878 -1.376951
C 2.141901 0.525158 0.966939
C 1.426206 -0.558270 1.146402
C -1.440362 1.119879 -1.459626
C -1.787081 -0.152001 -1.921994
N -2.359400 2.005915 -1.024034
C -3.130669 -0.513780 -1.929492
H -1.014949 -0.833043 -2.260533
C -3.613391 1.625623 -1.035521
C -4.084313 0.388231 -1.472417
H -3.433002 -1.494782 -2.281013
H -5.138870 0.143409 -1.450551
C -0.024810 -0.511260 1.410233
C -0.687074 0.650091 1.818213
N -0.690358 -1.651356 1.146805
C -2.071603 0.631686 1.918897
H -0.117521 1.550221 2.023113
C -1.997777 -1.633002 1.245538
C -2.768652 -0.537331 1.618381
H -2.614421 1.523808 2.216390
H -3.849220 -0.589465 1.669258
I -5.040749 3.046960 -0.274477
I -3.003333 -3.474150 0.745028
C 3.508078 1.356251 -1.794663
C 3.108919 0.430184 -2.756184
C 4.871027 1.630234 -1.634843
C 4.059533 -0.219883 -3.545767
H 2.052956 0.218368 -2.894546
C 5.817742 0.978234 -2.417493
H 5.189829 2.355832 -0.890521
C 5.414584 0.046890 -3.375927
H 3.733137 -0.937832 -4.292539
H 6.871852 1.198684 -2.281558
H 6.153012 -0.461406 -3.987559
H 2.682685 3.139299 -0.955036
H 1.858783 -1.542551 0.937037
N 2.762150 -3.248270 -0.215288
C 4.205449 -3.231191 0.029577
H 4.392602 -3.695578 1.002843
H 4.733863 -3.850460 -0.718721
C 4.770929 -1.814480 0.043746
H 4.326865 -1.238285 0.862316
H 5.855979 -1.836796 0.185537
H 4.566216 -1.278140 -0.889219
C 2.464113 -3.055903 -1.636561
H 2.535608 -4.015455 -2.180464
H 3.229306 -2.400313 -2.062189
C 1.101723 -2.410785 -1.861776
H 1.081464 -1.417695 -1.399030
H 0.905203 -2.303340 -2.934727
H 0.288223 -2.998431 -1.422875
C 2.137571 -4.470162 0.295572
H 1.174274 -4.592888 -0.210484
H 2.744550 -5.356129 0.032922
C 1.887936 -4.413708 1.798190
H 1.460897 -5.357920 2.149767
H 2.810233 -4.235576 2.359690
H 1.183759 -3.607627 2.026950

TS-R_{a}-3
C 0.805542 2.994231 2.538418
C 1.955525 3.487698 1.926131
C 2.331872 3.005389 0.673305
C 1.574375 2.022082 0.042043
C 0.431008 1.503225 0.660970
C 0.045176 2.013307 1.904579
H 0.496178 3.372707 3.507653
H 2.550523 4.250638 2.418080
H 3.217695 3.396782 0.182314
H 1.873367 1.649738 -0.931604
H -0.855094 1.633541 2.381546
C -0.384482 0.427247 0.003605
C 0.198741 -0.959137 -0.134521
C 1.549333 -0.979120 -0.599096
C 2.737129 -1.034964 -0.866296
C -1.593722 0.752842 -0.397087
C -2.796448 1.074445 -0.820335
C 4.138708 -1.081421 -1.116147
C 4.764480 -2.270179 -1.519753
N 4.836306 0.063435 -0.929885
C 6.135670 -2.264897 -1.732896
H 4.172563 -3.168068 -1.655403
C 6.128514 0.024046 -1.140430
C 6.859852 -1.089876 -1.542295
H 6.645606 -3.170573 -2.044829
H 7.930206 -1.044174 -1.696102
C -4.032702 0.918684 -0.033572
C -4.032305 0.629582 1.334912
N -5.182701 1.064530 -0.720464
C -5.243614 0.462341 1.990993
H -3.090972 0.551728 1.866699
C -6.311107 0.906411 -0.069974
C -6.434389 0.598234 1.280513
H -5.267782 0.233855 3.051653
H -7.400946 0.478581 1.752877
I 7.192624 1.877124 -0.821874
I -8.116012 1.154512 -1.227904
C -0.698429 -2.008983 -0.723392
C -0.270108 -2.852476 -1.758001
C -1.990493 -2.218314 -0.210643
C -1.088017 -3.868607 -2.250359
H 0.721856 -2.710882 -2.177530
C -2.810137 -3.227192 -0.704530
H -2.362252 -1.582923 0.588623
C -2.363750 -4.062764 -1.728906
H -0.723918 -4.505479 -3.051196
H -3.802005 -3.362409 -0.283167
H -3.002596 -4.851534 -2.112960
H 0.536632 -1.419908 1.213825
H -2.943126 1.443082 -1.834655
N 0.886129 -1.930639 2.362666
C 1.403241 -0.862912 3.257803
H 0.629649 -0.097127 3.321043
H 1.533443 -1.293904 4.260648
C 2.692856 -0.216084 2.771062
H 2.623505 0.052375 1.712358
H 2.853877 0.703997 3.339549

H	3.563612	-0.860191	2.915819
C	1.931624	-2.960770	2.122617
H	2.282451	-3.320025	3.100486
H	2.761668	-2.453545	1.628883
C	1.475330	-4.126292	1.257688
H	1.031229	-3.776496	0.321253
H	2.349944	-4.733810	1.010360
H	0.757307	-4.771980	1.768569
C	-0.356802	-2.532238	2.907734
H	-0.737266	-3.212528	2.142904
H	-0.105065	-3.123436	3.798635
C	-1.418089	-1.485722	3.222864
H	-2.396779	-1.966762	3.298103
H	-1.224154	-0.959762	4.160285
H	-1.464224	-0.746393	2.415243

TS-S_a-3

C	-0.840897	-1.801564	3.532395
C	-1.727842	-2.660288	2.886361
C	-1.865958	-2.595345	1.500520
C	-1.139189	-1.663158	0.764206
C	-0.266438	-0.778688	1.408253
C	-0.109285	-0.873114	2.795150
H	-0.714934	-1.854138	4.609289
H	-2.300921	-3.383519	3.457878
H	-2.540139	-3.274759	0.988018
H	-1.250757	-1.619306	-0.313459
H	0.591466	-0.208719	3.294957
C	0.516994	0.245920	0.639024
C	-0.207490	1.341446	-0.096856
C	-1.457659	0.968514	-0.678294
C	-2.579882	0.736571	-1.092634
C	1.826850	0.159208	0.733042
C	3.130383	0.032833	0.844728
C	-3.906220	0.445065	-1.523186
C	-4.522816	1.190389	-2.538853
N	-4.543898	-0.574059	-0.900922
C	-5.820049	0.867928	-2.911637
H	-3.981475	2.000636	-3.013300
C	-5.766911	-0.844894	-1.283061
C	-6.481288	-0.180860	-2.275943
H	-6.320745	1.427927	-3.694758
H	-7.492653	-0.464195	-2.537256
C	3.957757	-0.760601	-0.082264
C	3.413377	-1.458174	-1.166541
N	5.279885	-0.773006	0.174257
C	4.260695	-2.178881	-1.994629
H	2.343416	-1.423650	-1.341483
C	6.053080	-1.465019	-0.629480
C	5.630331	-2.195014	-1.733661
H	3.865510	-2.728721	-2.842688
H	6.326305	-2.743998	-2.354655
I	-6.738320	-2.476823	-0.252008
I	8.162790	-1.446484	-0.168458
C	0.628420	2.262146	-0.940557
C	0.318172	2.512343	-2.284751
C	1.718935	2.953305	-0.386841
C	1.058647	3.417872	-3.042307
H	-0.520940	1.992771	-2.738014
C	2.462862	3.853500	-1.143210
H	1.986542	2.790304	0.652600
C	2.137219	4.093337	-2.477715
H	0.791058	3.589880	-4.080697
H	3.297243	4.374894	-0.683690
H	2.715213	4.798444	-3.066409
H	-0.764637	2.151792	0.965817
H	3.678779	0.536200	1.640801
N	-1.325238	2.926015	1.864663
C	-1.877878	2.098856	2.968585
H	-1.052594	1.505188	3.363268
H	-2.214317	2.777443	3.765513
C	-2.999932	1.158463	2.549920
H	-2.724138	0.581276	1.662222
H	-3.178464	0.453490	3.366654
H	-3.935667	1.686481	2.350570
C	-2.407711	3.677600	1.176916
H	-2.953407	4.259135	1.933525
H	-3.089325	2.932339	0.763989
C	-1.921506	4.581635	0.052922
H	-1.297864	4.031415	-0.658148
H	-2.794045	4.960078	-0.485945
H	-1.360108	5.443527	0.420672
C	-0.262379	3.835396	2.362434
H	0.174255	4.317790	1.485606
H	-0.728769	4.614460	2.981346
C	0.832287	3.113544	3.139594
H	1.695528	3.776911	3.236329
H	0.512287	2.832424	4.145407
H	1.158077	2.207160	2.614992

INT-S_a-3

C	-0.517109	-3.225189	1.167110
C	-1.545469	-3.728119	0.373339
C	-1.856637	-3.100974	-0.832968
C	-1.152964	-1.969887	-1.239118
C	-0.128711	-1.448420	-0.440639
C	0.189303	-2.094735	0.758611
H	-0.261336	-3.710656	2.103900
H	-2.098525	-4.607122	0.689038
H	-2.647955	-3.495378	-1.463045
H	-1.398478	-1.485353	-2.178363
H	0.999087	-1.707722	1.372854
C	0.637549	-0.220635	-0.853603
C	-0.084933	1.050052	-0.990248
C	-1.465773	1.013836	-1.096106
C	-2.699482	1.031161	-1.106109
C	1.935324	-0.415718	-1.034293
C	3.207036	-0.681493	-1.239659
C	-4.104225	1.044314	-1.026659
C	-4.840412	2.244137	-1.159799
N	-4.735996	-0.136344	-0.778937
C	-6.216121	2.208839	-1.020842
H	-4.317230	3.170583	-1.369735
C	-6.037283	-0.114326	-0.658181
C	-6.864373	0.997789	-0.757755
H	-6.797072	3.120844	-1.117233
H	-7.937603	0.930030	-0.640403
C	4.263938	-0.580260	-0.215700
C	4.000932	-0.250055	1.118660
N	5.519897	-0.817888	-0.644339
C	5.056934	-0.155797	2.013738
H	2.978349	-0.076083	1.434391
C	6.495048	-0.724762	0.227905
C	6.356275	-0.397089	1.572175
H	4.876276	0.101059	3.052631
H	7.209110	-0.336114	2.235889
I	-6.966314	-2.030998	-0.244055
I	8.481531	-1.121175	-0.521776
C	0.570574	2.364337	-1.007162
C	-0.140098	3.510630	-1.430983
C	1.885831	2.575333	-0.542093
C	0.417437	4.781265	-1.371741
H	-1.148158	3.387655	-1.817342
C	2.439571	3.851435	-0.484268
H	2.480600	1.738001	-0.196073
C	1.715819	4.968481	-0.895542
H	-0.167113	5.632745	-1.708977
H	3.452205	3.968398	-0.107964
H	2.153171	5.960514	-0.851338
H	-1.424460	1.221700	0.992023
H	3.567663	-0.973407	-2.226854
N	-1.604625	1.444243	1.987206
C	-1.787886	0.132785	2.701372
H	-0.833480	-0.389204	2.639849
H	-1.991818	0.373587	3.748392
C	-2.868888	-0.729037	2.071155
H	-2.691555	-0.855230	0.997044
H	-2.823395	-1.717813	2.534244
H	-3.874995	-0.331761	2.221942
C	-2.860839	2.271508	2.032819
H	-3.110111	2.393864	3.090529
H	-3.637044	1.672270	1.556616
C	-2.724846	3.606990	1.322817
H	-2.305209	3.473707	0.320226
H	-3.723031	4.039350	1.219728
H	-2.106332	4.315534	1.877007
C	-0.408781	2.218569	2.472757
H	-0.291132	3.051952	1.777335

H -0.664804 2.611402 3.460587
C 0.864182 1.390662 2.493275
H 1.714506 2.070541 2.588690
H 0.897410 0.684075 3.325248
H 0.975458 0.849358 1.547055

INT-R_a-3

C -0.621834 -2.602861 2.419951
C -1.375877 -3.368083 1.533161
C -1.428696 -3.011469 0.185774
C -0.746443 -1.886832 -0.271181
C -0.004090 -1.100134 0.616631
C 0.063919 -1.479330 1.961461
H -0.565581 -2.877553 3.468788
H -1.913413 -4.242238 1.886829
H -2.000810 -3.612915 -0.513998
H -0.791702 -1.613157 -1.320080
H 0.663322 -0.886570 2.648471
C 0.729008 0.129310 0.150253
C -0.031015 1.210020 -0.484790
C -1.346816 0.968414 -0.854170
C -2.542318 0.821576 -1.116162
C 2.029289 0.105481 0.409054
C 3.311680 -0.024203 0.669421
C -3.930931 0.681307 -1.297299
C -4.712692 1.726365 -1.840391
N -4.507799 -0.485931 -0.898838
C -6.080317 1.554062 -1.955360
H -4.229126 2.643804 -2.157764
C -5.803775 -0.596909 -1.028724
C -6.674209 0.357924 -1.540135
H -6.696865 2.346785 -2.367657
H -7.739988 0.186552 -1.611258
C 4.286056 -0.607079 -0.273338
C 3.909976 -1.129352 -1.516521
N 5.570233 -0.613622 0.134708
C 4.885906 -1.660699 -2.345857
H 2.865306 -1.110663 -1.807886
C 6.467828 -1.124090 -0.675118
C 6.216834 -1.666893 -1.929185
H 4.621393 -2.071913 -3.314778
H 7.009562 -2.071527 -2.545117
I -6.649320 -2.477250 -0.353127
I 8.509506 -1.103378 0.033309
C 0.521996 2.543224 -0.768818
C -0.118361 3.396953 -1.695196
C 1.654597 3.063769 -0.108892
C 0.332978 4.688649 -1.933041
H -0.985211 3.026093 -2.235228
C 2.103231 4.359816 -0.351623
H 2.183245 2.459424 0.617574
C 1.450969 5.186985 -1.262818
H -0.190303 5.308910 -2.655404
H 2.974247 4.724554 0.185800
H 1.806042 6.195183 -1.449743
H -1.566149 1.654582 1.144487
H 3.742144 0.314698 1.613241
N -2.003778 2.063934 1.990981
C -2.293291 0.927943 2.933441
H -1.329991 0.489080 3.191347
H -2.729050 1.371704 3.833131
C -3.181867 -0.138399 2.314218
H -2.786914 -0.465828 1.345753
H -3.185254 -1.003081 2.982488
H -4.214955 0.191011 2.183897
C -3.270414 2.749545 1.555268
H -3.767248 3.085790 2.469847
H -3.884539 1.982940 1.083312
C -3.032944 3.892663 0.584279
H -2.395576 3.573079 -0.246677
H -4.000464 4.192976 0.174294
H -2.586173 4.766249 1.062917
C -1.003614 3.045166 2.541542
H -0.758925 3.714122 1.714458
H -1.517886 3.615666 3.320064
C 0.264419 2.388161 3.059175
H 1.009299 3.171671 3.217942
H 0.112721 1.871379 4.008869
H 0.673279 1.685770 2.324411

TS-S_aS_a-4

C 1.525781 -2.813086 -1.556205
C 1.389425 -3.881927 -0.670023
C 0.526916 -3.767583 0.417955
C -0.188921 -2.590187 0.622112
C -0.057826 -1.512978 -0.260639
C 0.809287 -1.637407 -1.354010
H 2.191869 -2.894688 -2.409811
H 1.953519 -4.795971 -0.826533
H 0.417542 -4.591197 1.116786
H -0.853265 -2.494589 1.476871
H 0.918311 -0.808105 -2.046234
C -0.850776 -0.259387 -0.052977
C -0.207658 1.055048 -0.302915
C 1.132132 1.113841 -0.228479
C 2.394106 1.124119 -0.005490
C -2.088346 -0.387070 0.389836
C -3.340144 -0.537148 0.758262
C 3.479998 0.933168 -0.959918
C 3.399756 1.309267 -2.309937
N 4.612724 0.379216 -0.467606
C 4.487901 1.094337 -3.144580
H 2.484993 1.759287 -2.680435
C 5.615059 0.180204 -1.284884
C 5.645691 0.506900 -2.635305
H 4.441695 1.375172 -4.192181
H 6.516108 0.320028 -3.250730
C -4.465122 -0.654938 -0.189679
C -4.275056 -0.849285 -1.562319
N -5.697160 -0.568445 0.347970
C -5.386357 -0.927669 -2.390366
H -3.268597 -0.945962 -1.956377
C -6.726061 -0.651651 -0.462150
C -6.662228 -0.824484 -1.840608
H -5.267497 -1.075223 -3.458831
H -7.555326 -0.882385 -2.449379
I 7.371132 -0.728036 -0.403334
I -8.673866 -0.504171 0.456050
C -0.996901 2.290840 -0.514116
C -0.462290 3.539278 -0.153780
C -2.274168 2.271154 -1.094429
C -1.172592 4.715704 -0.364922
H 0.526696 3.574834 0.296086
C -2.987287 3.448929 -1.301363
H -2.709843 1.326988 -1.403930
C -2.444373 4.679237 -0.936797
H -0.733765 5.665458 -0.073414
H -3.972425 3.401484 -1.756510
H -3.002722 5.596182 -1.096051
H 2.751452 0.802459 1.485561
H -3.618931 -0.556380 1.811305
N 2.919600 0.415201 2.572623
C 3.514423 1.538543 3.343274
H 2.838925 2.387565 3.222321
H 3.528124 1.260014 4.403840
C 4.900831 1.922953 2.843920
H 4.906417 2.011345 1.752359
H 5.172974 2.890549 3.271801
H 5.663994 1.199139 3.137659
C 3.829970 -0.762916 2.492611
H 4.083086 -1.060667 3.517717
H 4.731873 -0.418851 1.986517
C 3.233855 -1.918529 1.702203
H 2.813918 -1.569794 0.751935
H 4.030018 -2.633933 1.478318
H 2.452204 -2.447460 2.251976
C 1.559575 0.034715 3.049242
H 1.152807 -0.638789 2.289350
H 1.672646 -0.527007 3.984187
C 0.616828 1.216759 3.224554
H -0.393947 0.828015 3.374633
H 0.872191 1.831434 4.090490
H 0.601122 1.843665 2.327373

TS-R_aS_a-4

C 2.180139 -0.031365 -3.551863
C 1.778537 -1.310370 -3.935733
C 0.561105 -1.813581 -3.480688

C -0.238611 -1.049508 -2.635787
C 0.159533 0.231835 -2.236470
C 1.377038 0.735125 -2.712208
H 3.122973 0.373859 -3.906819
H 2.409650 -1.909809 -4.584161
H 0.238630 -2.808554 -3.771460
H -1.178659 -1.453039 -2.267808
H 1.696426 1.729493 -2.417162
C -0.697770 1.034955 -1.309562
C -0.058919 1.980247 -0.353614
C 1.156437 1.645226 0.109520
C 2.238559 1.160962 0.595884
C -2.003075 0.838470 -1.323759
C -3.304855 0.670385 -1.349036
C 3.608816 1.326238 0.130141
C 4.097656 2.505005 -0.453908
N 4.421173 0.257580 0.306367
C 5.421623 2.562094 -0.867940
H 3.431148 3.351793 -0.576959
C 5.659750 0.341836 -0.107222
C 6.248573 1.451373 -0.702170
H 5.815855 3.463899 -1.325994
H 7.285409 1.453182 -1.012676
C -4.008109 -0.381770 -0.589017
C -3.332453 -1.427485 0.050038
N -5.349864 -0.282870 -0.551880
C -4.064240 -2.369003 0.759366
H -2.251661 -1.492074 -0.023835
C -6.013662 -1.192322 0.123441
C -5.452824 -2.262476 0.811430
H -3.563454 -3.187780 1.265956
H -6.062470 -2.976269 1.350438
I 6.864129 -1.429254 0.201606
I -8.160714 -0.979588 0.144055
C -0.761519 3.181383 0.149197
C -0.396928 3.747491 1.382737
C -1.781824 3.810868 -0.581560
C -1.022985 4.891412 1.864599
H 0.392486 3.275931 1.962548
C -2.410602 4.954191 -0.096066
H -2.075791 3.409202 -1.545931
C -2.039298 5.502254 1.130233
H -0.720622 5.303263 2.823040
H -3.194011 5.421755 -0.685389
H -2.533539 6.391538 1.508071
H 1.975668 -0.145091 1.411194
H -3.947352 1.327390 -1.935765
N 1.661720 -1.175232 1.862998
C 1.978077 -1.131574 3.315184
H 1.482278 -0.244364 3.713072
H 1.534831 -2.014328 3.791314
C 3.473789 -1.037080 3.586314
H 3.933579 -0.273166 2.950252
H 3.624564 -0.755883 4.631068
H 3.987838 -1.985502 3.416456
C 2.428000 -2.237438 1.150190
H 2.216848 -3.192039 1.647728
H 3.482173 -1.992057 1.280917
C 2.121043 -2.302523 -0.338429
H 2.195432 -1.311653 -0.800292
H 2.858373 -2.954878 -0.814346
H 1.129801 -2.710800 -0.548232
C 0.199566 -1.251792 1.584135
H 0.092470 -1.055650 0.511992
H -0.136116 -2.276929 1.784626
C -0.621138 -0.233783 2.364450
H -1.627230 -0.195734 1.936763
H -0.715582 -0.496616 3.420356
H -0.188716 0.767516 2.277676

TS-S_aR_a-4
C 1.525781 -2.813086 -1.556205
C 1.389425 -3.881927 -0.670023
C 0.526916 -3.767583 0.417955
C -0.188921 -2.590187 0.622112
C -0.057826 -1.512978 -0.260639
C 0.809287 -1.637407 -1.354010
H 2.191869 -2.894688 -2.409811
H 1.953519 -4.795971 -0.826533
H 0.417542 -4.591197 1.116786
H -0.853265 -2.494589 1.476871
H 0.918311 -0.808105 -2.046234
C -0.850776 -0.259387 -0.052977
C -0.207658 1.055048 -0.302915
C 1.132132 1.113841 -0.228479
C 2.394106 1.124119 -0.005490
C -2.088346 -0.387070 0.389836
C -3.340144 -0.537148 0.758262
C 3.479998 0.933168 -0.959918
C 3.399756 1.309267 -2.309937
N 4.612724 0.379216 -0.467606
C 4.487901 1.094337 -3.144580
H 2.484993 1.759287 -2.680435
C 5.615059 0.180204 -1.284884
C 5.645691 0.506900 -2.635305
H 4.441695 1.375172 -4.192181
H 6.516108 0.320028 -3.250730
C -4.465122 -0.654938 -0.189679
C -4.275056 -0.849285 -1.562319
N -5.697160 -0.568445 0.347970
C -5.386357 -0.927669 -2.390366
H -3.268597 -0.945962 -1.956377
C -6.726061 -0.651651 -0.462150
C -6.662228 -0.824484 -1.840608
H -5.267497 -1.075223 -3.458831
H -7.555326 -0.882385 -2.449379
I 7.371132 -0.728036 -0.403334
I -8.673866 -0.504171 0.456050
C -0.996901 2.290840 -0.514116
C -0.462290 3.539278 -0.153780
C -2.274168 2.271154 -1.094429
C -1.172592 4.715704 -0.364922
H 0.526696 3.574834 0.296086
C -2.987287 3.448929 -1.301363
H -2.709843 1.326988 -1.403930
C -2.444373 4.679237 -0.936797
H -0.733765 5.665458 -0.073414
H -3.972425 3.401484 -1.756510
H -3.002722 5.596182 -1.096051
H 2.751452 0.802459 1.485561
H -3.618931 -0.556380 1.811305
N 2.919600 0.415201 2.572623
C 3.514423 1.538543 3.343274
H 2.838925 2.387565 3.222321
H 3.528124 1.260014 4.403840
C 4.900831 1.922953 2.843920
H 4.906417 2.011345 1.752359
H 5.172974 2.890549 3.271801
H 5.663994 1.199139 3.137659
C 3.829970 -0.762916 2.492611
H 4.083086 -1.060667 3.517717
H 4.731873 -0.418851 1.986517
C 3.233855 -1.918529 1.702203
H 2.813918 -1.569794 0.751935
H 4.030018 -2.633933 1.478318
H 2.452204 -2.447460 2.251976
C 1.559575 0.034715 3.049242
H 1.152807 -0.638789 2.289350
H 1.672646 -0.527007 3.984187
C 0.616828 1.216759 3.224554
H -0.393947 0.828015 3.374633
H 0.872191 1.831434 4.090490
H 0.601122 1.843665 2.327373

TS-S_aR_a-4*
C 0.011977 -3.592777 -0.303237
C -1.196079 -3.720778 -0.985427
C -1.749099 -2.610730 -1.624030
C -1.103151 -1.378393 -1.578528
C 0.110402 -1.239720 -0.892996
C 0.660482 -2.360147 -0.260295
H 0.450917 -4.449985 0.197518
H -1.705067 -4.679143 -1.020285
H -2.688668 -2.703758 -2.160583
H -1.536483 -0.521417 -2.083212
H 1.602623 -2.259070 0.271476
C 0.836027 0.071873 -0.842713
C 0.115762 1.297257 -0.436675

C -1.201011 1.253888 -0.163849
C -2.405064 1.143682 0.241120
C 2.120361 0.037164 -1.149492
C 3.406841 -0.064957 -1.394257
C -3.685598 1.420916 -0.375163
C -4.027698 2.697232 -0.857522
N -4.591792 0.414942 -0.394208
C -5.296899 2.911465 -1.372321
H -3.293805 3.494163 -0.817290
C -5.776735 0.654480 -0.896023
C -6.222285 1.866396 -1.406168
H -5.574951 3.889056 -1.754098
H -7.223182 1.993055 -1.797504
C 4.414369 -0.340847 -0.351523
C 4.076054 -0.560794 0.989399
N 5.695889 -0.362379 -0.766352
C 5.087292 -0.803125 1.908395
H 3.033668 -0.540793 1.292383
C 6.627288 -0.595785 0.127950
C 6.413781 -0.825524 1.482526
H 4.850953 -0.975914 2.953316
H 7.232529 -1.011742 2.165565
I -7.148952 -1.022159 -0.885727
I 8.659531 -0.615731 -0.600005
C 0.832882 2.584365 -0.245491
C 0.234068 3.788418 -0.645073
C 2.089622 2.650622 0.374503
C 0.861347 5.012009 -0.430839
H -0.732772 3.751254 -1.139775
C 2.722445 3.873310 0.581373
H 2.567646 1.739479 0.720523
C 2.113501 5.061117 0.179818
H 0.374931 5.928402 -0.751646
H 3.692640 3.896921 1.068915
H 2.608888 6.013356 0.340795
H -2.517892 0.192644 1.522392
H 3.809763 0.073567 -2.397002
N -2.616303 -0.492285 2.428702
C -2.467781 -1.907945 1.966777
H -1.604889 -1.918155 1.294474
H -2.227137 -2.514224 2.848029
C -3.684417 -2.459760 1.241768
H -3.989703 -1.794905 0.428248
H -3.409646 -3.428110 0.816363
H -4.532548 -2.615081 1.913523
C -3.960273 -0.207125 3.011708
H -4.128972 -0.910989 3.834717
H -4.682073 -0.414462 2.219828
C -4.115260 1.236589 3.468386
H -3.814997 1.929515 2.676015
H -5.169068 1.415720 3.694338
H -3.538980 1.456968 4.369203
C -1.509604 -0.099935 3.350569
H -1.582636 0.981705 3.478686
H -1.703250 -0.576879 4.318132
C -0.124310 -0.454009 2.827488
H 0.616296 -0.008749 3.496993
H 0.050609 -1.532084 2.801543
H 0.034619 -0.044688 1.824285

TS-S_aR_a-4**

C 0.247237 -3.643824 -0.961124
C -0.878699 -3.705590 -1.775394
C -1.400677 -2.535006 -2.322055
C -0.805622 -1.308607 -2.051316
C 0.324524 -1.237940 -1.230868
C 0.846219 -2.415940 -0.692919
H 0.660353 -4.548743 -0.531650
H -1.348306 -4.659593 -1.984467
H -2.275209 -2.577002 -2.960905
H -1.213947 -0.402134 -2.482836
H 1.721467 -2.366614 -0.054587
C 0.982911 0.076298 -0.934023
C 0.167870 1.184926 -0.387087
C -1.148119 1.017377 -0.212481
C -2.365563 0.767666 0.095750
C 2.275858 0.151568 -1.166378
C 3.572719 0.153820 -1.352445
C -3.549088 1.022908 -0.728012
C -3.519746 1.725010 -1.940050
N -4.713286 0.527926 -0.253507
C -4.694217 1.905022 -2.654871
H -2.577237 2.117209 -2.303213
C -5.801767 0.706174 -0.954674
C -5.887641 1.383454 -2.163465
H -4.689171 2.444694 -3.594557
H -6.824965 1.496219 -2.688780
C 4.546004 -0.228592 -0.311096
C 4.162140 -0.659593 0.962178
N 5.841577 -0.129173 -0.659668
C 5.143191 -0.986264 1.884978
H 3.110765 -0.735584 1.213274
C 6.744117 -0.448960 0.235381
C 6.483684 -0.883562 1.528270
H 4.872729 -1.321265 2.879042
H 7.280434 -1.128092 2.215622
I -7.593659 -0.157018 -0.118265
I 8.798813 -0.275525 -0.384354
C 0.789199 2.463419 0.047365
C 0.128749 3.677107 -0.184770
C 2.007618 2.498218 0.737393
C 0.664887 4.881510 0.254549
H -0.814146 3.666124 -0.720944
C 2.547667 3.704197 1.171445
H 2.528862 1.575148 0.959586
C 1.881878 4.902365 0.930661
H 0.135230 5.806955 0.060115
H 3.489358 3.704427 1.708355
H 2.305174 5.840997 1.267523
H -2.599527 -0.237703 1.232775
H 4.008749 0.467428 -2.297731
N -2.739244 -1.110443 2.031697
C -2.534575 -2.392264 1.300607
H -1.617556 -2.271129 0.719519
H -2.370932 -3.183119 2.040530
C -3.671630 -2.757229 0.358470
H -3.903580 -1.927931 -0.313311
H -3.350666 -3.606122 -0.246138
H -4.578826 -3.044938 0.890495
C -4.105048 -0.9800X5 2.605973
H -4.263027 -1.805464 3.308632
H -4.801033 -1.080301 1.776289
C -4.338172 0.372513 3.262592
H -4.048918 1.175781 2.580872
H -5.403406 0.476261 3.473845
H -3.798162 0.487819 4.202379
C -1.686527 -0.887165 3.060153
H -1.786419 0.148132 3.386861
H -1.908785 -1.536887 3.913156
C -0.267563 -1.118275 2.557613
H 0.426301 -0.786704 3.331682
H -0.064467 -2.1700X2 2.353137
H -0.068564 -0.539658 1.652487

TS-R_aR_a-4

C 0.636884 0.955307 -0.558288
C 1.182923 0.597557 0.777162
C 2.268213 1.167960 1.265368
C -0.682766 0.821347 -0.767393
C -1.958390 0.803161 -0.875129
C 3.372304 1.694444 1.744028
C -2.824161 -0.255035 -1.372873
C 1.497112 1.532470 -1.619030
C 0.483670 -0.417133 1.627570
C 2.861040 1.216338 -1.719606
C 3.644321 1.751234 -2.738952
C 3.089475 2.611754 -3.685047
C 1.734042 2.929230 -3.599253
C 0.950743 2.400141 -2.578807
C 0.519005 -0.314611 3.023856
C -0.081806 -1.281125 3.825535
C -0.744965 -2.360013 3.242974
C -0.793167 -2.466872 1.853996
C -0.182886 -1.506222 1.051769
N -4.011133 -0.404376 -0.737744
C -4.827940 -1.336573 -1.156117
C -4.611518 -2.194176 -2.227894
C -3.398516 -2.032880 -2.897495

C -2.498470 -1.065504 -2.472715
I -6.685827 -1.509292 -0.056725
C 4.722396 1.157092 1.491539
C 5.851446 1.807257 1.996078
C 7.110643 1.282537 1.725203
C 7.223324 0.128034 0.957101
C 6.027866 -0.430435 0.510734
N 4.829255 0.032635 0.756229
I 6.119483 -2.214684 -0.702309
H -2.658829 1.851789 0.096760
H 3.326758 2.594470 2.357446
H 3.311261 0.538713 -1.000017
H 4.697223 1.487492 -2.794011
H 3.703108 3.029052 -4.477203
H 1.285052 3.600268 -4.325857
H -0.103687 2.656622 -2.513101
H 1.023891 0.533487 3.478215
H -0.041658 -1.183809 4.906178
H -1.221380 -3.110381 3.865964
H -1.302327 -3.306105 1.389830
H -0.214385 -1.603934 -0.028647
H -5.340626 -2.936594 -2.525205
H -3.161484 -2.669952 -3.744002
H -1.545819 -0.923230 -2.971056
H 5.736762 2.709486 2.586818
H 8.002726 1.769181 2.105860
H 8.182760 -0.312303 0.717877
N -3.087303 2.520717 0.929585
C -4.571162 2.388704 0.894979
H -4.788404 1.331513 1.045091
H -4.982357 2.964686 1.732536
C -5.165785 2.818406 -0.437813
H -4.635578 2.333496 -1.264003
H -6.209131 2.494517 -0.472469
H -5.146155 3.900334 -0.584618
C -2.639743 3.909084 0.624761
H -3.154333 4.585737 1.316875
H -2.985775 4.127850 -0.387158
C -1.129409 4.091949 0.691382
H -0.609964 3.328552 0.102248
H -0.883438 5.071455 0.274102
H -0.751830 4.061969 1.715803
C -2.487632 1.996819 2.190997
H -1.407360 1.967249 2.023321
H -2.696084 2.717289 2.990762
C -2.965444 0.598575 2.550064
H -2.327901 0.212776 3.348965
H -3.999376 0.586004 2.902582
H -2.871057 -0.072692 1.690334

INT-S_aS_a-4

C 1.246576 1.560197 4.087701
C 2.122579 2.621419 4.306297
C 2.425037 3.495660 3.263281
C 1.861291 3.306993 2.004809
C 0.985480 2.239594 1.777739
C 0.679920 1.371268 2.829848
H 1.005323 0.875970 4.894853
H 2.566292 2.768578 5.285752
H 3.098530 4.330239 3.430248
H 2.089535 3.997100 1.198516
H -0.002610 0.544194 2.656082
C 0.352440 2.042763 0.438034
C 1.200198 2.007348 -0.789214
C 2.509009 2.105503 -0.713003
C 3.817947 2.157094 -0.663570
C -0.952659 1.890261 0.368997
C -2.250731 1.709841 0.334835
C 4.663354 0.963192 -0.473284
C 6.053684 1.076318 -0.438948
N 4.043123 -0.224034 -0.330246
C 6.816281 -0.072780 -0.251274
H 6.522535 2.047005 -0.556576
C 4.787978 -1.284666 -0.155049
C 6.181194 -1.300385 -0.102552
H 7.899318 -0.015047 -0.220017
H 6.734696 -2.218552 0.046526
C -2.897816 0.386008 0.420409
C -2.176469 -0.798952 0.597806
N -4.238632 0.390455 0.306930
C -2.867424 -2.001958 0.652588
H -1.095678 -0.768406 0.692985
C -4.862170 -0.763367 0.364986
C -4.255092 -2.003509 0.533796
H -2.333604 -2.936759 0.788388
H -4.831831 -2.918719 0.572209
I 3.735293 -3.155612 0.071832
I -7.008547 -0.693466 0.178958
C 0.569082 1.826132 -2.132057
C 0.872760 2.709068 -3.171770
C -0.302951 0.758871 -2.374744
C 0.309143 2.533676 -4.433263
H 1.549613 3.537135 -2.982767
C -0.864751 0.584273 -3.635878
H -0.530786 0.054468 -1.580062
C -0.562835 1.472584 -4.667293
H 0.549593 3.228654 -5.231493
H -1.535523 -0.250338 -3.813669
H -1.003978 1.336151 -5.649459
H 4.328993 3.114766 -0.749013
H -2.925675 2.555048 0.213463

INT-S_aR_a-4

C -2.499790 3.574782 -0.467938
C -1.687701 4.698743 -0.322569
C -0.361452 4.546634 0.077376
C 0.148308 3.276815 0.334832
C -0.663348 2.147042 0.196693
C -1.993634 2.303847 -0.210375
H -3.531563 3.685495 -0.787220
H -2.086629 5.688445 -0.520968
H 0.276947 5.416635 0.193514
H 1.181063 3.154817 0.649876
H -2.628445 1.430167 -0.333942
C -0.099953 0.784150 0.437495
C -0.874204 -0.208853 1.237180
C -1.976178 0.133568 1.866324
C -3.101944 0.421538 2.473401
C 1.067288 0.475431 -0.086115
C 2.243749 0.208441 -0.599164
C -4.415247 0.308458 1.812222
C -5.587613 0.638432 2.493178
N -4.432856 -0.111822 0.532123
C -6.804527 0.527730 1.827158
H -5.540319 0.975062 3.522869
C -5.592243 -0.200673 -0.068640
C -6.827113 0.097325 0.505078
H -7.732054 0.776519 2.332217
H -7.750869 -0.000379 -0.050737
C 3.517345 0.433088 0.112274
C 3.576438 0.972971 1.400989
N 4.626510 0.072297 -0.558463
C 4.817530 1.139762 2.000390
H 2.661847 1.254344 1.913227
C 5.784394 0.242741 0.036448
C 5.969526 0.768361 1.310997
H 4.894193 1.556177 2.999343
H 6.955941 0.881645 1.742309
I -5.543702 -0.862313 -2.120073
I 7.524557 -0.359424 -1.084411
C -0.418527 -1.629511 1.323448
C 0.873481 -1.952500 1.751157
C -1.309056 -2.655701 0.994047
C 1.267923 -3.284632 1.840605
H 1.566778 -1.163078 2.027456
C -0.912104 -3.987356 1.083815
H -2.310846 -2.395854 0.662083
C 0.378045 -4.304081 1.504860
H 2.270885 -3.526095 2.178054
H -1.609832 -4.776375 0.821788
H 0.689233 -5.341601 1.573383
H -3.094447 0.779318 3.501233
H 2.330876 -0.225812 -1.593466

INT-R_aR_a-4

C 0.481059 2.681780 0.460671
C -0.737773 2.644484 -0.399804
C -1.912362 2.360764 0.119308

C 1.674762 2.520589 -0.067374
C 2.874483 2.339601 -0.563207
C -3.091765 2.057111 0.603694
C 3.514787 1.012916 -0.650819
C 0.353578 2.836944 1.938703
C -0.626042 2.865593 -1.870774
C -0.538341 3.760102 2.495512
C -0.622715 3.907001 3.877141
C 0.177072 3.132114 4.716051
C 1.065081 2.208698 4.166714
C 1.152001 2.060325 2.785465
C 0.180528 3.884265 -2.390029
C 0.250065 4.090800 -3.764838
C -0.479598 3.281389 -4.634492
C -1.282314 2.263189 -4.122739
C -1.354097 2.054956 -2.748383
N 2.878628 -0.025207 -0.073856
C 3.441975 -1.203913 -0.145943
C 4.655986 -1.487063 -0.770684
C 5.310014 -0.411907 -1.361290
C 4.737967 0.855593 -1.303219
I 2.376111 -2.823173 0.799699
C -3.609771 0.676034 0.645622
C -4.829488 0.392989 1.260887
C -5.283238 -0.922640 1.279078
C -4.517278 -1.920071 0.686857
C -3.319337 -1.513110 0.100713
N -2.865786 -0.286366 0.066250
I -2.082015 -3.007845 -0.841025
H 3.444907 3.188531 -0.937438
H -3.736807 2.838547 1.002936
H -1.157576 4.370339 1.844999
H -1.312960 4.630677 4.299033
H 0.105897 3.245615 5.793092
H 1.685586 1.596921 4.813950
H 1.829916 1.332428 2.346887
H 0.744587 4.520989 -1.715204
H 0.873642 4.887960 -4.157072
H -0.419977 3.441765 -5.706262
H -1.847454 1.624542 -4.794212
H -1.964784 1.253633 -2.340128
H 5.065259 -2.489039 -0.790759
H 6.259934 -0.564032 -1.863136
H 5.225832 1.711926 -1.755563
H -5.405879 1.191123 1.715365
H -6.227480 -1.172186 1.751792
H -4.831043 -2.955955 0.678303

TS-*R*aRa-5

C 0.319783 -2.179446 -0.585110
C -0.317373 -2.110597 0.774674
C -1.568732 -2.451993 0.967619
C 1.572264 -2.534522 -0.741662
C 2.835996 -2.829592 -0.934797
C -2.832002 -2.726099 1.192316
C 3.906235 -1.820460 -0.822545
C -0.434287 -1.686778 -1.782005
C 0.432401 -1.488596 1.912682
C -1.696836 -2.181844 -2.123889
C -2.366766 -1.681118 -3.237605
C -1.784233 -0.687398 -4.022694
C -0.521763 -0.198111 -3.692386
C 0.149303 -0.694572 -2.577984
C 1.704633 -1.922903 2.298777
C 2.368003 -1.296769 3.351508
C 1.769838 -0.237051 4.031417
C 0.498109 0.192976 3.657157
C -0.166412 -0.428853 2.603523
N 3.539737 -0.555883 -0.536029
C 4.482971 0.341542 -0.414328
C 5.849945 0.108402 -0.560843
C 6.222817 -1.196519 -0.861867
C 5.243971 -2.177423 -0.993925
I 3.830067 2.341464 0.065234
C -3.902564 -1.732105 0.985874
C -5.240356 -2.072617 1.187659
C -6.219343 -1.107580 0.968741
C -5.846632 0.165758 0.553875
C -4.479650 0.385223 0.387819
N -3.536379 -0.497884 0.588209
I -3.827540 2.336987 -0.262085
H 3.136628 -3.851226 -1.158744
H -3.132318 -3.719885 1.518613
H -2.152645 -2.964288 -1.527493
H -3.344951 -2.074908 -3.495492
H -2.310730 -0.298569 -4.888494
H -0.060311 0.575604 -4.297914
H 1.128296 -0.310266 -2.303414
H 2.173685 -2.754508 1.785017
H 3.353873 -1.644087 3.644587
H 2.291745 0.249359 4.849365
H 0.024567 1.018035 4.179929
H -1.151815 -0.090787 2.293483
H 6.577764 0.901230 -0.445453
H 7.271176 -1.445370 -0.989716
H 5.506480 -3.204109 -1.224017
H -5.502832 -3.074723 1.508267
H -7.267683 -1.344259 1.117979
H -6.574273 0.945531 0.369351

TS-*S*a*S*a-5

C 1.131558 -2.960683 -4.045160
C 2.321368 -3.660618 -4.240158
C 3.163000 -3.905702 -3.156700
C 2.820675 -3.453191 -1.885514
C 1.625202 -2.754604 -1.682014
C 0.786331 -2.510021 -2.775209
H 0.474484 -2.756984 -4.884657
H 2.593045 -4.008891 -5.231458
H 4.089906 -4.452107 -3.299238
H 3.479498 -3.648683 -1.045097
H -0.135033 -1.953852 -2.624942
C 1.232386 -2.290519 -0.316272
C 2.310712 -1.843946 0.630551
C 3.284438 -1.120080 0.126204
C 4.216509 -0.363255 -0.399424
C -0.042344 -2.233596 -0.001612
C -1.331377 -2.125677 0.217786
C 4.079449 1.100744 -0.523656
C 5.129844 1.874712 -1.017482
N 2.905350 1.648342 -0.153564
C 4.955709 3.251452 -1.126183
H 6.061826 1.403167 -1.308828
C 2.773493 2.944684 -0.267975
C 3.746937 3.821943 -0.744462
H 5.755530 3.878349 -1.506416
H 3.567525 4.887192 -0.813937
C -2.106308 -0.912497 -0.115300
C -1.504934 0.257999 -0.589258
N -3.435547 -1.006116 0.069506
C -2.311919 1.345908 -0.893204
H -0.426538 0.306611 -0.705889
C -4.169211 0.042545 -0.223421
C -3.690334 1.252896 -0.713045
H -1.874143 2.266881 -1.264919
H -4.353825 2.078393 -0.936891
I 0.869409 3.760141 0.336360
I -6.291399 -0.161534 0.097672
C 2.324411 -2.198021 2.081075
C 3.540732 -2.563801 2.671871
C 1.172751 -2.160117 2.874247
C 3.605915 -2.884465 4.023862
H 4.435393 -2.605300 2.057280
C 1.240165 -2.484592 4.227341
H 0.224849 -1.860283 2.443811
C 2.453430 -2.848419 4.806848
H 4.556356 -3.170433 4.463120
H 0.338697 -2.445327 4.830634
H 2.500827 -3.102314 5.860935
H 5.134290 -0.811833 -0.776216
H -1.897401 -2.940848 0.665025

TS-*S*a*R*a-5

C 2.524111 -2.496847 -1.513354
C 1.758895 -3.636623 -1.750498
C 0.387271 -3.610172 -1.498423
C -0.211783 -2.453569 -1.011271
C 0.551131 -1.303812 -0.773037

C 1.926722 -1.335889 -1.028505
H 3.593205 -2.505600 -1.705249
H 2.227301 -4.541685 -2.123805
H -0.216252 -4.495570 -1.671371
H -1.278016 -2.439363 -0.801382
H 2.536076 -0.458505 -0.836067
C -0.101883 -0.057585 -0.268094
C 0.715583 0.896572 0.558846
C 1.459093 0.401194 1.521631
C 2.236388 -0.094532 2.454335
C -1.368683 0.168812 -0.535293
C -2.646479 0.317160 -0.794908
C 3.636534 -0.481749 2.196763
C 4.372587 -1.185474 3.150505
N 4.167149 -0.133781 1.008805
C 5.683729 -1.546495 2.853754
H 3.920669 -1.446487 4.101003
C 5.398121 -0.496222 0.754609
C 6.228950 -1.204106 1.621134
H 6.279130 -2.095072 3.576114
H 7.242442 -1.468519 1.348221
C -3.721437 -0.233653 0.055170
C -3.459772 -0.978234 1.209301
N -4.975706 0.024642 -0.356803
C -4.529899 -1.463692 1.947726
H -2.434490 -1.164221 1.511653
C -5.965606 -0.448424 0.364646
C -5.831601 -1.199987 1.526985
H -4.358884 -2.045127 2.847700
H -6.693614 -1.559438 2.074027
I 6.160712 0.069224 -1.180830
I -7.953969 -0.003985 -0.339778
C 0.777606 2.360172 0.266675
C -0.354010 3.112998 -0.061526
C 2.024855 2.996802 0.325674
C -0.238167 4.476927 -0.320929
H -1.328994 2.641033 -0.100790
C 2.136390 4.359330 0.068552
H 2.906645 2.404443 0.557315
C 1.003952 5.104404 -0.257042
H -1.125566 5.050457 -0.569506
H 3.110210 4.836810 0.112673
H 1.089661 6.166570 -0.462805
H 1.854702 -0.248672 3.461759
H -2.977299 0.871369 -1.671799

INT-*S*$_a$*S*$_a$-5

C 4.692585 2.649020 -2.617381
C 5.536199 3.123792 -1.612146
C 5.358354 2.689654 -0.300751
C 4.342781 1.787915 0.008148
C 3.492601 1.309997 -0.994564
C 3.681851 1.745356 -2.312446
H 4.827766 2.978704 -3.642647
H 6.328930 3.825072 -1.851948
H 6.009918 3.053925 0.487160
H 4.204001 1.457651 1.033464
H 3.032064 1.365600 -3.096188
C 2.403610 0.358713 -0.662047
C 2.403303 -0.360403 0.661599
C 1.377950 -0.107891 1.444666
C 0.296886 0.196312 2.121609
C 1.378253 0.106857 -1.445309
C 0.296983 -0.196620 -2.122271
C -1.014867 -0.419956 1.849115
C -2.160419 0.035336 2.502664
N -1.069468 -1.386336 0.911920
C -3.395934 -0.493348 2.140359
H -2.081861 0.804674 3.262765
C -2.243207 -1.870370 0.602435
C -3.460479 -1.471000 1.154435
H -4.305141 -0.144098 2.619078
H -4.399365 -1.906247 0.836589
C -1.014341 0.420441 -1.849395
C -2.160435 -0.034279 -2.502389
N -1.068001 1.386965 -0.912298
C -3.395493 0.495115 -2.139546
H -2.082690 -0.803750 -3.262441
C -2.241327 1.871677 -0.602314
C -3.459074 1.472882 -1.153683
H -4.305113 0.146298 -2.617804
H -4.397594 1.908646 -0.835454
I -2.263958 -3.439550 -0.880457
I -2.260365 3.440999 0.880428
C 3.491768 -1.312174 0.994339
C 3.680313 -1.747829 2.312238
C 4.342186 -1.790230 -0.008094
C 4.690583 -2.651904 2.617437
H 3.030314 -1.367960 3.095746
C 5.357305 -2.692393 0.301079
H 4.203940 -1.459731 -1.033405
C 5.534447 -3.126806 1.612463
H 4.825241 -2.981816 3.642698
H 6.009063 -3.056758 -0.486629
H 6.326811 -3.828425 1.852483
H 0.325642 0.986285 2.870312
H 0.325174 -0.986470 -2.871118

INT-*R*$_a$*R*$_a$-5

C -0.313775 -0.919881 0.681807
C 0.313571 -0.919329 -0.682702
C 1.216964 -1.818830 -0.985498
C -1.216893 -1.819777 0.984217
C -2.194553 -2.648665 1.262289
C 2.194659 -2.647575 -1.263799
C -3.605237 -2.301917 0.999635
C 0.073645 0.172689 1.615254
C -0.073985 0.173707 -1.615519
C 1.340788 0.759675 1.525042
C 1.698422 1.781232 2.402356
C 0.799330 2.226466 3.369287
C -0.466505 1.646367 3.457681
C -0.829526 0.628153 2.583130
C -1.340903 0.761059 -1.524394
C -1.698723 1.783025 -2.401158
C -0.800051 2.228300 -3.368454
C 0.465556 1.647842 -3.457761
C 0.828758 0.629222 -2.583767
N -3.860742 -1.070816 0.514826
C -5.106218 -0.750935 0.277208
C -6.210116 -1.576229 0.485921
C -5.942667 -2.844241 0.989576
C -4.627367 -3.217233 1.251378
I -5.437943 1.234095 -0.500054
C 3.605305 -2.301178 -1.000659
C 4.627309 -3.216599 -1.252543
C 5.942617 -2.843930 -0.990345
C 6.210160 -1.576139 -0.486167
C 5.106375 -0.750739 -0.277299
N 3.860877 -1.070313 -0.515313
I 5.438331 1.233912 0.500761
H -1.992814 -3.624370 1.699201
H 1.992898 -3.622963 -1.701444
H 2.048872 0.405334 0.777790
H 2.685366 2.228207 2.328511
H 1.080627 3.025068 4.048343
H -1.175106 1.994883 4.202437
H -1.821098 0.185459 2.632384
H -2.048668 0.406663 -0.776875
H -2.685493 2.230277 -2.326617
H -1.081497 3.027190 -4.047111
H 1.173835 1.996392 -4.202807
H 1.820149 0.186218 -2.633794
H -7.217452 -1.245175 0.268415
H -6.757352 -3.536280 1.175838
H -4.391342 -4.200064 1.643987
H 4.391169 -4.199242 -1.645554
H 6.757235 -3.536017 -1.176713
H 7.217515 -1.245318 -0.268394

INT-*S*$_a$*R*$_a$-5

C 2.246013 -0.943755 1.158938
C 1.999938 -1.824403 -0.030951
C 1.285703 -2.917790 0.101997
C 1.216122 -0.615737 1.904953
C 0.158298 -0.256225 2.589739
C 0.469922 -3.935169 0.237434

C -0.670017 0.905577 2.212650
C 3.617034 -0.430251 1.419491
C 2.480678 -1.359553 -1.361350
C 4.732499 -1.146072 0.973383
C 6.018124 -0.675949 1.228994
C 6.202579 0.514440 1.929033
C 5.093350 1.235632 2.371755
C 3.808854 0.768761 2.115955
C 2.554813 0.011929 -1.630450
C 2.967792 0.459191 -2.882177
C 3.315013 -0.456079 -3.874782
C 3.245156 -1.823320 -3.610164
C 2.833068 -2.273473 -2.359554
N -0.341577 1.559647 1.081784
C -1.097873 2.560749 0.715160
C -2.217049 3.030037 1.401885
C -2.542125 2.361147 2.576490
C -1.765811 1.282622 2.989539
I -0.553910 3.528976 -1.134611
C -0.997178 -3.767126 0.177277
C -1.850320 -4.861283 0.321590
C -3.225842 -4.651712 0.266632
C -3.717897 -3.366442 0.070558
C -2.765862 -2.355742 -0.062212
N -1.470115 -2.520605 -0.015727
I -3.448384 -0.329614 -0.364955
H -0.161960 -0.836702 3.452988
H 0.850989 -4.940066 0.407179
H 4.591666 -2.075366 0.428371
H 6.876072 -1.241828 0.880097
H 7.204784 0.882074 2.124555
H 5.229420 2.168258 2.910070
H 2.942066 1.335418 2.445954
H 2.271155 0.725119 -0.860756
H 3.016934 1.525203 -3.081755
H 3.641181 -0.105835 -4.848831
H 3.518945 -2.541283 -4.376777
H 2.787421 -3.337722 -2.145809
H -2.798729 3.867420 1.038011
H -3.400234 2.676694 3.160962
H -2.003174 0.735523 3.895301
H -1.440792 -5.853712 0.474278
H -3.912988 -5.484268 0.376524
H -4.779215 -3.158435 0.023400

TS-R_aR_a-6
C 0.562080 1.963804 -0.458027
C 0.965190 0.665150 -0.396941
C 1.785836 -0.367732 -0.288755
C 1.009639 2.998015 -1.400415
C 2.232901 2.854378 -2.068920
C 2.654238 3.821316 -2.974988
C 1.861969 4.942497 -3.219935
C 0.640222 5.088367 -2.563046
C 0.211384 4.119084 -1.663211
C -0.562094 1.963786 0.458049
C -0.965153 0.665116 0.396970
C -1.785782 -0.367780 0.288787
C -1.009713 2.997982 1.400424
C -0.211579 4.119156 1.663142
C -0.640488 5.088415 2.562969
C -1.862182 4.942420 3.219929
C -2.654328 3.821135 2.975061
C -2.232923 2.854220 2.069001
C -3.197250 -0.261102 -0.112640
C 3.197304 -0.261047 0.112668
C -3.772669 0.931463 -0.568528
C -5.116840 0.938342 -0.914094
C -5.862303 -0.234157 -0.810551
C -5.178962 -1.358316 -0.359557
N -3.912209 -1.399973 -0.021514
N 3.912271 -1.399911 0.021511
C 5.179024 -1.358257 0.359556
C 5.862359 -0.234104 0.810577
C 5.116887 0.938387 0.914153
C 3.772715 0.931509 0.568590
I 6.266349 -3.215032 0.195330
I -6.266286 -3.215095 -0.195364
H 3.168028 1.828270 0.656057
H 2.850412 1.981364 -1.874777
H 3.603246 3.701705 -3.487311
H 2.193848 5.698843 -3.923765
H 0.016307 5.954264 -2.758877
H -0.748518 4.224329 -1.165612
H 1.421443 -1.374936 -0.478089
H 0.748283 4.224502 1.165489
H -0.016668 5.954396 2.758737
H -2.194114 5.698749 3.923752
H -3.603293 3.701424 3.487440
H -2.850338 1.981125 1.874920
H -3.167989 1.828232 -0.655966
H -5.587387 1.849380 -1.269086
H -6.912086 -0.271237 -1.071691
H 6.912142 -0.271183 1.071716
H 5.587428 1.849417 1.269171
H -1.421381 -1.374979 0.478127

TS-S_aS_a-6
C -3.435178 -0.661316 0.282002
C -2.118374 -1.020612 0.176283
C -1.143756 -1.900398 -0.028497
C -4.494731 -1.379348 1.003796
C -4.392355 -2.761825 1.204693
C -5.385980 -3.445856 1.896953
C -6.491656 -2.757068 2.394029
C -6.598164 -1.379386 2.202192
C -5.603905 -0.691933 1.515587
C -3.435180 0.661222 -0.282255
C -2.118341 1.020446 -0.176655
C -1.143707 1.900161 0.028357
C -4.494779 1.379316 -1.003914
C -5.604265 0.692040 -1.515216
C -6.598581 1.379569 -2.201665
C -6.491820 2.757182 -2.393845
C -5.385825 3.445826 -1.897277
C -4.392146 2.761725 -1.205167
C 0.246848 1.700543 -0.400084
C 0.246768 -1.700731 0.400027
C 0.589372 0.803455 -1.419559
C 1.928778 0.632456 -1.740602
C 2.897091 1.373791 -1.065914
C 2.432719 2.254711 -0.095647
N 1.177234 2.434548 0.239667
N 1.177234 -2.434640 -0.239720
C 2.432685 -2.254761 0.095690
C 2.896946 -1.373893 1.066061
C 1.928552 -0.632653 1.740734
C 0.589176 -0.803686 1.419575
I 3.882517 -3.423936 -0.994994
I 3.882410 3.424075 0.995024
H -0.189773 -0.247519 1.930210
H -3.531412 -3.296207 0.814013
H -5.299129 -4.517175 2.045799
H -7.267684 -3.291794 2.932178
H -7.453314 -0.839136 2.594930
H -5.679833 0.382825 1.378787
H -1.347969 -2.822647 -0.572017
H -5.680390 -0.382673 -1.378187
H -7.453976 0.839424 -2.594014
H -7.267896 3.291966 -2.931869
H -5.298767 4.517089 -2.046402
H -3.530955 3.295997 -0.814883
H -0.189514 0.247234 -1.930232
H 2.225073 -0.069998 -2.513675
H 3.952656 1.269009 -1.283418
H 3.952491 -1.269073 1.283641
H 2.224760 0.069767 2.513871
H -1.347956 2.822356 0.571956

TS-S_aR_a-6
C -0.939252 1.519442 0.942447
C -0.400349 2.512486 0.041453
C 0.934893 2.450199 0.303140
C 0.117363 0.673497 1.099953
C 0.713542 -0.508989 1.151925
C 2.125520 2.972139 0.571469
C 0.054343 -1.753556 0.729236
C -2.204188 1.535362 1.690704

C -1.070423 3.244722 -1.042429
C -2.646529 0.381577 2.351493
C -3.836258 0.399269 3.071383
C -4.599420 1.564890 3.135762
C -4.162399 2.718394 2.484923
C -2.968296 2.707304 1.772454
C -2.315617 2.817449 -1.522542
C -2.943942 3.509487 -2.551661
C -2.331229 4.626784 -3.119430
C -1.087001 5.050715 -2.653647
C -0.459306 4.365198 -1.618972
N -1.188931 -1.670480 0.213347
C -1.783091 -2.779933 -0.141711
C -1.242307 -4.059936 -0.049719
C 0.044148 -4.143568 0.475574
C 0.702371 -2.985124 0.871868
I -3.784438 -2.568560 -0.928496
C 3.379166 2.215213 0.591067
C 4.555802 2.791637 1.080798
C 5.723279 2.037307 1.092354
C 5.701211 0.726838 0.623741
C 4.472486 0.265155 0.158088
N 3.359925 0.950790 0.120960
I 4.353969 -1.761812 -0.581411
H 1.748252 -0.584492 1.474020
H 2.186070 4.026327 0.841302
H -2.058059 -0.527620 2.282620
H -4.171325 -0.499619 3.579062
H -5.530170 1.575339 3.693828
H -4.747958 3.630345 2.539919
H -2.620345 3.611142 1.280792
H -2.783365 1.937187 -1.090142
H -3.908380 3.172200 -2.917250
H -2.821314 5.164043 -3.924974
H -0.607159 5.918556 -3.094354
H 0.508014 4.694215 -1.250003
H -1.792277 -4.936211 -0.367649
H 0.526030 -5.110751 0.576176
H 1.703332 -3.022650 1.287663
H 4.546131 3.811547 1.449212
H 6.648340 2.462257 1.468173
H 6.584983 0.102037 0.618163

EZ-3
C 2.505736 -0.947502 0.060897
C 3.418471 0.072775 0.012099
C 2.365957 1.112580 0.038244
C 1.360586 -0.000309 0.170090
C 0.034325 -0.055600 0.357365
C 2.449786 2.445868 -0.102596
C -0.754736 -1.264645 0.630805
C 1.373715 3.434186 -0.136476
C 2.620685 -2.403953 -0.030298
C 4.871882 0.162327 -0.111035
C 1.710633 -3.136998 -0.803660
C 1.829405 -4.519223 -0.905385
C 2.850441 -5.184917 -0.228276
C 3.758748 -4.462684 0.545696
C 3.648588 -3.079478 0.642087
C 5.568058 1.199033 0.525774
C 6.951940 1.292380 0.408942
C 7.653607 0.358245 -0.350309
C 6.967518 -0.674679 -0.990586
C 5.586455 -0.775278 -0.871594
N -2.000292 -1.281769 0.107145
C -2.748848 -2.331504 0.334074
C -2.380799 -3.445597 1.084588
C -1.110610 -3.413006 1.650174
C -0.290587 -2.311078 1.433551
C 1.676806 4.790070 -0.311481
C 0.643283 5.718718 -0.342393
C -0.672869 5.289266 -0.199882
C -0.853593 3.918772 -0.031625
N 0.097652 3.022556 0.002216
I -4.727286 -2.293328 -0.530903
I -2.865577 3.166015 0.194981
H -0.521159 0.874323 0.308253
H 3.448549 2.858200 -0.228646
H 0.914722 -2.615754 -1.328493
H 1.122063 -5.077339 -1.510301
H 2.937885 -6.264086 -0.302434
H 4.552165 -4.978083 1.077227
H 4.351437 -2.514155 1.247467
H 5.024078 1.915875 1.133440
H 7.481802 2.094370 0.912555
H 8.732112 0.433836 -0.444266
H 7.510605 -1.401037 -1.586556
H 5.050216 -1.572753 -1.377246
H -3.053592 -4.280816 1.231087
H -0.768621 -4.240110 2.263890
H 0.692286 -2.250727 1.886124
H 2.709455 5.102487 -0.421201
H 0.857044 6.773981 -0.476996
H -1.508340 5.977271 -0.218309

ZZ-3
C 0.663698 0.008968 -0.183063
C -0.663689 0.008971 0.183238
C -0.729868 -1.471472 0.162897
C 0.729868 -1.471477 -0.162741
C 1.598885 -2.480091 -0.313878
C -1.598900 -2.480075 0.314008
C 3.048706 -2.355027 -0.489487
C 1.608032 1.071697 -0.533906
C -1.608034 1.071673 0.534099
C 2.469790 0.905335 -1.626512
C 3.324448 1.932106 -2.010719
C 3.339886 3.131878 -1.299671
C 2.496439 3.300147 -0.202642
C 1.631036 2.278065 0.177090
C -2.469910 0.905210 1.626598
C -3.324612 1.931938 2.010816
C -3.339984 3.131778 1.299883
C -2.496420 3.300154 0.202959
C -1.630973 2.278115 -0.176784
N 3.655537 -1.280614 0.054491
C 4.949217 -1.160037 -0.101684
C 5.773819 -2.057949 -0.774689
C 5.150246 -3.177807 -1.317669
C 3.776972 -3.335146 -1.172594
I 5.857083 0.577216 0.802596
C -3.048721 -2.354965 0.489600
C -3.777021 -3.335014 1.172765
C -5.150297 -3.177641 1.317792
C -5.773836 -2.057820 0.774696
C -4.949200 -1.159974 0.101646
N -3.655519 -1.280589 -0.054497
I -5.857028 0.577185 -0.802856
H 1.204102 -3.494044 -0.294250
H -1.204138 -3.494035 0.294360
H 2.453934 -0.030098 -2.178967
H 3.981958 1.795383 -2.863512
H 4.010814 3.931572 -1.597289
H 2.510417 4.229186 0.358361
H 0.971624 2.409034 1.030007
H -2.454097 -0.030268 2.178980
H -3.982215 1.795125 2.863523
H -4.010952 3.931438 1.597502
H -2.510337 4.229250 -0.357953
H -0.971485 2.409173 -1.029627
H 6.840684 -1.896588 -0.861174
H 5.736402 -3.919697 -1.850140
H 3.265418 -4.196854 -1.587432
H -3.265493 -4.196701 1.587679
H -5.736480 -3.919482 1.850299
H -6.840704 -1.896443 0.861120
H 7.57668900 1.65860900 -1.89777300

***EE*-3**
C 3.461323 -0.684873 0.034993
C 3.461330 0.684908 -0.034963
C 1.981922 0.744172 -0.115152
C 1.981917 -0.744121 0.115215
C 1.122803 -1.716469 0.450562
C 1.122797 1.716502 -0.450523
C -0.302507 -1.526187 0.762502
C -0.302517 1.526181 -0.762433
C 4.468313 -1.738712 0.114228

C	4.468327	1.738737	-0.114232
C	4.195357	-3.004256	-0.423309
C	5.141264	-4.021843	-0.342131
C	6.364307	-3.790043	0.284252
C	6.641279	-2.534924	0.828158
C	5.701679	-1.514370	0.744047
C	4.195371	3.004305	0.423250
C	5.141282	4.021885	0.342039
C	6.364330	3.790055	-0.284322
C	6.641303	2.534913	-0.828173
C	5.701699	1.514366	-0.744029
N	-1.149705	-2.467155	0.301800
C	-2.429493	-2.304699	0.538549
C	-2.997709	-1.249554	1.246109
C	-2.116227	-0.298073	1.751357
C	-0.753785	-0.438479	1.517690
C	-0.753790	0.438388	-1.517502
C	-2.116236	0.297923	-1.751113
C	-2.997726	1.249433	-1.245934
C	-2.429517	2.304658	-0.538484
N	-1.149726	2.467167	-0.301789
I	-3.745056	-3.818081	-0.259068
I	-3.745092	3.818107	0.258983
H	1.494645	-2.735064	0.534342
H	1.494629	2.735094	-0.534371
H	3.248526	-3.178998	-0.925730
H	4.922986	-4.995511	-0.768596
H	7.100194	-4.584871	0.350780
H	7.590151	-2.353569	1.322528
H	5.911035	-0.541102	1.178343
H	3.248535	3.179072	0.925655
H	4.923003	4.995572	0.768462
H	7.100220	4.584878	-0.350875
H	7.590180	2.353534	-1.322526
H	5.911056	0.541080	-1.178284
H	-4.067184	-1.178092	1.398853
H	-2.494130	0.543650	2.323654
H	-0.040626	0.282960	1.901367
H	-0.040626	-0.283077	-1.901119
H	-2.494137	-0.543869	-2.323310
H	-4.067202	1.177935	-1.398647

Supplementary Table S11. R_3C–H homolytic bond dissociation energies ($\Delta H_{BDE,homolytic}$).

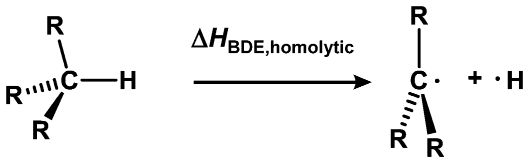

R^1 subtituent	R^2 subtituent	R^3 subtituent	BDE
H	H	H	103.0
CH_3	H	H	99.8
HC≡C	H	H	90.4
C_5H_4NC≡C	H	H	88.2
C_5H_3INC≡C	H	H	89.1
C_6H_5	H	H	88.7
CH_3	C_6H_5	HC≡C	77.9
CH_3	C_6H_5	C_6H_4NC≡C	76.2
CH_3	C_6H_5	C_6H_3INC≡C	76.9

Computed at (TightPNO)DLPNO-CCSD(T)/CBS(3,4/def2)//PCM(DMF)-M06-2X-D3/6-31G(d)/SDD.

References

[1] M. J. Frisch, G. W. Trucks, H. B. Schlegel, G. E. Scuseria, M. A. Robb, J. R. Cheeseman, G. Scalmani, V. Barone, G. A. Petersson, H. Nakatsuji, X. Li, M. Caricato, A. V. Marenich, J. Bloino, B. G. Janesko, R. Gomperts, B. Mennucci, H. P. Hratchian, J. V. Ortiz, A. F. Izmaylov, J. L. Sonnenberg, Williams, F. Ding, F. Lipparini, F. Egidi, J. Goings, B. Peng, A. Petrone, T. Henderson, D. Ranasinghe, V. G. Zakrzewski, J. Gao, N. Rega, G. Zheng, W. Liang, M. Hada, M. Ehara, K. Toyota, R. Fukuda, J. Hasegawa, M. Ishida, T. Nakajima, Y. Honda, O. Kitao, H. Nakai, T. Vreven, K. Throssell, J. A. Montgomery Jr., J. E. Peralta, F. Ogliaro, M. J. Bearpark, J. J. Heyd, E. N. Brothers, K. N. Kudin, V. N. Staroverov, T. A. Keith, R. Kobayashi, J. Normand, K. Raghavachari, A. P. Rendell, J. C. Burant, S. S. Iyengar, J. Tomasi, M. Cossi, J. M. Millam, M. Klene, C. Adamo, R. Cammi, J. W. Ochterski, R. L. Martin, K. Morokuma, O. Farkas, J. B. Foresman, D. J. Fox, **2016**.

[2] Y. Zhao, D. G. Truhlar, *Theor. Chem. Acc.* **2008**, *120*, 215–241.

[3] S. Grimme, J. Antony, S. Ehrlich, H. Krieg, *J. Chem. Phys.* **2010**, *132*, 154104.

[4] W. J. Hehre, R. Ditchfield, J. A. Pople, *J. Chem. Phys.* **1972**, *56*, 2257–2261.

[5] G. Scalmani, M. J. Frisch, *J. Chem. Phys.* **2010**, *132*, 114110.

[6] H. Stoll, B. Metz, M. Dolg, *J. Comput. Chem.* **2002**, *23*, 767–778.

[7] C. Gonzalez, H. B. Schlegel, *J. Chem. Phys.* **1989**, *90*, 2154–2161.

[8] R. Krishnan, J. S. Binkley, R. Seeger, J. A. Pople, *J. Chem. Phys.* **1980**, *72*, 650–654.

[9] T. Clark, J. Chandrasekhar, G. W. Spitznagel, P. V. R. Schleyer, *J. Comput. Chem.* **1983**, *4*, 294–301.

[10] A. V. Marenich, C. J. Cramer, D. G. Truhlar, *J. Phys. Chem. B* **2009**, *113*, 6378–6396.

[11] E. Engelage, N. Schulz, F. Heinen, S. M. Huber, D. G. Truhlar, C. J. Cramer, *Chem. – Eur. J.* **2018**, *24*, 15983–15987.

[12] P. Pracht, S. Grimme, C. Bannwarth, F. Bohle, S. Ehlert, G. Feldmann, J. Gorges, M. Müller, T. Neudecker, C. Plett, S. Spicher, P. Steinbach, P. A. Wesołowski, F. Zeller, *J. Chem. Phys.* **2024**, *160*, 114110.

[13] C. Bannwarth, S. Ehlert, S. Grimme, *J. Chem. Theory Comput.* **2019**, *15*, 1652–1671.
